# Development of a genome-wide multiple duplex-SSR protocol and its applications for the identification of selfed progeny in switchgrass

**DOI:** 10.1186/1471-2164-13-522

**Published:** 2012-10-03

**Authors:** Linglong Liu, Yanqi Wu

**Affiliations:** 1 Department of Plant and Soil Sciences, Oklahoma State University, 368 Ag Hall, Stillwater, OK, 74078-6028, USA

**Keywords:** SSR marker, Duplex PCR, Polymorphic information content, Selfing ratio, Switchgrass

## Abstract

**Background:**

Switchgrass (*Panicum virgatum*) is a herbaceous crop for the cellulosic biofuel feedstock development in the USA and Europe. As switchgrass is a naturally outcrossing species, accurate identification of selfed progeny is important to producing inbreds, which can be used in the production of heterotic hybrids. Development of a technically reliable, time-saving and easily used marker system is needed to quantify and characterize breeding origin of progeny plants of targeted parents.

**Results:**

Genome-wide screening of 915 mapped microsatellite (simple sequence repeat, SSR) markers was conducted, and 842 (92.0%) produced clear and scorable bands on a pooled DNA sample of eight switchgrass varieties. A total of 166 primer pairs were selected on the basis of their relatively even distribution in switchgrass genome and PCR amplification quality on 16 tetraploid genotypes. Mean polymorphic information content value for the 166 markers was 0.810 ranging from 0.116 to 0.959. From them, a core set of 48 loci, which had been mapped on 17 linkage groups, was further tested and optimized to develop 24 sets of duplex markers. Most of (up to 87.5%) targeted, but non-allelic amplicons within each duplex were separated by more than 10-bp. Using the established duplex PCR protocol, selfing ratio (*i.e.*, selfed/all progeny x100%) was identified as 0% for a randomly selected open-pollinated ‘Kanlow’ genotype grown in the field, 15.4% for 22 field-grown plants of bagged inflorescences, and 77.3% for a selected plant grown in a growth chamber.

**Conclusions:**

The study developed a duplex SSR-based PCR protocol consisting of 48 markers, providing ample choices of non-tightly-linked loci in switchgrass whole genome, and representing a powerful, time-saving and easily used method for the identification of selfed progeny in switchgrass. The protocol should be a valuable tool in switchgrass breeding efforts.

## Background

Switchgrass (*Panicum virgatum* L.) is a C4 perennial grass native to the prairies of North America and being developed as a herbaceous crop for the biofuel feedstock production in the USA and Europe [1-3]. Recurrent selection procedures have been widely employed in genetic improvement of populations and development of cultivars in switchgrass [4]. Because of its wind facilitated pollination behavior and low fertility of bagged inflorescences, switchgrass is considered to be allogamous. Although homozygous inbred lines of switchgrass had not been reported yet, some studies indicated that the rate of self-pollination ranged from less than 1% of bagged inflorescences [5-7] to higher than 50% in some specific individuals [8] or a genotype grown in a controlled environment [9]. Through continuous selfing, development of inbred lines is potentially possible, which will produce single cross hybrid cultivars in switchgrass [8-10]. Hybrid vigor (*i.e.*, biomass yield) has been reported in switchgrass [11]. Using tissue culture protocols to clonally propagate two heterozygous parents for hybrid seed production was suggested [11]. But the approach for producing hybrid cultivars is not applied due to high costs associated with producing large quantities of switchgrass clones through tissue culture and transplanting the clones into field plantings for large scale seed production. Identification of selfed progeny, offers the potential for the development of switchgrass inbred lines to serve as parents of F1 hybrids. The proposed procedure has proven successful for large amounts of seed production in maize (*Zea mays*), likely applicable in switchgrass.

The attempt to obtain switchgrass selfed seeds was carried out by bagging inflorescences of selected plants [5-7]. However, bagging may not be fully effective to prevent pollen contamination [12]. It has been proposed that seeds from bagged panicles needed to be genotyped with molecular markers to confirm parentage [8]. Morphological traits, such as pubescence on the adaxial surface of the leaf blade, foliage color, and seed size, were used to identify selfed and crossed progeny in previous experiments [13,14]. Although these phenotypic markers are simple and easily used, they are not only genotype-dependent but also may be environmentally sensitive. Instead, simple sequence repeat (SSR) markers have many advantages due to their co-dominance, low cost, high polymorphism, and environmental independence, and have been available in switchgrass [15-19]. SSR markers were used for genetic diversity [20], cultivar classification [21], and evolution [22] in switchgrass. Using PCR amplifications of six individual SSR markers, one preliminary study reported the confirmation of selfed progeny in switchgrass [12]. It will save 50% time and cost in the lab work if a duplex PCR protocol is developed. In addition, one prerequisite for molecular genetic-based inbred testing requires non-tightly linked markers [23]. Recently available SSR linkage maps [18,24] enable to select molecular markers covering much of the genome and less of linkage in switchgrass.

Multiplex polymerase chain reaction (PCR) consists of two or more primer sets within a single PCR mixture to produce amplicons of varying sizes that are specific to different DNA sequences. Since the use to detect deletions in a *dystrophin* gene [25], multiplex PCR protocols have been well established as a widespread technique in forensic studies, disease diagnosis, pathogen identification or linkage analysis due to obvious advantages of reducing labor, time, and reagent cost (see review [26]). In crops, to identify genotypes, test seed purity, and protect intellectual property, multiplex PCRs have been developed in rapeseed (*Brassica napus*) [27], cassava (*Manihot esculenta*) [28], peanut (*Arachis hypogaea*) [29], sorghum *Sorghum bicolor*[30], cotton (*Gossypium hirsutum*) [31], soybean (*Glycine max*) [32], maize (*Zea mays*) [33], sunflower (*Helianthus annuus*) [34], wheat (*Triticum aestivum*) [35,36], and red cover (*Trifolium pretense*) [37].

Because of inherent variation for selfing rates in switchgrass, development of a technically reliable and easily used multiplex marker system is very useful to quantify and characterize selfing and crossing rates of switchgrass. However, no similar study has been reported in switchgrass. The objectives of this study were: (1) to select a set of polymorphic SSR markers based on genome-wide screening, (2) to develop a duplex PCR-based protocol, and (3) to apply this SSR system in the identification of self- and cross-fertilized progeny of selected switchgrass plants in different growth conditions.

## Results

### Screening and evaluation of genome-wide mapped SSR markers

Of the 915 primer pairs (PPs) that were positioned on the published linkage maps [18,24], 842 (92.0%) produced clearly scorable bands with approximate sizes as reported previously [15-19]. The remaining 73 (8.0%) PPs produced either no amplicons or nonspecific or smear products. The number of alleles among the scorable SSR markers ranged from one to 20. The mean number of alleles per locus was 14.3 for dinucleotide, 10.5 for trinucleotide, and 8.3 for other SSR markers with repeat motifs ≥4 (Table 1). The SSRs with dinucleotide repeats produced a significantly greater number of alleles than those with trinucleotide repeats (*t*-test, *p* < 0.01).

**Table 1:** The evaluation of microsatellite primer pairs (PPs) for different repeat classes in pooled DNA

**Class**	**Primer pairs**			**Mean of alleles per locus ± standard error**
	**Tested**	**Scorable**	**Percentage (%)**	
Dinucleotide	409	384	93.9	14.3±0.3
Trinucleotide	369	342	92.7	10.5 ±0.2
Tetra-, penta-,hexa-nucleotide	29	20	69.0	8.3±0.7
Compound	108	96	88.9	10.1±0.3
Total	915	842	92.0	_

**Figure 1:**
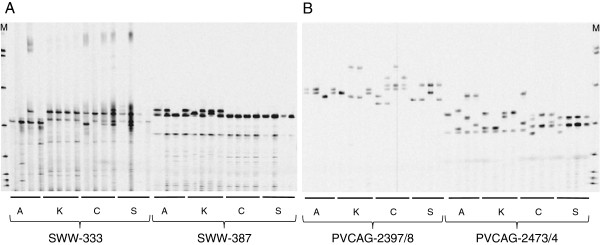
**Polymorphisms of SSR primer pairs in a panel of 16 individuals.** The panel was made from four cultivars, Alamo (A) Kanlow (K), Cimarron (C) and Summer (S), and each variety included four different genotypes. The lane with an “M” is DNA marker of 50-350 bp size standards (LI-COR Biosciences, Lincoln, NE, USA). **A)** Amplification from two EST-SSR markers, SWW-333 and SWW-387. **B)** Amplification from two genomic SSR markers, PVCAG-2397/8 and PVCAG-2473/4.

From the 842 PPs, 166 well amplified SSR markers were selected due to their relatively high allele number (≥4) per locus. These markers were distributed on 18 linkage groups (LGs) and spanned 1751.4 cM (84.0% coverage) of the reference map [24]. The number of SSR markers in each LG ranged from 2 on LG 7b to 20 on LG 3b ( Additional file 1). Average marker interval was 10.6 cM. The uncovered regions spanned 333.8 cM on the recent map, and those longer than 15 cM were on seven LGs, *i.e.*, LG 1b, 3a, 3b, 6a, 2a, 7a, and 9a ( Additional file 1). To estimate polymorphic information content (PIC), assess genotyping quality, and identify candidate SSR markers for multiple duplex PCR, the 166 SSR markers were tested for polymorphisms on 16 individuals selected from four different tetraploid cultivars (Figure 1). The raw data (*e.g.*, SSR allele size range, heterozygosity, PIC value, frequency of each allele, etc.) for each SSR marker are presented in Additional file 2.

**Figure 2:**
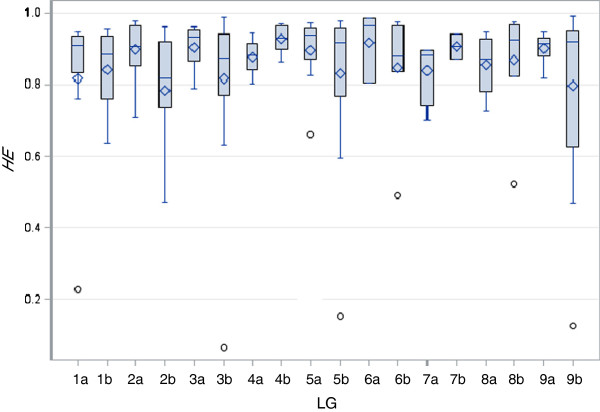
**Expected heterozygosity (*HE*) by linkage group (LG) for 166 simple sequence repeat markers.** The box plot showing means (*lozenge*) and distribution of heterozygosity values for different LGs.

The selected EST-SSR (eSSR) markers produced fewer bands (Figure 1A) than genomic SSR (gSSR) (Figure 1B). The mean number of alleles was 8.4 amplified for eSSRs while 14.6 for gSSRs. Mean PIC value for all 166 loci was 0.810 with a range from 0.116 (SWW-2377) to 0.959 (PVCA-1843/4). No significant differences in expected heterozygosity were observed among 18 LGs (*p* >0.05, Figure 2). The gSSRs had a significantly higher mean PIC value (0.844 ± 0.135) than that of eSSR (0.688 ± 0.183) (*t*-test, *p* <0.001).

### Development of a set of duplex PCR markers

**Figure 3:**
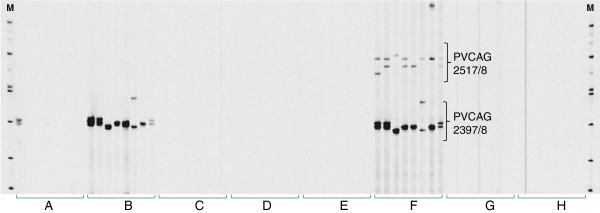
**Optimization of duplex PCR.** Alleles were amplified with a duplex set of SSR primer pairs (PVCAG-2517/8 and PVCAG-2397/8). Eight switchgrass genotypes from Alamo and Kanlow were used as amplification templates. The adjustments of PCR reaction components were shown from group A to H. Group **A**: control PCR with the same conditions as monoplex PCR, except for mixing PVCAG-2517/8 and PVCAG-2397/8 together; **B**: doubling *Taq* polymerase; **C**: doubling dNTPs concentration; **D**: doubling concentrations of DNA templates; **E**: increasing buffer concentration to 1.6x; **F**: increasing Mg ^2+^ concentration to 2.4 mM; **G**: doubling IR-M13 dye; **H**: doubling primer concentrations of PVCAG-2517/8 and PVCAG-2397/8. “M” indicated the DNA ladder.

Multiplex PCRs were developed by using the principles described by Edwards and Gibbs [26] and Hayden *et al.*[35,36]. PCR robustness, polymorphism and map position were used as the screening criteria to select 60 out of the 166 single-locus markers, and then these markers were empirically tested for duplex PCR quality. Comparing to monoplex PCR conditions, which generally didn’t work in duplex PCR by just combining two sets of primers together (Figure 3, set A), several adjustments associated with reaction chemicals were tested to optimize the protocol. The increase of dNTP, template DNA, buffer, IR-M13 dye, and primer concentrations did not significantly improve the amplification quality (Figure 3, set C, D, E, G and H). In contrast, the increase of *Taq* polymerase concentration from 0.25 to 0.5 units per 10*μ*l reaction partially increased amplicon quantity but did not correct uneven amplification or pull up unamplified alleles (Figure 3, set B). The most effective change that affected SSR primer compatibility was the increase in Mg^2+^ concentration (from 1× to 1.6×), which generally pulled up faintly amplified and unamplified loci (Figure 3, set F).

**Figure 4:**
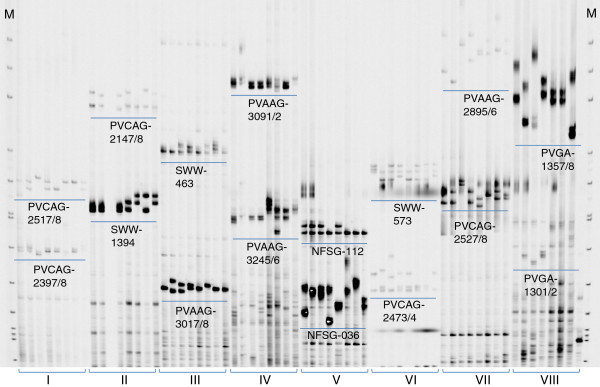
**Primer pair (PP) combinations for the duplex PCR.** The target bands for each PP (*underline*) and names of PPs are given. Here the duplex sets (I –VIII) were temporarily encoded and later they were integrated into the final list of duplex sets in Table 2. The DNA templates were the same as Figure 3.

After an optimal duplex PCR protocol was identified, individual SSR primer chemical regent quantities were modified as necessary to obtain appropriate fluorescent signals for two SSR markers in each duplex (Figure 4). The process of calibrating primer quantities was done by comparing fluorescent signal intensity. The relative ratio between two SSR PPs’ concentrations was more important than the absolute quantities, which is consistent with previous results [34]. The duplex PCR protocol required adding 0.125 to 4 pmoles of each SSR primer in a 10 *μ*l reaction volume (see details in Table 2).

**Table 2:** Parameters of 48 microsatellite markers assembled into 24 duplexes (Sets 1-24) in switchgrass

**Set**	**SSR ID**^**a**^	**Type**^**b**^	**Repeat motif**	**LG**^**c**^	**Position**^**d**^	**N**_**A**_^**e**^	**L**_**min-max**_**(bp)**^**f**^	**D**_**min**_**(bp)**^**g**^	**PIC**^**h**^	**NE-1P**^**i**^	**Primer** (**pmol)**^**j**^
1	PVAAG-3163/4	gSSR	(ACA)29	5b	63.5	19	211-293	16	0.925	0.248	1
	PVGA-1143/4	gSSR	(GA)7-(GA)8	5a	29.3	20	156-195		0.926	0.246	1
2	PVCAG-2397/8	gSSR	(CAG)12	3b	36.3	9	161-189	73	0.822	0.484	2
	PVCAG-2517/8	gSSR	(GCT)8	9a	6.9	15	213-234		0.879	0.362	2
3	PVCAG-2147/8	gSSR	(CAG)7	6b	150.5	12	285-306	68	0.836	0.454	1
	SWW-1394	eSSR	(GGT)n	7a,VIIa	60.4	8	194-217		0.651	0.709	0.5
4	SWW-463	eSSR	(TG)n	9a	58.3	7	253-273	108	0.762	0.58	0.5
	PVAAG-3017/8	gSSR	(AAG)11	6b	58.8	8	132-145		0.787	0.539	0.5
5	PVAAG-3091/2	gSSR	(TTC)13	9a	79.5	13	304-346	71	0.867	0.39	1
	PVAAG-3245/6	gSSR	(TTC)9	2a	91.4	19	102-233		0.929	0.237	0.25
6	NFSG-112	gSSR	(GA)n	8b,VIIIb	48	4	189-195	22	0.65	0.472	0.5
	NFSG-036	gSSR	(GA)n	4a,IVa	0	17	120-167		0.91	0.289	0.5
7	SWW-573	eSSR	(CAG)n	2b	39.5	11	229-247	60	0.767	0.561	1
	PVCAG-2473/4	gSSR	(GCA)12	3a	60.8	13	138-169		0.896	0.325	4
8	PVAAG-2895/6	gSSR	(GAA)10	5b	118	23	310-413	82	0.936	0.215	4
	PVCAG-2527/8	gSSR	(GCT)9	4b	60.8	12	206-228		0.867	0.392	4
9	NFSG-200	gSSR	(GA)n	9b,IXb	76.4	15	107-146	19	0.884	0.35	1
	NFSG-219	gSSR	(GA)n	8b,VIIIb	26.6	19	165-199		0.928	0.243	1
10	SWW-387	eSSR	(CT)n	9b	46.5	5	154-159	44	0.622	0.49	2
	PVGA-1663/4	gSSR	(AG)13	9b	34.1	17	203-230		0.89	0.332	2
11	PVAAG-3051/2	gSSR	(GAA)29	7a	31.8	15	185-254	10	0.851	0.418	1
	PVCAG-2209/10	gSSR	(GC)8	4a	13.4	9	264-274		0.839	0.452	1
12	PVCAG-2207/8	gSSR	(CTG)5	2b	40.3	7	221-238	29	0.677	0.685	0.125
	PVCAG-2289/90	gSSR	(TGC)5	4b	61	16	157-192		0.886	0.343	0.25
13	PVCAG-2187/8	gSSR	(GCA)7	8b	69.4	13	152-179	99	0.859	0.404	1
	PVGA-1549/50	gSSR	(GAA)6	1b	83.5	11	278-331		0.85	0.428	1
14	SWW-2662	eSSR	(AGG)n	2b, IIb	73.5	10	178-197	43	0.774	0.555	1
	5211_B07	eSSR	(AGC)8	2a	17.2	7	240-253		0.799	0.525	1
15	PVGA-1357/8	gSSR	(AC)7-(GA)22	5a	59.5	22	229-337	3	0.938	0.211	1
	PVGA-1301/2	gSSR	(TC)22	9b	50.5	17	163-226		0.914	0.279	1.5
16	PVGA-1243/4	gSSR	(TC)23	5b	69.3	25	280-317	62	0.945	0.191	2
	PVCAG-2297/8	gSSR	(CAG)6	3a	66.6	15	177-218		0.866	0.386	0.5
17	PVAAG-3311/2	gSSR	(CTT)28	2a	29.6	12	140-170	66	0.883	0.353	0.5
	PVGA-1813/4	gSSR	(GA)7	5a	72.2	18	236-276		0.915	0.277	1
18	NFSG-035	gSSR	(GA)n	3a, IIIa	129.2	13	117-151	52	0.897	0.325	1
	SWW-125	gSSR	(GA)n	2b, IIb	45.5	5	203-220		0.629	0.487	1
19	PVCA-893/4	gSSR	(AC)19	3b	65.3	9	297-336	81	0.78	0.546	1
	SWW-1615	eSSR	(GGC)n	1a, Ia	109.8	17	185-216		0.873	0.368	1
20	SWW-1622	eSSR	(GCG)n	2b, IIb	56.3	6	233-246	15	0.77	0.576	1
	SWW-1889	eSSR	(GCT)n	6b, VIb	72	4	211-218		0.624	0.484	1
21	PVCA-415/6	gSSR	(TG)16	6a	68.1	20	137-172	5	0.93	0.236	1
	SWW-1643	eSSR	(GA)n	3b	136	12	177-207		0.881	0.361	2
22	PVCA-979/80	gSSR	(GT)30	8a	50.2	8	283-310	60	0.678	0.679	2
	SWW-2376	eSSR	(CTG)n	5b, Vb	111.5	9	204-223		0.655	0.703	2
23	PVCAG-2269/70	gSSR	(CAG)8	4b	0	17	209-262	6	0.906	0.298	0.5
	PVCAG-2361/2	gSSR	(AGC)8	1b	25.9	8	268-277		0.705	0.648	1
24	PVCAG-2279/80	gSSR	(GCT)8	5a	11.7	11	236-248	14	0.83	0.46	0.25
	PVGA-1963/4	gSSR	(GA)9-(AG)6	5b	66.5	17	191-222		0.908	0.294	2

Footnote: ^a^ The sequences of primer pairs are available in previous studies [15-19]; ^b^ gSSR, genomic SSR; eSSR, EST-SSR; ^c^ The linkage group (LG) with an Arabic number indicates the map developed by Liu *et al.*[24], while Roman number by Okada *et al.*[18]; ^d^ The position indicated genetic distance of linkage map by Liu *et al.*[24]; ^e^ Number of alleles (N_A_); ^f^ Minimum and maximum allele lengths (L); ^g^ Minimum difference (D) between non-allelic bands; ^h^ Polymorphic information content (PIC); ^i^ Average non-exclusion probability for one known parent (NE-1P); ^j^ Each primer quantity.

Of the 60 tested PPs, 48 SSR markers were assembled into 24 duplexes (set #1-24) by testing them on eight individual DNA samples (Table 2). Twelve markers were discarded due to unsatisfactory amplifications in duplex PCRs. All duplex PCRs produced the same SSR alleles as monoplex PCRs (Figure 4). The 48 SSR markers represented 15 perfect and five compound repeat SSRs of dinucleotide, 26 perfect and one compound repeat SSRs of trinucleotide, and one compound repeat SSR of pentanucleotide (Table 2). The mean PIC for dinucleotide and trinucleotide SSRs were 0.823 and 0.820, respectively (Table 2). Eleven markers were eSSRs and the other 37 gSSRs (Table 2).

The 48 markers constituting the 24 duplexes are distributed on 17 LGs and the number of SSR markers per LG ranged from one (on LG 1a and 8a, respectively) to five (on LG 2b and 5b, respectively), based on a published linkage map [24] (see Additional file 1, in red). The mean distance of two immediate neighboring markers was 37.7 cM, and the nearest markers were on LG 2b with a mean distance of 8.5 cM. The only LG with no SSR marker loci contributing to the duplex PCR sets was LG 7b, one of the shortest and least polymorphic LGs in the mapping population [24].

The allele band size range of the 48 SSR markers was from 102 bp (PVAAG-3245/6) to 413 bp (PVAAG-2895/6) (Table 2). Non-allelic but targeted bands within each duplex were separated by more than10-bp among the 16 genotypes, aside from a 3-bp gap between alleles amplified by one duplex Set 15 (PVGA-1357/8 and PVGA-1301/2), a 5-bp gap by Set 21 (PVCA-415/6 and SWW-1643) and a 6-bp gap by Set 23 (PVCAG-2269/70 and PVCAG-2361/2) (Table 2).

The minimum, mean and maximum PIC values of the 48 markers were 0.622, 0.829 and 0.945, respectively. The mean of non-exclusion probability of one marker, if one parent is known (NE-1P), were 0.414 ranging from 0.191 to 0.709 (Table 2).

### Validation of the duplex PCR in identification of selfed progeny using different populations

**Figure 5:**
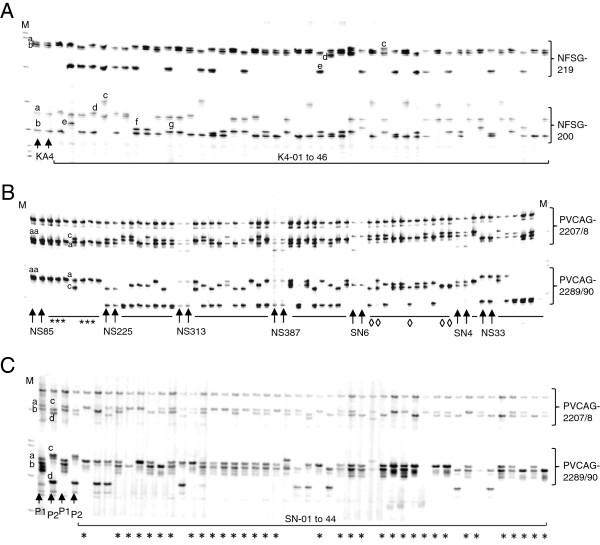
**Genotyping switchgrass progeny with duplex PCR.** For each parent, DNA sample was duplicated in PCR and shown by arrows. The letters (“a” to “g”) indicate different alleles of each locus. “M” indicates the DNA ladder. **A**) Genotyping a maternal plant (K4) grown in a field plot and its 46 progeny (K4-01 to 46) with a duplex set 9 (NFSG-200 and NFSG-219). **B**) Genotyping seven parents (*arrows*) and their progeny harvested by bagging in the field with duplex set 12 (PVCAG-2207/8 and PVCAG-2289/90). The asterisks indicate selfed progeny and lozenges indicate seed contaminations. **C**) Genotyping the seed parent ‘SL937x15’ (P1), pollen parent ‘NL94 LYE 16x13’ (P2) and 44 progeny (SN-01 to 44) amplified with a duplex set 12. The asterisks indicate selfed progeny.

**Figure 6:**
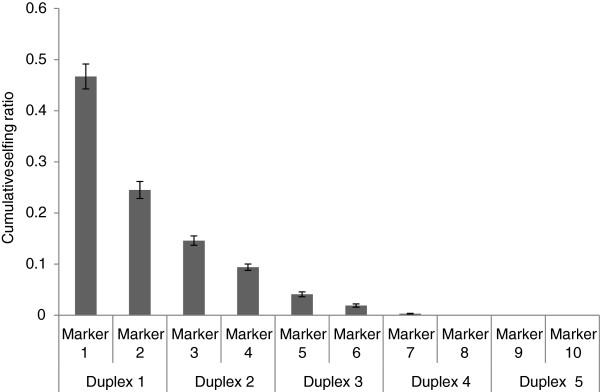
**Cumulative selfing ratio with increasing the number of loci.** The duplex sets were randomly selected on different linkage groups. The bar indicated standard error.

Three populations produced in different environments were used to determine selfing ratios (Figure 5). Population 1: A maternal plant (K4) grown in the field condition and its 46 putative half-sib progeny were genotyped with 100 sets of randomly selected duplex markers (Figure 5A). Each set contained five duplexes with 10 loci on different LGs. One plant (K4-11) was identified as a contaminant because all alleles of its six loci (PVCAG2361/2, 5211-B07, PVAAG3163/4, PVGA1143/4, PVCAG2397/8 and PVGA1963/4) were not inherited from K4. The remaining 45 plants showed the maternity relatedness with K4 and were included in further analysis. The results showed that, if genotyping with one random marker, the mean value of selfing ratio was 46.7%. As more markers were tested and more polymorphisms were detected between the maternal plant and unknown pollen parents, the cumulative selfing ratio began to decline. When the number of marker loci increased up to eight or more, the cumulative selfing ratio dropped to 0 (Figure 6).

Population 2: Twenty-two different families with totally 99 progeny were genotyped for 10 loci with five sets of duplex PCR (Sets 1, 9, 12, 14 and 18). These progeny plants were from seeds harvested in a field plot by bagging panicles. Unexpectedly, of 99 progeny, 34 (34.3%) were identified as contaminants because each of them showed no alleles from their corresponding maternal parents in at least two loci (Figure 5B, indicated by lozenges). In the remaining 55 progeny, 10 plants from four families (*i.e.*, NS85-1, 2, 3, 5, 6, and 7; SN16-1 and -2; SN17-1; and SN44-1) shared the same alleles from their respective maternal parents, and therefore were identified as selfed progeny (Figure 5B, indicated by asterisks). The other 55 progeny were identified as hybrids due to their possession of alleles, which were different from their seed parents (Table 3). Later, the 10 selfed progeny were further confirmed by detecting two additional SSR duplexes (Sets 2 and 24). The overall selfing ratio by the bagging method was only 15.4% (10/65) if contaminants were excluded.

**Table 3:** Selfing test for plants by bagging with duplex PCRs

**Grand**	**Parent**	**Family**	**Selfed**	**Crossed**	
**parent**		**size**	**progeny**	**progeny**	**Contaminants**
NL94 LYE 16x13	NS33	6	0	6	0
	NS81	8	0	7	1
	NS85	7	6	1	0
	NS225	7	0	0	7
	NS313	10	0	10	0
	NS387	8	0	7	1
SL93 7x15	SN4	1	0	0	1
	SN6	11	0	1	10
	SN9	2	0	0	2
	SN11	7	0	1	6
	SN16	2	2	0	0
	SN17	1	1	0	0
	SN18	1	0	1	0
	SN19	1	0	0	1
	SN25	4	0	4	0
	SN30	1	0	1	0
	SN31	1	0	1	0
	SN33	5	0	3	2
	SN34	13	0	11	2
	SN38	1	0	0	1
	SN41	1	0	1	0
	SN44	1	1	0	0
Total		99	10	55	34

Population 3: Forty-four progeny plants from seed of a breeding line (‘SL93 7x15’, abbreviated as SL93) grown in a controlled growth chamber, in which another breeding line (‘NL94 LYE 16x13’, NL94) was grown as a potential pollen donor, were genotyped with five sets of PCR-duplex (Sets 1, 11, 12, 16 and 24, Figure 5C). The genotyping results were consistent across all 10 SSR loci. For the 44 progeny from SL93, 34 (77.3%) were identified as originating from self-fertilization and 10 (22.7%) were hybrids between SL93 and NL94. SL93, like NL94 as reported recently [9], was a self-compatible genotype in the specific environment.

## Discussion

Switchgrass has become one important energy crop in the USA and Europe due to its high biomass yield and adaptability on marginal lands, low nutrient and water requirements, and powerful ability as a carbon sink [1-3,8]. The duplex PCRs consisting of 48 SSR markers were the first developed in switchgrass. The protocol was based on a genome-wide selection of SSR marker loci. PCR-multiplexes for genome-wide or nearly genome-wide SSR marker loci have only been developed in limited plant species thus far, including maize [33], sunflower [34], *Arabidopsis thaliana*[38], rice [39] and soybean [40]. In other crops, such as rapeseed [27], cotton [31], and sorghum [30,41], multiplex PCR systems have been established, although markers were selected not covering the whole genome.

### SSR marker polymorphism

Initially, DNA samples from eight diverse switchgrass cultivars were pooled together, which not only kept the diversity of different ecotypes of switchgrass but simultaneously minimized the number of genotypes used for the preliminary screening of SSR markers. Similar strategy had been used in a previous switchgrass study [19]. Generally, SSR markers with dinucleotide repeats were more polymorphic than trinucleotide repeats in several plant species, such as barley [42], rice [43], wheat [44], maize [45], and soybean [46]. In this study, of all scorable PPs in the preliminary screening, the dinucleotide repeat SSRs produced a significantly greater number of alleles than those with trinucleotide repeats (Table 1), which was consistent with previous studies in switchgrass [19,24,47]. However, in the final 48 PPs selected for duplex PCR, we found that both classes of SSRs were equally polymorphic. The PIC values for the selected dinucleotide and trinucleotide SSRs were not significantly different (0.823 vs. 0.820), and the number of alleles per locus were nearly identical (12.3 for dinucleotide and 12.6 for trinucleotide repeats) (Table 2).

### Duplex PCR

Due to the competition of primers, DNAs, Mg^2+^, and other reaction components, PCR multiplexing generally requires some optimization [26]. In this study, we found increasing *Taq* and Mg^2+^ concentrations improved the duplex PCR quality (Figure 3). Previous studies showed that a *Taq* DNA polymerase concentration (with an appropriate increase in MgCl_2_ concentration) four to five times greater than that required in monoplex PCR, was necessary to achieve optimal nucleic acid amplification [48]. In contrast, the alteration of other PCR components such as PCR buffer constituents, dNTPs, and primer absolute concentrations in multiplex PCR over those reported for most monoplex PCRs usually resulted in little improvement in the sensitivity or specificity of the test [49]. But another study showed that only increasing the buffer concentration markedly improved the quality of multiplex PCR [34]. It is evident that the optimization is necessary in developing multiplex PCR [26,35,36].

Aside from technical factors discussed above, the selection of SSR markers to create PCR duplexes in switchgrass also integrated information of marker map position, allele-length range, genotyping quality, and polymorphism. The 48 selected markers covered the major portion of switchgrass genome based on the available genetic maps although they were not evenly distributed in the genome. Despite that we tried to select unlinked markers before designing duplex combinations, the selected marker loci in LG 2b and 5b remained linked although most of them >10 cM. Tightly linked loci are not ideal in paternity analysis because they are usually inherited together, but these markers indeed provide more choices, especially in case some of them lack polymorphisms within and between tested parents.

The band-size separations of individual SSR markers in each duplex combination were mostly more than 10-bp, which should be wide enough to unequivocally score alleles amplified in major switchgrass lowland varieties. Even for the nearest distance of non-allelic bands (3-bp, duplex Set 15), it could be easily differentiated on frequently-used polyacrylamide gels [50]. The duplex marker system might also perform well on capillary electrophoresis instruments due to their similar resolutions with the LI-COR DNA analyzer used here [50].

### SSRs needed for parentage analysis

A comprehensive review of 53 articles showed an average of seven microsatellites (ranging 3 to11) was used for plant parentage studies [51]. In general, the number of markers required to resolve parentage with a given level of confidence depends on a number of factors. One of the main factors is the expected heterozygosity or polymorphism of each marker [52]. Of the 48 markers, the mean PIC value was 0.829. In an actual example (population 1) with 46 individuals derived from naturally wind pollination, four sets of randomly selected duplexes containing eight markers were enough to discriminate the breeding origin of each progeny (inbreeding vs. outcrossing). In another population (population 3) harvested from a control environment, theoretically only one polymorphic locus could assign parentage to each progeny plant. Therefore, four duplex sets identified in this study would be recommended for the identification of self- or cross-fertilized progeny. Thus, the 24 sets of duplex SSRs should provide a reservoir used for the breeding origin analysis in switchgrass.

In addition, from population 2, using 14 markers, we found the overall selfing ratio of switchgrass plants by bagging their inflorescences was 15.4%, which was slightly lower than the results in a previous report [12]. The bagging method with pillow cases did not produce only selfed progeny, perhaps because openings on pillow cases were bigger than switchgrass pollen grains. Previous study showed pollen size of switchgrass was in the range of 42.5 to 54.0 *μ*m [53]. Therefore, bagging methods need to be improved if a large number of inbreds are needed in a hybrid breeding program in the future.

### Implications in other forage species

This study was focused on the tetraploid switchgrass varieties/genotypes. But paternity testing for polysomic genomes is complicated and has not yet been well developed [37]. Recent studies indicated that tetraploid switchgrass exhibited disomic inheritance [9,18,24]. So paternity testing for selfing analysis in switchgrass can be implemented by the same ways as diploid species with diploid-specific software (such as Cervus 3.0 [54]). The set of SSR markers selected here are also helpful to identify genotypes, test seed purity, and protect intellectual property in switchgrass. Other forage species with diploid and allopolyploid genome structure were reported as well, including creeping bentgrass (*Agrostis palustri*) [55], colonial bentgrass (*Agrostis capillaries*) [56], perennial ryegrass (*Lolium perenne*) [57], Italian ryegrass (*L. multiflorum*) [57], tall fescue (*Festuca arundinacea*) [58], meadow fescue (*Festuca pratensis*) [59], white clover (*Trifolium repens*) [60], red clover (*Trifolium pretense*) [61], and smooth bromegrass (*Bromus inermis*) [62]. Similar testing can be expanded into these species.

## Conclusions

Based on the genome-wide screening of a large set of SSR markers, we developed a multiple duplex PCR system including 48 polymorphic PPs. The applications of this SSR test system demonstrated its high discrimination capability and effectiveness for the identification of switchgrass selfed progeny, which were produced on multiple plants in different pollination conditions. The protocol provides ample SSR markers, which should be a powerful tool for the detection of inbreds in switchgrass.

## Methods

### Plant material

Plants of ‘Alamo’, ‘Kanlow’, ‘Nebraska 28’, ‘Cave-in-Rock’, ‘Summer’, ‘Docotah’, ‘Shelter’, and ‘Blackwell’ [63], were grown in an Oklahoma State University (OSU) greenhouse, Stillwater, OK. These cultivars had been widely used in the USA and represented eco- and cyto-type diversity within the species [64]. For initial SSR marker screening, equimolar DNAs from the eight cultivars were mixed to form a pooled DNA sample. For each cultivar, DNA sample was a mix from four to six plants.

In an OSU switchgrass nursery, ‘Alamo’, ‘Kanlow’, and ‘Cimarron’ plants were space planted on 3.5 feet × 3.5 feet centers in 2008. Four individuals from each cultivar were randomly selected. These 12 individual plants with additional four ‘Summer’ plants grown in the greenhouse constituted a panel (totally 16 plants), which was used for marker polymorphism analysis.

Open-pollinated seeds from a randomly selected ‘Kanlow’ genotype (encoded K4) in the nursery were harvested in 2010, and then they were germinated on filter paper in petri dishes after pre-chilling treatment for two weeks [65]. The obtained seedlings were transplanted into conetainers and grown in the greenhouse for leaf collection. The obtained half-sib progeny population (Population 1) of 46 individuals (encoded as K4-1 to 46) was used to examine selfing and outcrossing rates of a plant grown in the open-pollinating, natural field condition.

In 2010, 22 first-generation selfed (S1) plants of two genotypes NL94 and SL93 [9] were selected according to spring growth vigor, plant height, and crown size. And then two inflorescences from each plant were bagged with pillow cages [66] in the field before inflorescences fully emerged out. The obtained seeds were germinated respectively in a growth chamber in the spring of 2011. Survived plants were transplanted in a field plot on August 1, 2011 and constituted 22 families of totally 99 progeny plants (Population 2). The family size ranged from 1 to 13 in Population 2. Population 3 included 44 progeny collected from SL93 in a growth chamber, in which NL94 served as the pollen donor [9]. Genomic DNA was isolated from healthy leaf tissues using the CTAB method [67]. The DNA concentration was measured using an ND1000 spectrophotometer (NanoDrop Products, Wilmington, DE). The working solutions were adjusted to10 ng/*μ*l as PCR templates.

### SSR primer screening

PCR PPs were obtained principally from two sources: those on two-sister linkage maps (totally 585 PPs) [18], and the others from a recent linkage map (totally 473 PPs) [24]. Primer sequence information was collected from previous studies [15-19]. After excluding SSR redundancy, unique PPs were used in this study. All forward primers were appended with a M13 sequence (5’-CACGACGTTGTAAAACGAC-3’) at the 5’ end to allow indirect labeling in PCR reactions. The initial screening for polymorphism was performed using the pooled DNA sample to determine the number of alleles at each locus. Candidate SSR PPs were selected based on the previous data generated in the mapping experiment [24] and screening results of amplifying clear bands, displaying four or more alleles per locus and avoiding tight linkages (*i.e.*, >10 cM between two neighboring loci). And then selected PPs were tested on the panel with 16 individual DNA samples to determine polymorphism. Monoplex PCR with 10 *μ*l volume mixtures each reaction and a ‘Touchdown’ thermal cycling program was used [68,69].

### Development and optimization of duplex PCR

Based on amplified allele size, map position and heterozygosity for each of the candidate SSR markers, a smaller set of SSRs was selected for testing in duplex PCR. The criteria used to combine SSR markers into duplex PCR were as the following: (1) Non-overlapping allele size for each pair of two markers; (2) Primer compatibility and genotyping quality in duplex PCR; (3) High polymorphism estimated by PIC value [70]; (4) Two high-quality bands in each genotype due to disomic inheritance identified in tetraploid switchgrass [9,18]; (5) Genetic distance between selected SSRs ≥ 10 cM; (6) SSRs with tri-, tetra-, or higher nucleotide repeats were preferred to lessen slippage during PCR [71].

An optimization procedure was carried out before the final PP combinations for duplex PCR were assembled. Eight switchgrass genotypes from Alamo (A) and Kanlow (K), *i.e.*, A2, A4, A5, A10, K1, K3, K4 and K5, were used as amplification templates. The SSR PPs used here to optimize duplexes were PVCAG-2397/8 and 2517/8. The adjustment of duplex PCR parameters on the amplification effect followed: increasing *Taq* polymerase (BioLabs®, Catalog #M0273X, NEW ENGLAND Inc., USA) from 0.25 to 0.5 units, dNTPs from 0.2 to 0.4 mM, template DNAs from 15 to 30 ng, PCR buffer from 1 × to 1.6 ×, Mg ^2+^ concentration from 1.5 to 2.4 mM, IR-M13 forward primer (labeled with either 700 nm or 800 nm florescence) concentration from 0.02 to 0.04 μM and PP quantity from 1.0 to 2.0 pmoles. Subsequently, the compatibilities of different SSR primer combinations were tested on the same eight genotypes.

Duplex PCRs were performed in 10 *μ*l of reaction mixture containing 1 × PCR buffer, 2.4 mM of Mg ^2+^, 0.2 mM each of dNTPs, 0.125 to 4.0 pmoles of each primer (Table 2), 0.5 units of *Taq* polymerase (BioLabs®, USA), 0.02 μM IR-M13 forward primer, and 15 ng of genomic DNA. The cycling parameters were the same as monoplex PCR mentioned above. PCR products labeled with 700 and 800-nm dye were pooled, and mixed thoroughly. After denaturation, they were separated using 6.5% KB plus polyacrylamide gels with a LI-COR 4300 DNA Analyzer (LI-COR Biosciences, Lincoln, NE, USA) [69].

### Genotyping and data analysis

The gel bands were visually scored and band sizes were determined using Saga Generation 2 software, version 3.3 (LI-COR Biosciences, Lincoln, NE, USA). For Population 3, the scoring of PCR bands and identification of selfed progeny were the same as our former study [9]. For Populations 1 and 2, PCR bands were recorded as “ab” if two bands (“a” indicated upper band and “b” for lower band) or “aa” if only one band for the parent. In the progeny, if bands were from parents, they were scored in the same way as described above. If different bands were scored in the progeny, letters from “c” to “g” (or “h”, “i” and so on, if more alleles appeared) were assigned to each band based on the molecular sizes (“c” for the largest and “d” for a smaller band than “c” but larger than other non-parental bands) . If the alleles of a tested individual were all derived from its corresponding maternal parent, it was identified as a selfed progeny. Apart from maternal allele(s), if non-parental alleles of a progeny individual appeared, it was identified as cross-fertilized (*i.e.*, hybrid). If none of the alleles of a tested progeny was from the seed parents on more than two marker loci (≥2), it was determined as contaminants and excluded in further study.

*T*-test was carried out using Microsoft® Excel 2007. The allele frequency, heterozygosity, PIC, and average non-exclusion probability for one known parent (NE-1P) were estimated using Cervus 3.0 [54]. The output options were set as the following: Header row = yes, Read locus names = yes, First allele in column =3, Number of loci=166. For comparisons of expected heterozygosity among 18 LGs, the Scheffe’s method for general linear model (GLM) procedure was used in SAS 9.3 (SAS Institute Inc.) with a significance level of 0.05.

## Competing interests

The authors declare that they have no competing interests.

## Authors’ contributions

YQW conceived and designed the experiment, prepared the materials, supervised entire study and helped draft the manuscript. LL participated in the experimental design, carried out the field and molecular genetic studies, analyzed the data and drafted the manuscript. All authors read and approved the final manuscript.

## Supplementary Material

Additional file 1 **Linkage map of switchgrass showing the positions of 166 simple sequence repeat (SSR) marker loci for polymorphism analysis.** The genetic distances and marker orders are adopted from a previous study [24]. To simplify and clarify the display of linkage map in this study, only 166 loci are shown and the other 333 loci are removed from a previous reference map [24]. The loci that assembled into 24 duplex sets are indicated in red. Click here for file

Additional file 2 Allele frequency for 166 simple sequence repeat (SSR) markers. Click here for file
