# Supplementary figures and images for: Development of a genome-wide multiple duplex-SSR protocol and its applications for the identification of selfed progeny in switchgrass
Source: BMC Genomics. 2012 Oct 3;13:522. doi: 10.1186/1471-2164-13-522 (PMC3533973; doi:10.1186/1471-2164-13-522)

## Slide 1
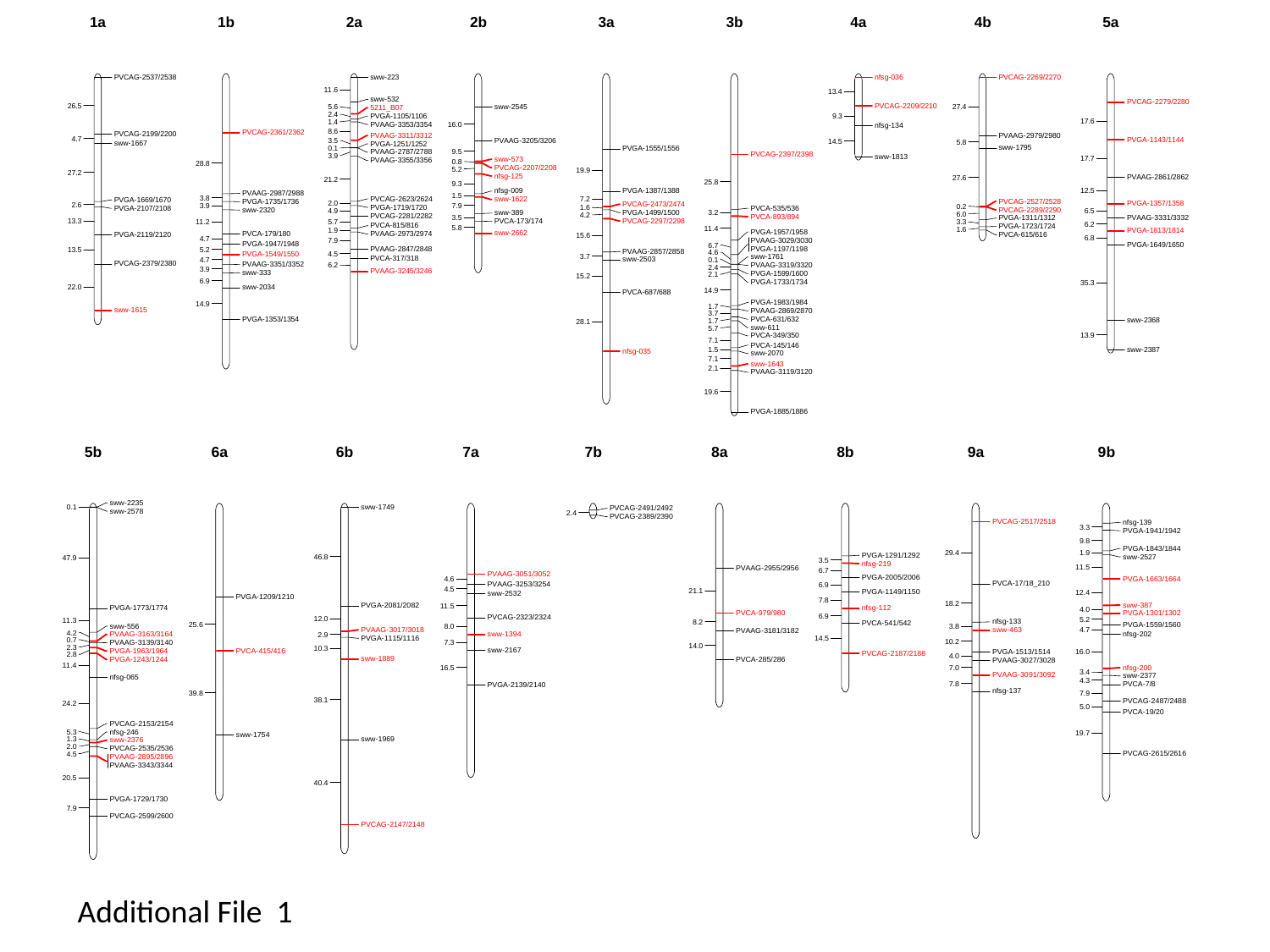

# Additional File 1

Supplement: Additional file 1 — Linkage map of switchgrass showing the positions of 166 simple sequence repeat (SSR) marker loci for polymorphism analysis. The genetic distances and marker orders are adopted from a previous study [24]. To simplify and clarify the display of linkage map in this study, only 166 loci are shown and the other 333 loci are removed from a previous reference map [24]. The loci that assembled into 24 duplex sets are indicated in red. [file 1471-2164-13-522-S1.pptx]
