# Supplementary material for: Development of a genome-wide multiple duplex-SSR protocol and its applications for the identification of selfed progeny in switchgrass
Source: BMC Genomics. 2012 Oct 3;13:522. doi: 10.1186/1471-2164-13-522 (PMC3533973; doi:10.1186/1471-2164-13-522)
Supplement: Additional file 2 — Allele frequency for 166 simple sequence repeat (SSR) markers. [file 1471-2164-13-522-S2.docx]

Cervus 3.0.3 - © Copyright Tristan Marshall 1998-2007

Distributed by Field Genetics Ltd - www.fieldgenetics.com

Licensed for non-commercial use only

Allele frequency analysis completed on Thursday, July 5, 2012, at 08:55 am

**** Summary statistics ****

Locus k N HObs HExp PIC NE-1P NE-2P NE-PP NE-I NE-SI

1105/1106 22 15 1.000 0.979 0.944 0.195 0.108 0.020 0.005 0.278

1115/1116 19 15 0.933 0.963 0.927 0.244 0.139 0.032 0.009 0.287

1149/1150 15 16 0.813 0.927 0.890 0.337 0.203 0.064 0.018 0.305

1209/1210 23 14 1.000 0.987 0.949 0.179 0.098 0.017 0.005 0.275

1243/1244 25 16 1.000 0.978 0.945 0.191 0.106 0.019 0.005 0.278

1251/1252 21 16 1.000 0.964 0.930 0.234 0.133 0.029 0.008 0.285

1291/1292 7 15 0.800 0.823 0.766 0.578 0.400 0.218 0.072 0.370

1301/1302 17 16 1.000 0.950 0.914 0.279 0.162 0.043 0.012 0.293

1311/1312 21 15 0.933 0.970 0.934 0.222 0.125 0.027 0.007 0.283

1353/1354 3 16 1.000 0.637 0.544 0.810 0.667 0.518 0.220 0.496

1357/1358 22 16 1.000 0.972 0.938 0.211 0.118 0.024 0.006 0.281

1387/1388 16 16 0.875 0.865 0.826 0.459 0.295 0.114 0.038 0.341

1499/1500 18 16 0.938 0.962 0.927 0.244 0.139 0.033 0.009 0.286

1513/1514 16 15 0.867 0.929 0.891 0.333 0.200 0.060 0.018 0.306

1549/1550 11 16 1.000 0.891 0.850 0.428 0.270 0.107 0.032 0.326

1555/1556 19 16 1.000 0.958 0.923 0.254 0.146 0.035 0.010 0.289

1559/1560 15 16 1.000 0.921 0.883 0.353 0.214 0.071 0.021 0.309

1599/1600 14 16 0.938 0.800 0.762 0.562 0.380 0.171 0.064 0.378

1649/1650 20 16 1.000 0.970 0.936 0.219 0.123 0.026 0.007 0.282

1663/1664 17 16 0.750 0.925 0.890 0.332 0.199 0.058 0.017 0.306

1669/1670 2 16 0.250 0.226 0.195 0.976 0.903 0.833 0.634 0.799

1719/1720 21 16 0.938 0.978 0.945 0.194 0.107 0.020 0.005 0.278

1723/1724 22 16 1.000 0.966 0.932 0.227 0.129 0.027 0.008 0.284

1733/1734 11 14 0.357 0.881 0.833 0.457 0.295 0.125 0.039 0.335

1735/1736 18 16 1.000 0.954 0.919 0.267 0.154 0.039 0.011 0.291

1773/1774 22 16 1.000 0.966 0.932 0.227 0.129 0.027 0.008 0.284

179/180 16 15 0.867 0.938 0.900 0.315 0.187 0.056 0.016 0.301

1813/1814 18 16 0.938 0.950 0.915 0.277 0.161 0.042 0.012 0.293

1843/1844 28 16 1.000 0.992 0.959 0.147 0.079 0.011 0.003 0.270

1941/1942 8 15 0.933 0.802 0.752 0.590 0.407 0.211 0.074 0.381

1947/1948 20 16 1.000 0.956 0.921 0.258 0.148 0.036 0.010 0.290

1957/1958 28 16 1.000 0.990 0.957 0.153 0.083 0.012 0.003 0.271

1963/1964 17 16 0.688 0.944 0.908 0.294 0.173 0.048 0.014 0.296

1983/1984 9 16 0.625 0.720 0.669 0.688 0.502 0.294 0.119 0.431

2005/2006 23 16 1.000 0.974 0.941 0.204 0.114 0.022 0.006 0.280

2081/2082 22 16 1.000 0.974 0.940 0.205 0.115 0.023 0.006 0.280

2107/2108 17 15 1.000 0.949 0.912 0.284 0.166 0.045 0.013 0.294

2119/2120 17 16 0.875 0.948 0.912 0.283 0.165 0.044 0.012 0.294

2139/2140 12 15 0.933 0.883 0.841 0.440 0.280 0.109 0.034 0.332

2147/2148 12 16 0.500 0.879 0.836 0.454 0.292 0.123 0.038 0.334

2153/2154 17 16 1.000 0.958 0.923 0.256 0.147 0.036 0.010 0.289

2187/2188 13 15 1.000 0.901 0.859 0.404 0.253 0.094 0.028 0.322

2199/2200 11 16 1.000 0.907 0.867 0.394 0.244 0.091 0.027 0.317

2207/2208 7 15 0.933 0.736 0.677 0.685 0.502 0.304 0.117 0.424

2209/2210 9 15 0.733 0.885 0.839 0.452 0.289 0.123 0.037 0.332

2269/2270 17 16 1.000 0.942 0.906 0.298 0.175 0.049 0.014 0.297

2279/2280 11 16 0.500 0.871 0.830 0.460 0.296 0.121 0.039 0.338

2281/2282 15 16 1.000 0.919 0.883 0.350 0.212 0.067 0.020 0.310

2289/2290 16 16 1.000 0.923 0.886 0.343 0.207 0.065 0.019 0.308

2297/2298 15 16 1.000 0.903 0.866 0.386 0.238 0.080 0.025 0.319

2323/2324 11 16 0.938 0.895 0.853 0.423 0.267 0.107 0.032 0.324

2361/2362 8 16 0.438 0.766 0.705 0.648 0.471 0.278 0.104 0.405

2379/2380 15 16 0.750 0.923 0.886 0.346 0.209 0.067 0.020 0.308

2389/2390 15 16 0.875 0.871 0.834 0.446 0.284 0.105 0.035 0.337

2397/2398 9 16 0.813 0.869 0.822 0.484 0.316 0.144 0.044 0.340

2473/2474 14 16 0.875 0.933 0.896 0.325 0.194 0.060 0.017 0.302

2487/2488 10 16 0.688 0.879 0.837 0.451 0.289 0.119 0.037 0.333

2491/2492 14 15 0.667 0.943 0.904 0.306 0.180 0.053 0.015 0.298

2517/2518 15 15 1.000 0.920 0.879 0.362 0.221 0.074 0.022 0.311

2527/2528 12 16 0.375 0.907 0.867 0.392 0.243 0.090 0.026 0.317

2535/2536 6 15 0.267 0.766 0.695 0.671 0.497 0.316 0.113 0.408

2537/2538 14 15 0.867 0.910 0.869 0.384 0.237 0.084 0.025 0.316

2599/2600 12 16 0.563 0.865 0.823 0.472 0.307 0.129 0.042 0.341

2615/2616 14 16 1.000 0.927 0.890 0.341 0.205 0.066 0.019 0.306

2623/2624 12 15 1.000 0.908 0.867 0.391 0.242 0.088 0.026 0.318

2787/2788 10 16 0.563 0.853 0.807 0.505 0.334 0.153 0.049 0.349

2847/2848 13 16 1.000 0.919 0.881 0.361 0.219 0.075 0.022 0.310

2857/2858 10 15 1.000 0.839 0.787 0.538 0.364 0.180 0.059 0.359

2861/2862 19 16 1.000 0.925 0.890 0.330 0.199 0.057 0.017 0.306

2869/2870 18 15 0.867 0.961 0.924 0.251 0.144 0.035 0.009 0.288

2895/2896 20 16 0.938 0.962 0.928 0.242 0.138 0.032 0.009 0.286

2895/2896 23 16 1.000 0.970 0.936 0.215 0.121 0.024 0.007 0.282

2955/2956 16 16 0.500 0.948 0.912 0.285 0.166 0.045 0.013 0.294

2973/2974 24 16 1.000 0.978 0.945 0.191 0.106 0.019 0.005 0.278

2979/2980 16 13 0.538 0.960 0.918 0.270 0.156 0.041 0.011 0.291

2987/2988 14 15 0.467 0.885 0.842 0.437 0.279 0.111 0.035 0.331

3017/3018 8 16 0.500 0.835 0.787 0.539 0.362 0.176 0.058 0.360

3027/3028 14 16 0.875 0.931 0.894 0.330 0.197 0.062 0.018 0.303

3029/3030 15 16 0.688 0.889 0.852 0.412 0.258 0.090 0.029 0.327

3051/3052 15 16 0.563 0.891 0.851 0.418 0.264 0.099 0.031 0.326

3091/3092 13 15 0.867 0.908 0.867 0.390 0.242 0.088 0.026 0.318

3119/3120 15 16 0.813 0.915 0.877 0.366 0.224 0.076 0.022 0.312

3139/3140 18 16 1.000 0.952 0.917 0.271 0.157 0.040 0.011 0.292

3163/3164 19 16 0.750 0.960 0.925 0.248 0.142 0.034 0.009 0.287

3181/3182 9 16 1.000 0.907 0.866 0.400 0.248 0.095 0.027 0.317

3205/3206 16 16 1.000 0.931 0.895 0.322 0.192 0.057 0.016 0.303

3245/3246 19 15 0.800 0.966 0.929 0.237 0.135 0.031 0.008 0.285

3253/3254 16 16 0.563 0.897 0.859 0.399 0.249 0.088 0.027 0.322

3319/3320 21 16 1.000 0.964 0.930 0.234 0.133 0.029 0.008 0.285

3331/3332 15 16 0.938 0.940 0.903 0.306 0.181 0.052 0.015 0.299

3343/3344 15 16 0.813 0.919 0.881 0.358 0.218 0.073 0.021 0.310

3351/3352 14 16 0.813 0.933 0.897 0.325 0.193 0.060 0.017 0.302

3353/3354 12 15 0.867 0.885 0.843 0.435 0.275 0.106 0.033 0.331

3355/3356 9 16 1.000 0.835 0.787 0.537 0.361 0.175 0.058 0.360

5211-B07 7 16 0.563 0.849 0.799 0.525 0.351 0.173 0.055 0.353

535/536 8 15 0.267 0.630 0.586 0.772 0.585 0.374 0.176 0.490

541/542 20 16 0.875 0.968 0.934 0.225 0.127 0.028 0.007 0.283

615/616 11 16 0.375 0.889 0.848 0.430 0.273 0.108 0.033 0.328

631/632 11 16 0.250 0.817 0.769 0.558 0.380 0.185 0.065 0.371

687/688 16 15 1.000 0.954 0.917 0.273 0.158 0.041 0.011 0.292

NFSG-009 13 16 0.875 0.921 0.883 0.355 0.215 0.072 0.021 0.309

NFSG-035 13 15 0.800 0.936 0.897 0.325 0.194 0.060 0.017 0.302

NFSG-036 17 16 0.938 0.946 0.910 0.289 0.169 0.046 0.013 0.295

NFSG-065 9 16 0.750 0.798 0.745 0.600 0.420 0.228 0.080 0.383

NFSG-112 3 16 0.688 0.522 0.450 0.872 0.735 0.590 0.300 0.572

NFSG-125 5 16 0.313 0.472 0.429 0.887 0.733 0.566 0.322 0.602

NFSG-133 11 14 0.714 0.899 0.855 0.417 0.262 0.101 0.030 0.324

NFSG-134 10 16 0.375 0.802 0.748 0.592 0.414 0.223 0.079 0.381

NFSG-137 9 16 0.313 0.859 0.811 0.501 0.331 0.155 0.049 0.346

NFSG-139 7 16 0.500 0.599 0.557 0.799 0.616 0.409 0.200 0.510

NFSG-200 15 16 0.875 0.921 0.884 0.350 0.212 0.068 0.020 0.309

NFSG-202 4 15 0.600 0.625 0.529 0.810 0.673 0.518 0.232 0.506

NFSG-219 19 16 0.938 0.962 0.928 0.243 0.138 0.032 0.009 0.286

NFSG-246 6 16 0.750 0.714 0.649 0.716 0.540 0.350 0.138 0.439

PV1115/1116 16 16 0.563 0.944 0.908 0.295 0.173 0.048 0.014 0.296

PV1143/1144 20 16 1.000 0.960 0.926 0.246 0.141 0.033 0.009 0.287

PV1197/1198 17 16 1.000 0.948 0.912 0.284 0.166 0.045 0.013 0.294

PV1729/1730 18 16 1.000 0.954 0.919 0.266 0.153 0.039 0.011 0.291

PVCA-145/146 2 16 0.063 0.063 0.059 0.998 0.971 0.944 0.884 0.941

PVCA-17/18 18 16 1.000 0.948 0.912 0.282 0.164 0.044 0.012 0.294

PVCA-173/174 19 16 1.000 0.962 0.928 0.242 0.138 0.032 0.009 0.286

PVCA-19/20 20 16 0.875 0.954 0.919 0.261 0.151 0.036 0.010 0.291

PVCA-285/286 9 16 0.938 0.837 0.787 0.542 0.366 0.183 0.060 0.360

PVCA-317/318 12 16 0.813 0.891 0.850 0.424 0.268 0.105 0.032 0.326

PVCA-349/350 22 16 1.000 0.970 0.936 0.217 0.122 0.025 0.007 0.282

PVCA-415/416 20 16 0.938 0.964 0.930 0.236 0.134 0.030 0.008 0.285

PVCA-7/8 22 16 1.000 0.978 0.945 0.193 0.107 0.020 0.005 0.278

PVCA-815/816 12 16 0.500 0.883 0.842 0.440 0.280 0.112 0.035 0.331

PVCA-893/894 9 16 0.563 0.829 0.780 0.546 0.370 0.182 0.061 0.364

PVCA-979/980 8 16 0.938 0.726 0.678 0.679 0.491 0.281 0.113 0.427

sww-2662 10 15 0.733 0.823 0.774 0.555 0.377 0.185 0.064 0.368

sww-1394 8 16 0.500 0.698 0.651 0.709 0.521 0.309 0.130 0.445

sww-1615 17 16 0.938 0.909 0.873 0.368 0.225 0.071 0.022 0.315

sww-1622 6 15 0.867 0.828 0.770 0.576 0.398 0.218 0.070 0.368

sww-1643 12 14 0.500 0.923 0.881 0.361 0.219 0.074 0.021 0.310

sww-1667 8 15 0.200 0.759 0.710 0.642 0.454 0.246 0.094 0.407

sww-1749 9 16 0.688 0.855 0.808 0.506 0.335 0.157 0.050 0.348

sww-1754 8 16 0.438 0.806 0.748 0.600 0.423 0.239 0.081 0.380

sww-1761 15 16 0.938 0.938 0.901 0.313 0.185 0.055 0.016 0.300

sww-1795 10 15 1.000 0.862 0.814 0.493 0.325 0.148 0.047 0.345

sww-1813 10 15 0.600 0.878 0.833 0.459 0.295 0.125 0.039 0.335

sww-1889 4 16 0.375 0.490 0.424 0.884 0.749 0.603 0.326 0.594

sww-1969 14 14 0.929 0.926 0.884 0.351 0.212 0.069 0.020 0.309

sww-2034 8 15 1.000 0.782 0.721 0.630 0.452 0.260 0.095 0.396

sww-2070 6 16 0.875 0.738 0.670 0.691 0.517 0.329 0.126 0.424

sww-2167 10 16 1.000 0.863 0.817 0.489 0.321 0.145 0.046 0.343

sww-2235 3 13 0.154 0.151 0.140 0.989 0.926 0.863 0.736 0.862

sww-223 6 16 1.000 0.706 0.647 0.719 0.537 0.341 0.136 0.442

sww-2320 10 16 0.688 0.760 0.716 0.632 0.444 0.234 0.090 0.404

sww-2368 8 16 0.500 0.825 0.770 0.568 0.392 0.209 0.069 0.368

sww-2376 9 16 0.750 0.710 0.655 0.703 0.521 0.318 0.130 0.439

sww-2377 3 16 0.125 0.123 0.116 0.993 0.940 0.886 0.779 0.885

sww-2387 3 15 0.133 0.660 0.565 0.797 0.651 0.501 0.204 0.482

sww-2503 9 16 0.500 0.788 0.732 0.616 0.437 0.245 0.087 0.390

sww-2527 11 16 0.750 0.883 0.840 0.448 0.287 0.121 0.037 0.331

sww-2532 8 14 0.429 0.743 0.685 0.672 0.489 0.287 0.112 0.419

sww-2545 8 16 0.500 0.567 0.531 0.818 0.635 0.426 0.222 0.531

sww-2578 4 15 0.800 0.595 0.494 0.829 0.703 0.556 0.261 0.528

sww-333 7 16 0.750 0.742 0.674 0.686 0.513 0.326 0.124 0.422

sww-387 5 16 0.375 0.468 0.422 0.890 0.741 0.580 0.331 0.606

sww-389 7 12 0.000 0.797 0.739 0.607 0.423 0.224 0.080 0.388

sww-463 7 16 0.438 0.815 0.762 0.580 0.401 0.213 0.072 0.373

sww-532 10 15 0.867 0.823 0.770 0.563 0.387 0.199 0.067 0.369

sww-556 13 16 0.938 0.899 0.861 0.398 0.247 0.087 0.027 0.321

sww-573 11 16 0.688 0.810 0.767 0.561 0.380 0.180 0.064 0.374

SWW-611 6 16 0.313 0.655 0.602 0.760 0.578 0.378 0.166 0.474

Number of individuals: 16

Number of loci: 166

Mean number of alleles per locus: 13.27

Mean proportion of individuals typed: 0.9782

Mean expected heterozygosity: 0.8531

Mean polymorphic information content (PIC): 0.8103

Combined non-exclusion probability (first parent): 1.17E-0066

Combined non-exclusion probability (second parent): 8.58E-0099

Combined non-exclusion probability (parent pair): 1.75E-0170

Combined non-exclusion probability (identity): 5.16E-0252

Combined non-exclusion probability (sib identity): 8.90E-0078

**Note:** The entries in the table of summary statistics for the loci used in the allele frequency analysis are as follows:

Locus: Name of locus read from the genotype file, or a locus number if no locus names were read.

k: Number of alleles at the locus.

N: Number of individuals typed at the locus.

HObs: Observed heterozygosity.

HExp: Expected heterozygosity.

PIC: Polymorphic information content.

NE-1P: Average non-exclusion probability for one candidate parent.

NE-2P: Average non-exclusion probability for one candidate parent given the genotype of a known parent of the opposite sex.

NE-PP: Average non-exclusion probability for a candidate parent pair.

NE-I: Average non-exclusion probability for identity of two unrelated individuals.

NE-SI: Average non-exclusion probability for identity of two siblings.

**** Files ****

Input

Genotype data file: Genotype.csv

Output

Summary text file: Allele frequency new.txt

Allele frequency file: Allele frequency new.alf

**** Loci ****

1 1105/1106

2 1115/1116

3 1149/1150

4 1209/1210

5 1243/1244

6 1251/1252

7 1291/1292

8 1301/1302

9 1311/1312

10 1353/1354

11 1357/1358

12 1387/1388

13 1499/1500

14 1513/1514

15 1549/1550

16 1555/1556

17 1559/1560

18 1599/1600

19 1649/1650

20 1663/1664

21 1669/1670

22 1719/1720

23 1723/1724

24 1733/1734

25 1735/1736

26 1773/1774

27 179/180

28 1813/1814

29 1843/1844

30 1941/1942

31 1947/1948

32 1957/1958

33 1963/1964

34 1983/1984

35 2005/2006

36 2081/2082

37 2107/2108

38 2119/2120

39 2139/2140

40 2147/2148

41 2153/2154

42 2187/2188

43 2199/2200

44 2207/2208

45 2209/2210

46 2269/2270

47 2279/2280

48 2281/2282

49 2289/2290

50 2297/2298

51 2323/2324

52 2361/2362

53 2379/2380

54 2389/2390

55 2397/2398

56 2473/2474

57 2487/2488

58 2491/2492

59 2517/2518

60 2527/2528

61 2535/2536

62 2537/2538

63 2599/2600

64 2615/2616

65 2623/2624

66 2787/2788

67 2847/2848

68 2857/2858

69 2861/2862

70 2869/2870

71 2895/2896

72 2895/2896

73 2955/2956

74 2973/2974

75 2979/2980

76 2987/2988

77 3017/3018

78 3027/3028

79 3029/3030

80 3051/3052

81 3091/3092

82 3119/3120

83 3139/3140

84 3163/3164

85 3181/3182

86 3205/3206

87 3245/3246

88 3253/3254

89 3319/3320

90 3331/3332

91 3343/3344

92 3351/3352

93 3353/3354

94 3355/3356

95 5211-B07

96 535/536

97 541/542

98 615/616

99 631/632

100 687/688

101 NFSG-009

102 NFSG-035

103 NFSG-036

104 NFSG-065

105 NFSG-112

106 NFSG-125

107 NFSG-133

108 NFSG-134

109 NFSG-137

110 NFSG-139

111 NFSG-200

112 NFSG-202

113 NFSG-219

114 NFSG-246

115 PV1115/1116

116 PV1143/1144

117 PV1197/1198

118 PV1729/1730

119 PVCA-145/146

120 PVCA-17/18

121 PVCA-173/174

122 PVCA-19/20

123 PVCA-285/286

124 PVCA-317/318

125 PVCA-349/350

126 PVCA-415/416

127 PVCA-7/8

128 PVCA-815/816

129 PVCA-893/894

130 PVCA-979/980

131 sww-2662

132 sww-1394

133 sww-1615

134 sww-1622

135 sww-1643

136 sww-1667

137 sww-1749

138 sww-1754

139 sww-1761

140 sww-1795

141 sww-1813

142 sww-1889

143 sww-1969

144 sww-2034

145 sww-2070

146 sww-2167

147 sww-2235

148 sww-223

149 sww-2320

150 sww-2368

151 sww-2376

152 sww-2377

153 sww-2387

154 sww-2503

155 sww-2527

156 sww-2532

157 sww-2545

158 sww-2578

159 sww-333

160 sww-387

161 sww-389

162 sww-463

163 sww-532

164 sww-556

165 sww-573

166 SWW-611

**** Locus 1105/1106 ****

Allele Count Heterozygotes Homozygotes Frequency

148 2 2 0 0.0667

153 1 1 0 0.0333

157 2 2 0 0.0667

162 1 1 0 0.0333

164 1 1 0 0.0333

166 1 1 0 0.0333

167 2 2 0 0.0667

180 1 1 0 0.0333

185 1 1 0 0.0333

188 1 1 0 0.0333

189 1 1 0 0.0333

190 1 1 0 0.0333

191 2 2 0 0.0667

192 3 3 0 0.1000

193 1 1 0 0.0333

194 2 2 0 0.0667

195 1 1 0 0.0333

197 1 1 0 0.0333

198 2 2 0 0.0667

199 1 1 0 0.0333

202 1 1 0 0.0333

209 1 1 0 0.0333

Number of individuals typed: 15

Heterozygotes: 15

Homozygotes: 0

Number of alleles: 22

Observed heterozygosity: 1.0000

Expected heterozygosity: 0.9793

Polymorphic information content (PIC): 0.9441

Average non-exclusion probability (first parent): 0.1950

Average non-exclusion probability (second parent): 0.1081

Average non-exclusion probability (parent pair): 0.0204

Average non-exclusion probability (identity): 0.0055

Average non-exclusion probability (sib identity): 0.2780

**** Locus 1115/1116 ****

Allele Count Heterozygotes Homozygotes Frequency

92 2 0 1 0.0667

139 1 1 0 0.0333

140 1 1 0 0.0333

142 1 1 0 0.0333

144 4 4 0 0.1333

145 1 1 0 0.0333

146 1 1 0 0.0333

147 3 3 0 0.1000

149 1 1 0 0.0333

150 3 3 0 0.1000

151 2 2 0 0.0667

153 1 1 0 0.0333

154 2 2 0 0.0667

155 2 2 0 0.0667

156 1 1 0 0.0333

158 1 1 0 0.0333

159 1 1 0 0.0333

161 1 1 0 0.0333

163 1 1 0 0.0333

Number of individuals typed: 15

Heterozygotes: 14

Homozygotes: 1

Number of alleles: 19

Observed heterozygosity: 0.9333

Expected heterozygosity: 0.9632

Polymorphic information content (PIC): 0.9270

Average non-exclusion probability (first parent): 0.2439

Average non-exclusion probability (second parent): 0.1390

Average non-exclusion probability (parent pair): 0.0324

Average non-exclusion probability (identity): 0.0089

Average non-exclusion probability (sib identity): 0.2867

**** Locus 1149/1150 ****

Allele Count Heterozygotes Homozygotes Frequency

195 1 1 0 0.0313

202 1 1 0 0.0313

205 2 2 0 0.0625

206 2 2 0 0.0625

209 5 5 0 0.1563

210 1 1 0 0.0313

211 4 2 1 0.1250

212 6 2 2 0.1875

213 2 2 0 0.0625

214 2 2 0 0.0625

215 1 1 0 0.0313

217 1 1 0 0.0313

221 1 1 0 0.0313

223 2 2 0 0.0625

224 1 1 0 0.0313

Number of individuals typed: 16

Heterozygotes: 13

Homozygotes: 3

Number of alleles: 15

Observed heterozygosity: 0.8125

Expected heterozygosity: 0.9274

Polymorphic information content (PIC): 0.8903

Average non-exclusion probability (first parent): 0.3369

Average non-exclusion probability (second parent): 0.2025

Average non-exclusion probability (parent pair): 0.0639

Average non-exclusion probability (identity): 0.0185

Average non-exclusion probability (sib identity): 0.3054

**** Locus 1209/1210 ****

Allele Count Heterozygotes Homozygotes Frequency

244 1 1 0 0.0357

245 1 1 0 0.0357

248 1 1 0 0.0357

252 1 1 0 0.0357

254 2 2 0 0.0714

257 1 1 0 0.0357

258 1 1 0 0.0357

259 1 1 0 0.0357

261 1 1 0 0.0357

264 2 2 0 0.0714

265 1 1 0 0.0357

267 2 2 0 0.0714

269 1 1 0 0.0357

270 1 1 0 0.0357

271 1 1 0 0.0357

274 1 1 0 0.0357

275 2 2 0 0.0714

276 1 1 0 0.0357

279 2 2 0 0.0714

282 1 1 0 0.0357

292 1 1 0 0.0357

316 1 1 0 0.0357

320 1 1 0 0.0357

Number of individuals typed: 14

Heterozygotes: 14

Homozygotes: 0

Number of alleles: 23

Observed heterozygosity: 1.0000

Expected heterozygosity: 0.9868

Polymorphic information content (PIC): 0.9493

Average non-exclusion probability (first parent): 0.1791

Average non-exclusion probability (second parent): 0.0983

Average non-exclusion probability (parent pair): 0.0172

Average non-exclusion probability (identity): 0.0045

Average non-exclusion probability (sib identity): 0.2754

**** Locus 1243/1244 ****

Allele Count Heterozygotes Homozygotes Frequency

280 1 1 0 0.0313

283 1 1 0 0.0313

285 1 1 0 0.0313

288 1 1 0 0.0313

292 1 1 0 0.0313

295 1 1 0 0.0313

296 1 1 0 0.0313

297 1 1 0 0.0313

298 1 1 0 0.0313

299 4 4 0 0.1250

300 1 1 0 0.0313

301 1 1 0 0.0313

302 1 1 0 0.0313

303 1 1 0 0.0313

305 2 2 0 0.0625

307 1 1 0 0.0313

308 1 1 0 0.0313

309 1 1 0 0.0313

310 2 2 0 0.0625

311 3 3 0 0.0938

312 1 1 0 0.0313

313 1 1 0 0.0313

314 1 1 0 0.0313

315 1 1 0 0.0313

317 1 1 0 0.0313

Number of individuals typed: 16

Heterozygotes: 16

Homozygotes: 0

Number of alleles: 25

Observed heterozygosity: 1.0000

Expected heterozygosity: 0.9778

Polymorphic information content (PIC): 0.9449

Average non-exclusion probability (first parent): 0.1909

Average non-exclusion probability (second parent): 0.1059

Average non-exclusion probability (parent pair): 0.0193

Average non-exclusion probability (identity): 0.0052

Average non-exclusion probability (sib identity): 0.2777

**** Locus 1251/1252 ****

Allele Count Heterozygotes Homozygotes Frequency

247 1 1 0 0.0313

257 3 3 0 0.0938

267 2 2 0 0.0625

268 2 2 0 0.0625

277 1 1 0 0.0313

279 1 1 0 0.0313

280 1 1 0 0.0313

282 2 2 0 0.0625

283 2 2 0 0.0625

284 5 5 0 0.1563

287 1 1 0 0.0313

288 2 2 0 0.0625

289 1 1 0 0.0313

290 1 1 0 0.0313

294 1 1 0 0.0313

298 1 1 0 0.0313

300 1 1 0 0.0313

303 1 1 0 0.0313

304 1 1 0 0.0313

308 1 1 0 0.0313

318 1 1 0 0.0313

Number of individuals typed: 16

Heterozygotes: 16

Homozygotes: 0

Number of alleles: 21

Observed heterozygosity: 1.0000

Expected heterozygosity: 0.9637

Polymorphic information content (PIC): 0.9299

Average non-exclusion probability (first parent): 0.2339

Average non-exclusion probability (second parent): 0.1329

Average non-exclusion probability (parent pair): 0.0293

Average non-exclusion probability (identity): 0.0081

Average non-exclusion probability (sib identity): 0.2852

**** Locus 1291/1292 ****

Allele Count Heterozygotes Homozygotes Frequency

170 1 1 0 0.0333

171 2 2 0 0.0667

173 5 5 0 0.1667

174 9 7 1 0.3000

175 6 2 2 0.2000

176 6 6 0 0.2000

178 1 1 0 0.0333

Number of individuals typed: 15

Heterozygotes: 12

Homozygotes: 3

Number of alleles: 7

Observed heterozygosity: 0.8000

Expected heterozygosity: 0.8230

Polymorphic information content (PIC): 0.7659

Average non-exclusion probability (first parent): 0.5785

Average non-exclusion probability (second parent): 0.4005

Average non-exclusion probability (parent pair): 0.2183

Average non-exclusion probability (identity): 0.0715

Average non-exclusion probability (sib identity): 0.3701

**** Locus 1301/1302 ****

Allele Count Heterozygotes Homozygotes Frequency

163 1 1 0 0.0313

168 1 1 0 0.0313

169 2 2 0 0.0625

171 1 1 0 0.0313

176 3 3 0 0.0938

182 1 1 0 0.0313

186 3 3 0 0.0938

191 3 3 0 0.0938

192 1 1 0 0.0313

193 4 4 0 0.1250

194 3 3 0 0.0938

196 4 4 0 0.1250

198 1 1 0 0.0313

200 1 1 0 0.0313

217 1 1 0 0.0313

223 1 1 0 0.0313

226 1 1 0 0.0313

Number of individuals typed: 16

Heterozygotes: 16

Homozygotes: 0

Number of alleles: 17

Observed heterozygosity: 1.0000

Expected heterozygosity: 0.9496

Polymorphic information content (PIC): 0.9143

Average non-exclusion probability (first parent): 0.2789

Average non-exclusion probability (second parent): 0.1620

Average non-exclusion probability (parent pair): 0.0433

Average non-exclusion probability (identity): 0.0120

Average non-exclusion probability (sib identity): 0.2930

**** Locus 1311/1312 ****

Allele Count Heterozygotes Homozygotes Frequency

136 1 1 0 0.0333

137 1 1 0 0.0333

138 1 1 0 0.0333

140 1 1 0 0.0333

141 4 4 0 0.1333

147 2 2 0 0.0667

148 1 1 0 0.0333

150 3 3 0 0.1000

152 1 1 0 0.0333

156 2 2 0 0.0667

157 1 1 0 0.0333

159 1 1 0 0.0333

160 1 1 0 0.0333

163 1 1 0 0.0333

172 1 1 0 0.0333

174 1 1 0 0.0333

175 1 1 0 0.0333

176 2 0 1 0.0667

177 2 2 0 0.0667

178 1 1 0 0.0333

181 1 1 0 0.0333

Number of individuals typed: 15

Heterozygotes: 14

Homozygotes: 1

Number of alleles: 21

Observed heterozygosity: 0.9333

Expected heterozygosity: 0.9701

Polymorphic information content (PIC): 0.9344

Average non-exclusion probability (first parent): 0.2222

Average non-exclusion probability (second parent): 0.1253

Average non-exclusion probability (parent pair): 0.0266

Average non-exclusion probability (identity): 0.0072

Average non-exclusion probability (sib identity): 0.2829

**** Locus 1353/1354 ****

Allele Count Heterozygotes Homozygotes Frequency

139 10 10 0 0.3125

149 16 16 0 0.5000

150 6 6 0 0.1875

Number of individuals typed: 16

Heterozygotes: 16

Homozygotes: 0

Number of alleles: 3

Observed heterozygosity: 1.0000

Expected heterozygosity: 0.6371

Polymorphic information content (PIC): 0.5439

Average non-exclusion probability (first parent): 0.8095

Average non-exclusion probability (second parent): 0.6673

Average non-exclusion probability (parent pair): 0.5179

Average non-exclusion probability (identity): 0.2198

Average non-exclusion probability (sib identity): 0.4964

**** Locus 1357/1358 ****

Allele Count Heterozygotes Homozygotes Frequency

229 1 1 0 0.0313

269 2 2 0 0.0625

270 1 1 0 0.0313

273 4 4 0 0.1250

274 2 2 0 0.0625

275 1 1 0 0.0313

278 1 1 0 0.0313

284 1 1 0 0.0313

286 1 1 0 0.0313

287 1 1 0 0.0313

291 1 1 0 0.0313

294 2 2 0 0.0625

296 2 2 0 0.0625

300 1 1 0 0.0313

303 3 3 0 0.0938

304 2 2 0 0.0625

306 1 1 0 0.0313

307 1 1 0 0.0313

311 1 1 0 0.0313

322 1 1 0 0.0313

326 1 1 0 0.0313

337 1 1 0 0.0313

Number of individuals typed: 16

Heterozygotes: 16

Homozygotes: 0

Number of alleles: 22

Observed heterozygosity: 1.0000

Expected heterozygosity: 0.9718

Polymorphic information content (PIC): 0.9384

Average non-exclusion probability (first parent): 0.2109

Average non-exclusion probability (second parent): 0.1181

Average non-exclusion probability (parent pair): 0.0239

Average non-exclusion probability (identity): 0.0065

Average non-exclusion probability (sib identity): 0.2809

**** Locus 1387/1388 ****

Allele Count Heterozygotes Homozygotes Frequency

214 1 1 0 0.0313

220 1 1 0 0.0313

221 1 1 0 0.0313

224 2 2 0 0.0625

232 1 1 0 0.0313

234 1 1 0 0.0313

235 11 7 2 0.3438

236 1 1 0 0.0313

237 2 2 0 0.0625

242 1 1 0 0.0313

244 5 5 0 0.1563

245 1 1 0 0.0313

247 1 1 0 0.0313

255 1 1 0 0.0313

257 1 1 0 0.0313

258 1 1 0 0.0313

Number of individuals typed: 16

Heterozygotes: 14

Homozygotes: 2

Number of alleles: 16

Observed heterozygosity: 0.8750

Expected heterozygosity: 0.8649

Polymorphic information content (PIC): 0.8262

Average non-exclusion probability (first parent): 0.4585

Average non-exclusion probability (second parent): 0.2949

Average non-exclusion probability (parent pair): 0.1136

Average non-exclusion probability (identity): 0.0380

Average non-exclusion probability (sib identity): 0.3405

**** Locus 1499/1500 ****

Allele Count Heterozygotes Homozygotes Frequency

163 3 3 0 0.0938

164 1 1 0 0.0313

165 1 1 0 0.0313

166 1 1 0 0.0313

167 3 3 0 0.0938

169 2 2 0 0.0625

170 1 1 0 0.0313

171 2 2 0 0.0625

176 2 2 0 0.0625

177 1 1 0 0.0313

178 3 3 0 0.0938

182 1 1 0 0.0313

184 1 1 0 0.0313

190 3 3 0 0.0938

194 3 1 1 0.0938

195 1 1 0 0.0313

198 1 1 0 0.0313

200 2 2 0 0.0625

Number of individuals typed: 16

Heterozygotes: 15

Homozygotes: 1

Number of alleles: 18

Observed heterozygosity: 0.9375

Expected heterozygosity: 0.9617

Polymorphic information content (PIC): 0.9274

Average non-exclusion probability (first parent): 0.2440

Average non-exclusion probability (second parent): 0.1388

Average non-exclusion probability (parent pair): 0.0327

Average non-exclusion probability (identity): 0.0089

Average non-exclusion probability (sib identity): 0.2864

**** Locus 1513/1514 ****

Allele Count Heterozygotes Homozygotes Frequency

236 2 2 0 0.0667

240 1 1 0 0.0333

243 1 1 0 0.0333

244 1 1 0 0.0333

247 1 1 0 0.0333

248 3 3 0 0.1000

249 7 5 1 0.2333

251 2 2 0 0.0667

252 1 1 0 0.0333

253 3 3 0 0.1000

254 2 0 1 0.0667

255 1 1 0 0.0333

256 1 1 0 0.0333

268 1 1 0 0.0333

272 1 1 0 0.0333

274 2 2 0 0.0667

Number of individuals typed: 15

Heterozygotes: 13

Homozygotes: 2

Number of alleles: 16

Observed heterozygosity: 0.8667

Expected heterozygosity: 0.9287

Polymorphic information content (PIC): 0.8906

Average non-exclusion probability (first parent): 0.3329

Average non-exclusion probability (second parent): 0.1998

Average non-exclusion probability (parent pair): 0.0601

Average non-exclusion probability (identity): 0.0176

Average non-exclusion probability (sib identity): 0.3055

**** Locus 1549/1550 ****

Allele Count Heterozygotes Homozygotes Frequency

278 1 1 0 0.0313

281 5 5 0 0.1563

283 4 4 0 0.1250

284 3 3 0 0.0938

285 8 8 0 0.2500

321 1 1 0 0.0313

323 1 1 0 0.0313

326 3 3 0 0.0938

328 2 2 0 0.0625

329 1 1 0 0.0313

331 3 3 0 0.0938

Number of individuals typed: 16

Heterozygotes: 16

Homozygotes: 0

Number of alleles: 11

Observed heterozygosity: 1.0000

Expected heterozygosity: 0.8911

Polymorphic information content (PIC): 0.8496

Average non-exclusion probability (first parent): 0.4276

Average non-exclusion probability (second parent): 0.2700

Average non-exclusion probability (parent pair): 0.1072

Average non-exclusion probability (identity): 0.0324

Average non-exclusion probability (sib identity): 0.3265

**** Locus 1555/1556 ****

Allele Count Heterozygotes Homozygotes Frequency

236 1 1 0 0.0313

242 3 3 0 0.0938

243 4 4 0 0.1250

244 2 2 0 0.0625

246 3 3 0 0.0938

248 1 1 0 0.0313

250 1 1 0 0.0313

252 1 1 0 0.0313

253 4 4 0 0.1250

254 1 1 0 0.0313

255 2 2 0 0.0625

256 1 1 0 0.0313

257 2 2 0 0.0625

258 1 1 0 0.0313

260 1 1 0 0.0313

261 1 1 0 0.0313

262 1 1 0 0.0313

265 1 1 0 0.0313

267 1 1 0 0.0313

Number of individuals typed: 16

Heterozygotes: 16

Homozygotes: 0

Number of alleles: 19

Observed heterozygosity: 1.0000

Expected heterozygosity: 0.9577

Polymorphic information content (PIC): 0.9232

Average non-exclusion probability (first parent): 0.2541

Average non-exclusion probability (second parent): 0.1457

Average non-exclusion probability (parent pair): 0.0354

Average non-exclusion probability (identity): 0.0097

Average non-exclusion probability (sib identity): 0.2886

**** Locus 1559/1560 ****

Allele Count Heterozygotes Homozygotes Frequency

284 1 1 0 0.0313

285 1 1 0 0.0313

287 2 2 0 0.0625

288 1 1 0 0.0313

291 1 1 0 0.0313

294 2 2 0 0.0625

295 1 1 0 0.0313

297 4 4 0 0.1250

298 1 1 0 0.0313

300 6 6 0 0.1875

302 5 5 0 0.1563

308 1 1 0 0.0313

310 1 1 0 0.0313

312 1 1 0 0.0313

329 4 4 0 0.1250

Number of individuals typed: 16

Heterozygotes: 16

Homozygotes: 0

Number of alleles: 15

Observed heterozygosity: 1.0000

Expected heterozygosity: 0.9214

Polymorphic information content (PIC): 0.8834

Average non-exclusion probability (first parent): 0.3534

Average non-exclusion probability (second parent): 0.2144

Average non-exclusion probability (parent pair): 0.0712

Average non-exclusion probability (identity): 0.0207

Average non-exclusion probability (sib identity): 0.3089

**** Locus 1599/1600 ****

Allele Count Heterozygotes Homozygotes Frequency

246 14 12 1 0.4375

247 1 1 0 0.0313

253 2 2 0 0.0625

254 1 1 0 0.0313

255 1 1 0 0.0313

265 4 4 0 0.1250

266 2 2 0 0.0625

268 1 1 0 0.0313

269 1 1 0 0.0313

271 1 1 0 0.0313

272 1 1 0 0.0313

280 1 1 0 0.0313

283 1 1 0 0.0313

288 1 1 0 0.0313

Number of individuals typed: 16

Heterozygotes: 15

Homozygotes: 1

Number of alleles: 14

Observed heterozygosity: 0.9375

Expected heterozygosity: 0.8004

Polymorphic information content (PIC): 0.7619

Average non-exclusion probability (first parent): 0.5624

Average non-exclusion probability (second parent): 0.3797

Average non-exclusion probability (parent pair): 0.1708

Average non-exclusion probability (identity): 0.0640

Average non-exclusion probability (sib identity): 0.3783

**** Locus 1649/1650 ****

Allele Count Heterozygotes Homozygotes Frequency

164 2 2 0 0.0625

165 3 3 0 0.0938

166 1 1 0 0.0313

168 3 3 0 0.0938

169 2 2 0 0.0625

170 1 1 0 0.0313

173 1 1 0 0.0313

175 2 2 0 0.0625

179 1 1 0 0.0313

180 2 2 0 0.0625

183 2 2 0 0.0625

184 1 1 0 0.0313

185 2 2 0 0.0625

186 3 3 0 0.0938

187 1 1 0 0.0313

198 1 1 0 0.0313

219 1 1 0 0.0313

220 1 1 0 0.0313

221 1 1 0 0.0313

226 1 1 0 0.0313

Number of individuals typed: 16

Heterozygotes: 16

Homozygotes: 0

Number of alleles: 20

Observed heterozygosity: 1.0000

Expected heterozygosity: 0.9698

Polymorphic information content (PIC): 0.9361

Average non-exclusion probability (first parent): 0.2188

Average non-exclusion probability (second parent): 0.1228

Average non-exclusion probability (parent pair): 0.0260

Average non-exclusion probability (identity): 0.0070

Average non-exclusion probability (sib identity): 0.2820

**** Locus 1663/1664 ****

Allele Count Heterozygotes Homozygotes Frequency

203 8 2 3 0.2500

204 2 2 0 0.0625

205 2 0 1 0.0625

206 1 1 0 0.0313

208 1 1 0 0.0313

210 3 3 0 0.0938

211 1 1 0 0.0313

213 1 1 0 0.0313

216 1 1 0 0.0313

217 2 2 0 0.0625

221 1 1 0 0.0313

222 2 2 0 0.0625

224 1 1 0 0.0313

227 2 2 0 0.0625

228 1 1 0 0.0313

229 2 2 0 0.0625

230 1 1 0 0.0313

Number of individuals typed: 16

Heterozygotes: 12

Homozygotes: 4

Number of alleles: 17

Observed heterozygosity: 0.7500

Expected heterozygosity: 0.9254

Polymorphic information content (PIC): 0.8899

Average non-exclusion probability (first parent): 0.3321

Average non-exclusion probability (second parent): 0.1994

Average non-exclusion probability (parent pair): 0.0584

Average non-exclusion probability (identity): 0.0173

Average non-exclusion probability (sib identity): 0.3061

**** Locus 1669/1670 ****

Allele Count Heterozygotes Homozygotes Frequency

202 4 4 0 0.1250

204 28 4 12 0.8750

Number of individuals typed: 16

Heterozygotes: 4

Homozygotes: 12

Number of alleles: 2

Observed heterozygosity: 0.2500

Expected heterozygosity: 0.2258

Polymorphic information content (PIC): 0.1948

Average non-exclusion probability (first parent): 0.9761

Average non-exclusion probability (second parent): 0.9026

Average non-exclusion probability (parent pair): 0.8332

Average non-exclusion probability (identity): 0.6343

Average non-exclusion probability (sib identity): 0.7992

**** Locus 1719/1720 ****

Allele Count Heterozygotes Homozygotes Frequency

165 2 2 0 0.0625

168 2 2 0 0.0625

172 1 1 0 0.0313

173 2 2 0 0.0625

176 1 1 0 0.0313

177 2 2 0 0.0625

178 2 2 0 0.0625

179 1 1 0 0.0313

180 2 2 0 0.0625

181 1 1 0 0.0313

182 2 2 0 0.0625

183 1 1 0 0.0313

184 1 1 0 0.0313

189 1 1 0 0.0313

194 2 2 0 0.0625

195 2 0 1 0.0625

196 2 2 0 0.0625

200 1 1 0 0.0313

203 1 1 0 0.0313

206 1 1 0 0.0313

209 2 2 0 0.0625

Number of individuals typed: 16

Heterozygotes: 15

Homozygotes: 1

Number of alleles: 21

Observed heterozygosity: 0.9375

Expected heterozygosity: 0.9778

Polymorphic information content (PIC): 0.9447

Average non-exclusion probability (first parent): 0.1939

Average non-exclusion probability (second parent): 0.1072

Average non-exclusion probability (parent pair): 0.0203

Average non-exclusion probability (identity): 0.0054

Average non-exclusion probability (sib identity): 0.2777

**** Locus 1723/1724 ****

Allele Count Heterozygotes Homozygotes Frequency

149 1 1 0 0.0313

150 5 5 0 0.1563

151 1 1 0 0.0313

154 1 1 0 0.0313

157 1 1 0 0.0313

160 1 1 0 0.0313

161 1 1 0 0.0313

162 1 1 0 0.0313

163 1 1 0 0.0313

164 2 2 0 0.0625

165 1 1 0 0.0313

168 3 3 0 0.0938

169 1 1 0 0.0313

170 2 2 0 0.0625

171 1 1 0 0.0313

173 1 1 0 0.0313

174 1 1 0 0.0313

175 1 1 0 0.0313

176 1 1 0 0.0313

178 2 2 0 0.0625

179 1 1 0 0.0313

186 2 2 0 0.0625

Number of individuals typed: 16

Heterozygotes: 16

Homozygotes: 0

Number of alleles: 22

Observed heterozygosity: 1.0000

Expected heterozygosity: 0.9657

Polymorphic information content (PIC): 0.9321

Average non-exclusion probability (first parent): 0.2273

Average non-exclusion probability (second parent): 0.1287

Average non-exclusion probability (parent pair): 0.0275

Average non-exclusion probability (identity): 0.0076

Average non-exclusion probability (sib identity): 0.2841

**** Locus 1733/1734 ****

Allele Count Heterozygotes Homozygotes Frequency

258 7 1 3 0.2500

263 6 0 3 0.2143

264 4 0 2 0.1429

265 2 0 1 0.0714

269 1 1 0 0.0357

280 2 2 0 0.0714

281 1 1 0 0.0357

283 2 2 0 0.0714

284 1 1 0 0.0357

291 1 1 0 0.0357

293 1 1 0 0.0357

Number of individuals typed: 14

Heterozygotes: 5

Homozygotes: 9

Number of alleles: 11

Observed heterozygosity: 0.3571

Expected heterozygosity: 0.8810

Polymorphic information content (PIC): 0.8334

Average non-exclusion probability (first parent): 0.4575

Average non-exclusion probability (second parent): 0.2947

Average non-exclusion probability (parent pair): 0.1247

Average non-exclusion probability (identity): 0.0388

Average non-exclusion probability (sib identity): 0.3350

**** Locus 1735/1736 ****

Allele Count Heterozygotes Homozygotes Frequency

222 2 2 0 0.0625

223 1 1 0 0.0313

226 3 3 0 0.0938

227 1 1 0 0.0313

228 3 3 0 0.0938

230 1 1 0 0.0313

233 3 3 0 0.0938

234 4 4 0 0.1250

235 1 1 0 0.0313

237 1 1 0 0.0313

238 1 1 0 0.0313

239 2 2 0 0.0625

240 1 1 0 0.0313

242 1 1 0 0.0313

243 4 4 0 0.1250

255 1 1 0 0.0313

256 1 1 0 0.0313

257 1 1 0 0.0313

Number of individuals typed: 16

Heterozygotes: 16

Homozygotes: 0

Number of alleles: 18

Observed heterozygosity: 1.0000

Expected heterozygosity: 0.9536

Polymorphic information content (PIC): 0.9188

Average non-exclusion probability (first parent): 0.2666

Average non-exclusion probability (second parent): 0.1538

Average non-exclusion probability (parent pair): 0.0392

Average non-exclusion probability (identity): 0.0108

Average non-exclusion probability (sib identity): 0.2908

**** Locus 1773/1774 ****

Allele Count Heterozygotes Homozygotes Frequency

185 1 1 0 0.0313

188 1 1 0 0.0313

189 1 1 0 0.0313

190 1 1 0 0.0313

192 2 2 0 0.0625

193 1 1 0 0.0313

195 1 1 0 0.0313

196 5 5 0 0.1563

198 1 1 0 0.0313

199 2 2 0 0.0625

201 1 1 0 0.0313

202 1 1 0 0.0313

204 1 1 0 0.0313

205 1 1 0 0.0313

206 1 1 0 0.0313

208 1 1 0 0.0313

209 1 1 0 0.0313

210 2 2 0 0.0625

212 3 3 0 0.0938

219 2 2 0 0.0625

227 1 1 0 0.0313

229 1 1 0 0.0313

Number of individuals typed: 16

Heterozygotes: 16

Homozygotes: 0

Number of alleles: 22

Observed heterozygosity: 1.0000

Expected heterozygosity: 0.9657

Polymorphic information content (PIC): 0.9321

Average non-exclusion probability (first parent): 0.2273

Average non-exclusion probability (second parent): 0.1287

Average non-exclusion probability (parent pair): 0.0275

Average non-exclusion probability (identity): 0.0076

Average non-exclusion probability (sib identity): 0.2841

**** Locus 179/180 ****

Allele Count Heterozygotes Homozygotes Frequency

271 1 1 0 0.0333

272 2 2 0 0.0667

273 4 4 0 0.1333

274 3 1 1 0.1000

275 1 1 0 0.0333

276 1 1 0 0.0333

277 2 2 0 0.0667

278 5 5 0 0.1667

279 4 2 1 0.1333

280 1 1 0 0.0333

283 1 1 0 0.0333

284 1 1 0 0.0333

302 1 1 0 0.0333

311 1 1 0 0.0333

326 1 1 0 0.0333

328 1 1 0 0.0333

Number of individuals typed: 15

Heterozygotes: 13

Homozygotes: 2

Number of alleles: 16

Observed heterozygosity: 0.8667

Expected heterozygosity: 0.9379

Polymorphic information content (PIC): 0.8995

Average non-exclusion probability (first parent): 0.3152

Average non-exclusion probability (second parent): 0.1871

Average non-exclusion probability (parent pair): 0.0558

Average non-exclusion probability (identity): 0.0159

Average non-exclusion probability (sib identity): 0.3006

**** Locus 1813/1814 ****

Allele Count Heterozygotes Homozygotes Frequency

236 1 1 0 0.0313

237 1 1 0 0.0313

239 1 1 0 0.0313

241 5 3 1 0.1563

242 1 1 0 0.0313

243 1 1 0 0.0313

244 1 1 0 0.0313

245 3 3 0 0.0938

247 4 4 0 0.1250

248 2 2 0 0.0625

249 1 1 0 0.0313

250 2 2 0 0.0625

252 1 1 0 0.0313

254 1 1 0 0.0313

257 2 2 0 0.0625

258 3 3 0 0.0938

268 1 1 0 0.0313

276 1 1 0 0.0313

Number of individuals typed: 16

Heterozygotes: 15

Homozygotes: 1

Number of alleles: 18

Observed heterozygosity: 0.9375

Expected heterozygosity: 0.9496

Polymorphic information content (PIC): 0.9146

Average non-exclusion probability (first parent): 0.2767

Average non-exclusion probability (second parent): 0.1608

Average non-exclusion probability (parent pair): 0.0421

Average non-exclusion probability (identity): 0.0118

Average non-exclusion probability (sib identity): 0.2930

**** Locus 1843/1844 ****

Allele Count Heterozygotes Homozygotes Frequency

195 1 1 0 0.0313

200 1 1 0 0.0313

202 1 1 0 0.0313

204 1 1 0 0.0313

205 1 1 0 0.0313

206 1 1 0 0.0313

207 1 1 0 0.0313

208 1 1 0 0.0313

222 1 1 0 0.0313

233 1 1 0 0.0313

234 1 1 0 0.0313

236 1 1 0 0.0313

237 1 1 0 0.0313

239 2 2 0 0.0625

256 2 2 0 0.0625

257 1 1 0 0.0313

265 2 2 0 0.0625

266 1 1 0 0.0313

269 1 1 0 0.0313

270 1 1 0 0.0313

271 1 1 0 0.0313

272 1 1 0 0.0313

275 1 1 0 0.0313

277 2 2 0 0.0625

278 1 1 0 0.0313

281 1 1 0 0.0313

294 1 1 0 0.0313

299 1 1 0 0.0313

Number of individuals typed: 16

Heterozygotes: 16

Homozygotes: 0

Number of alleles: 28

Observed heterozygosity: 1.0000

Expected heterozygosity: 0.9919

Polymorphic information content (PIC): 0.9595

Average non-exclusion probability (first parent): 0.1466

Average non-exclusion probability (second parent): 0.0791

Average non-exclusion probability (parent pair): 0.0114

Average non-exclusion probability (identity): 0.0030

Average non-exclusion probability (sib identity): 0.2703

**** Locus 1941/1942 ****

Allele Count Heterozygotes Homozygotes Frequency

145 2 2 0 0.0667

146 4 4 0 0.1333

147 2 2 0 0.0667

149 1 1 0 0.0333

150 12 10 1 0.4000

151 4 4 0 0.1333

152 4 4 0 0.1333

153 1 1 0 0.0333

Number of individuals typed: 15

Heterozygotes: 14

Homozygotes: 1

Number of alleles: 8

Observed heterozygosity: 0.9333

Expected heterozygosity: 0.8023

Polymorphic information content (PIC): 0.7518

Average non-exclusion probability (first parent): 0.5897

Average non-exclusion probability (second parent): 0.4075

Average non-exclusion probability (parent pair): 0.2113

Average non-exclusion probability (identity): 0.0742

Average non-exclusion probability (sib identity): 0.3808

**** Locus 1947/1948 ****

Allele Count Heterozygotes Homozygotes Frequency

188 1 1 0 0.0313

190 1 1 0 0.0313

193 1 1 0 0.0313

198 1 1 0 0.0313

204 2 2 0 0.0625

205 1 1 0 0.0313

206 4 4 0 0.1250

207 1 1 0 0.0313

209 1 1 0 0.0313

212 2 2 0 0.0625

213 5 5 0 0.1563

214 1 1 0 0.0313

217 1 1 0 0.0313

218 2 2 0 0.0625

219 1 1 0 0.0313

222 3 3 0 0.0938

223 1 1 0 0.0313

226 1 1 0 0.0313

230 1 1 0 0.0313

238 1 1 0 0.0313

Number of individuals typed: 16

Heterozygotes: 16

Homozygotes: 0

Number of alleles: 20

Observed heterozygosity: 1.0000

Expected heterozygosity: 0.9556

Polymorphic information content (PIC): 0.9212

Average non-exclusion probability (first parent): 0.2578

Average non-exclusion probability (second parent): 0.1484

Average non-exclusion probability (parent pair): 0.0360

Average non-exclusion probability (identity): 0.0100

Average non-exclusion probability (sib identity): 0.2896

**** Locus 1957/1958 ****

Allele Count Heterozygotes Homozygotes Frequency

289 1 1 0 0.0313

301 1 1 0 0.0313

302 1 1 0 0.0313

303 1 1 0 0.0313

306 2 2 0 0.0625

308 1 1 0 0.0313

311 1 1 0 0.0313

313 3 3 0 0.0938

314 1 1 0 0.0313

316 1 1 0 0.0313

318 1 1 0 0.0313

319 1 1 0 0.0313

321 1 1 0 0.0313

322 1 1 0 0.0313

323 1 1 0 0.0313

324 2 2 0 0.0625

325 1 1 0 0.0313

326 1 1 0 0.0313

328 1 1 0 0.0313

334 1 1 0 0.0313

335 1 1 0 0.0313

337 1 1 0 0.0313

339 1 1 0 0.0313

341 1 1 0 0.0313

356 1 1 0 0.0313

357 1 1 0 0.0313

359 1 1 0 0.0313

360 1 1 0 0.0313

Number of individuals typed: 16

Heterozygotes: 16

Homozygotes: 0

Number of alleles: 28

Observed heterozygosity: 1.0000

Expected heterozygosity: 0.9899

Polymorphic information content (PIC): 0.9574

Average non-exclusion probability (first parent): 0.1528

Average non-exclusion probability (second parent): 0.0828

Average non-exclusion probability (parent pair): 0.0123

Average non-exclusion probability (identity): 0.0032

Average non-exclusion probability (sib identity): 0.2713

**** Locus 1963/1964 ****

Allele Count Heterozygotes Homozygotes Frequency

191 1 1 0 0.0313

192 1 1 0 0.0313

193 2 2 0 0.0625

194 4 0 2 0.1250

195 5 1 2 0.1563

206 2 0 1 0.0625

207 4 4 0 0.1250

208 1 1 0 0.0313

209 1 1 0 0.0313

210 2 2 0 0.0625

211 1 1 0 0.0313

212 1 1 0 0.0313

213 1 1 0 0.0313

214 1 1 0 0.0313

217 3 3 0 0.0938

218 1 1 0 0.0313

222 1 1 0 0.0313

Number of individuals typed: 16

Heterozygotes: 11

Homozygotes: 5

Number of alleles: 17

Observed heterozygosity: 0.6875

Expected heterozygosity: 0.9435

Polymorphic information content (PIC): 0.9079

Average non-exclusion probability (first parent): 0.2943

Average non-exclusion probability (second parent): 0.1727

Average non-exclusion probability (parent pair): 0.0482

Average non-exclusion probability (identity): 0.0136

Average non-exclusion probability (sib identity): 0.2964

**** Locus 1983/1984 ****

Allele Count Heterozygotes Homozygotes Frequency

165 1 1 0 0.0313

178 1 1 0 0.0313

182 1 1 0 0.0313

184 6 2 2 0.1875

188 16 8 4 0.5000

190 3 3 0 0.0938

199 2 2 0 0.0625

204 1 1 0 0.0313

212 1 1 0 0.0313

Number of individuals typed: 16

Heterozygotes: 10

Homozygotes: 6

Number of alleles: 9

Observed heterozygosity: 0.6250

Expected heterozygosity: 0.7198

Polymorphic information content (PIC): 0.6695

Average non-exclusion probability (first parent): 0.6879

Average non-exclusion probability (second parent): 0.5021

Average non-exclusion probability (parent pair): 0.2939

Average non-exclusion probability (identity): 0.1195

Average non-exclusion probability (sib identity): 0.4312

**** Locus 2005/2006 ****

Allele Count Heterozygotes Homozygotes Frequency

208 1 1 0 0.0313

212 1 1 0 0.0313

254 1 1 0 0.0313

258 1 1 0 0.0313

259 1 1 0 0.0313

261 2 2 0 0.0625

263 1 1 0 0.0313

265 3 3 0 0.0938

267 4 4 0 0.1250

269 1 1 0 0.0313

271 1 1 0 0.0313

277 2 2 0 0.0625

278 1 1 0 0.0313

280 1 1 0 0.0313

281 2 2 0 0.0625

282 1 1 0 0.0313

283 1 1 0 0.0313

285 1 1 0 0.0313

288 1 1 0 0.0313

290 1 1 0 0.0313

298 1 1 0 0.0313

299 2 2 0 0.0625

300 1 1 0 0.0313

Number of individuals typed: 16

Heterozygotes: 16

Homozygotes: 0

Number of alleles: 23

Observed heterozygosity: 1.0000

Expected heterozygosity: 0.9738

Polymorphic information content (PIC): 0.9405

Average non-exclusion probability (first parent): 0.2043

Average non-exclusion probability (second parent): 0.1140

Average non-exclusion probability (parent pair): 0.0223

Average non-exclusion probability (identity): 0.0060

Average non-exclusion probability (sib identity): 0.2798

**** Locus 2081/2082 ****

Allele Count Heterozygotes Homozygotes Frequency

273 2 2 0 0.0625

295 2 2 0 0.0625

297 1 1 0 0.0313

298 1 1 0 0.0313

299 1 1 0 0.0313

300 1 1 0 0.0313

305 3 3 0 0.0938

306 3 3 0 0.0938

307 1 1 0 0.0313

308 2 2 0 0.0625

309 1 1 0 0.0313

310 3 3 0 0.0938

312 1 1 0 0.0313

315 1 1 0 0.0313

316 1 1 0 0.0313

318 2 2 0 0.0625

320 1 1 0 0.0313

328 1 1 0 0.0313

330 1 1 0 0.0313

353 1 1 0 0.0313

371 1 1 0 0.0313

383 1 1 0 0.0313

Number of individuals typed: 16

Heterozygotes: 16

Homozygotes: 0

Number of alleles: 22

Observed heterozygosity: 1.0000

Expected heterozygosity: 0.9738

Polymorphic information content (PIC): 0.9405

Average non-exclusion probability (first parent): 0.2054

Average non-exclusion probability (second parent): 0.1146

Average non-exclusion probability (parent pair): 0.0227

Average non-exclusion probability (identity): 0.0061

Average non-exclusion probability (sib identity): 0.2798

**** Locus 2107/2108 ****

Allele Count Heterozygotes Homozygotes Frequency

144 1 1 0 0.0333

186 1 1 0 0.0333

188 4 4 0 0.1333

189 1 1 0 0.0333

190 3 3 0 0.1000

191 3 3 0 0.1000

192 3 3 0 0.1000

194 1 1 0 0.0333

195 4 4 0 0.1333

196 1 1 0 0.0333

197 2 2 0 0.0667

198 1 1 0 0.0333

199 1 1 0 0.0333

200 1 1 0 0.0333

202 1 1 0 0.0333

204 1 1 0 0.0333

205 1 1 0 0.0333

Number of individuals typed: 15

Heterozygotes: 15

Homozygotes: 0

Number of alleles: 17

Observed heterozygosity: 1.0000

Expected heterozygosity: 0.9494

Polymorphic information content (PIC): 0.9120

Average non-exclusion probability (first parent): 0.2845

Average non-exclusion probability (second parent): 0.1659

Average non-exclusion probability (parent pair): 0.0450

Average non-exclusion probability (identity): 0.0126

Average non-exclusion probability (sib identity): 0.2943

**** Locus 2119/2120 ****

Allele Count Heterozygotes Homozygotes Frequency

270 3 1 1 0.0938

274 5 3 1 0.1563

279 3 3 0 0.0938

282 1 1 0 0.0313

287 2 2 0 0.0625

288 1 1 0 0.0313

292 2 2 0 0.0625

293 4 4 0 0.1250

294 1 1 0 0.0313

295 1 1 0 0.0313

296 1 1 0 0.0313

297 2 2 0 0.0625

298 2 2 0 0.0625

299 1 1 0 0.0313

301 1 1 0 0.0313

303 1 1 0 0.0313

304 1 1 0 0.0313

Number of individuals typed: 16

Heterozygotes: 14

Homozygotes: 2

Number of alleles: 17

Observed heterozygosity: 0.8750

Expected heterozygosity: 0.9476

Polymorphic information content (PIC): 0.9123

Average non-exclusion probability (first parent): 0.2832

Average non-exclusion probability (second parent): 0.1650

Average non-exclusion probability (parent pair): 0.0444

Average non-exclusion probability (identity): 0.0124

Average non-exclusion probability (sib identity): 0.2941

**** Locus 2139/2140 ****

Allele Count Heterozygotes Homozygotes Frequency

227 1 1 0 0.0333

231 3 3 0 0.1000

244 9 7 1 0.3000

245 1 1 0 0.0333

246 1 1 0 0.0333

250 4 4 0 0.1333

258 2 2 0 0.0667

261 2 2 0 0.0667

262 3 3 0 0.1000

268 1 1 0 0.0333

271 2 2 0 0.0667

276 1 1 0 0.0333

Number of individuals typed: 15

Heterozygotes: 14

Homozygotes: 1

Number of alleles: 12

Observed heterozygosity: 0.9333

Expected heterozygosity: 0.8828

Polymorphic information content (PIC): 0.8405

Average non-exclusion probability (first parent): 0.4399

Average non-exclusion probability (second parent): 0.2798

Average non-exclusion probability (parent pair): 0.1094

Average non-exclusion probability (identity): 0.0343

Average non-exclusion probability (sib identity): 0.3319

**** Locus 2147/2148 ****

Allele Count Heterozygotes Homozygotes Frequency

285 4 2 1 0.1250

286 1 1 0 0.0313

289 1 1 0 0.0313

291 6 2 2 0.1875

292 1 1 0 0.0313

293 5 1 2 0.1563

295 1 1 0 0.0313

300 8 4 2 0.2500

301 2 0 1 0.0625

302 1 1 0 0.0313

303 1 1 0 0.0313

306 1 1 0 0.0313

Number of individuals typed: 16

Heterozygotes: 8

Homozygotes: 8

Number of alleles: 12

Observed heterozygosity: 0.5000

Expected heterozygosity: 0.8790

Polymorphic information content (PIC): 0.8355

Average non-exclusion probability (first parent): 0.4539

Average non-exclusion probability (second parent): 0.2918

Average non-exclusion probability (parent pair): 0.1231

Average non-exclusion probability (identity): 0.0381

Average non-exclusion probability (sib identity): 0.3337

**** Locus 2153/2154 ****

Allele Count Heterozygotes Homozygotes Frequency

232 1 1 0 0.0313

233 3 3 0 0.0938

234 1 1 0 0.0313

235 3 3 0 0.0938

237 3 3 0 0.0938

239 1 1 0 0.0313

240 3 3 0 0.0938

241 2 2 0 0.0625

242 1 1 0 0.0313

243 3 3 0 0.0938

244 2 2 0 0.0625

245 3 3 0 0.0938

248 1 1 0 0.0313

250 1 1 0 0.0313

251 2 2 0 0.0625

252 1 1 0 0.0313

253 1 1 0 0.0313

Number of individuals typed: 16

Heterozygotes: 16

Homozygotes: 0

Number of alleles: 17

Observed heterozygosity: 1.0000

Expected heterozygosity: 0.9577

Polymorphic information content (PIC): 0.9230

Average non-exclusion probability (first parent): 0.2565

Average non-exclusion probability (second parent): 0.1469

Average non-exclusion probability (parent pair): 0.0364

Average non-exclusion probability (identity): 0.0099

Average non-exclusion probability (sib identity): 0.2886

**** Locus 2187/2188 ****

Allele Count Heterozygotes Homozygotes Frequency

152 2 2 0 0.0667

156 3 3 0 0.1000

157 2 2 0 0.0667

158 2 2 0 0.0667

166 6 6 0 0.2000

168 2 2 0 0.0667

170 7 7 0 0.2333

173 1 1 0 0.0333

174 1 1 0 0.0333

175 1 1 0 0.0333

176 1 1 0 0.0333

178 1 1 0 0.0333

179 1 1 0 0.0333

Number of individuals typed: 15

Heterozygotes: 15

Homozygotes: 0

Number of alleles: 13

Observed heterozygosity: 1.0000

Expected heterozygosity: 0.9011

Polymorphic information content (PIC): 0.8592

Average non-exclusion probability (first parent): 0.4041

Average non-exclusion probability (second parent): 0.2525

Average non-exclusion probability (parent pair): 0.0938

Average non-exclusion probability (identity): 0.0285

Average non-exclusion probability (sib identity): 0.3216

**** Locus 2199/2200 ****

Allele Count Heterozygotes Homozygotes Frequency

333 1 1 0 0.0313

335 1 1 0 0.0313

340 3 3 0 0.0938

341 3 3 0 0.0938

342 3 3 0 0.0938

343 6 6 0 0.1875

344 2 2 0 0.0625

345 5 5 0 0.1563

346 5 5 0 0.1563

347 1 1 0 0.0313

348 2 2 0 0.0625

Number of individuals typed: 16

Heterozygotes: 16

Homozygotes: 0

Number of alleles: 11

Observed heterozygosity: 1.0000

Expected heterozygosity: 0.9073

Polymorphic information content (PIC): 0.8669

Average non-exclusion probability (first parent): 0.3940

Average non-exclusion probability (second parent): 0.2439

Average non-exclusion probability (parent pair): 0.0912

Average non-exclusion probability (identity): 0.0266

Average non-exclusion probability (sib identity): 0.3172

**** Locus 2207/2208 ****

Allele Count Heterozygotes Homozygotes Frequency

221 3 3 0 0.1000

223 6 6 0 0.2000

224 1 1 0 0.0333

226 1 1 0 0.0333

227 4 2 1 0.1333

228 1 1 0 0.0333

238 14 14 0 0.4667

Number of individuals typed: 15

Heterozygotes: 14

Homozygotes: 1

Number of alleles: 7

Observed heterozygosity: 0.9333

Expected heterozygosity: 0.7356

Polymorphic information content (PIC): 0.6771

Average non-exclusion probability (first parent): 0.6845

Average non-exclusion probability (second parent): 0.5022

Average non-exclusion probability (parent pair): 0.3041

Average non-exclusion probability (identity): 0.1175

Average non-exclusion probability (sib identity): 0.4238

**** Locus 2209/2210 ****

Allele Count Heterozygotes Homozygotes Frequency

264 1 1 0 0.0333

265 4 4 0 0.1333

266 1 1 0 0.0333

268 2 0 1 0.0667

269 5 5 0 0.1667

270 7 3 2 0.2333

271 3 3 0 0.1000

272 4 4 0 0.1333

273 3 1 1 0.1000

Number of individuals typed: 15

Heterozygotes: 11

Homozygotes: 4

Number of alleles: 9

Observed heterozygosity: 0.7333

Expected heterozygosity: 0.8851

Polymorphic information content (PIC): 0.8393

Average non-exclusion probability (first parent): 0.4520

Average non-exclusion probability (second parent): 0.2892

Average non-exclusion probability (parent pair): 0.1234

Average non-exclusion probability (identity): 0.0371

Average non-exclusion probability (sib identity): 0.3315

**** Locus 2269/2270 ****

Allele Count Heterozygotes Homozygotes Frequency

209 1 1 0 0.0313

231 1 1 0 0.0313

234 1 1 0 0.0313

235 2 2 0 0.0625

236 2 2 0 0.0625

238 1 1 0 0.0313

239 6 6 0 0.1875

241 1 1 0 0.0313

242 2 2 0 0.0625

244 1 1 0 0.0313

245 3 3 0 0.0938

246 2 2 0 0.0625

248 1 1 0 0.0313

250 4 4 0 0.1250

251 1 1 0 0.0313

253 2 2 0 0.0625

262 1 1 0 0.0313

Number of individuals typed: 16

Heterozygotes: 16

Homozygotes: 0

Number of alleles: 17

Observed heterozygosity: 1.0000

Expected heterozygosity: 0.9415

Polymorphic information content (PIC): 0.9060

Average non-exclusion probability (first parent): 0.2976

Average non-exclusion probability (second parent): 0.1750

Average non-exclusion probability (parent pair): 0.0486

Average non-exclusion probability (identity): 0.0138

Average non-exclusion probability (sib identity): 0.2974

**** Locus 2279/2280 ****

Allele Count Heterozygotes Homozygotes Frequency

236 1 1 0 0.0313

237 2 2 0 0.0625

238 2 0 1 0.0625

239 10 2 4 0.3125

240 5 3 1 0.1563

241 2 0 1 0.0625

242 2 2 0 0.0625

243 3 1 1 0.0938

245 2 2 0 0.0625

246 1 1 0 0.0313

248 2 2 0 0.0625

Number of individuals typed: 16

Heterozygotes: 8

Homozygotes: 8

Number of alleles: 11

Observed heterozygosity: 0.5000

Expected heterozygosity: 0.8710

Polymorphic information content (PIC): 0.8296

Average non-exclusion probability (first parent): 0.4604

Average non-exclusion probability (second parent): 0.2961

Average non-exclusion probability (parent pair): 0.1207

Average non-exclusion probability (identity): 0.0385

Average non-exclusion probability (sib identity): 0.3378

**** Locus 2281/2282 ****

Allele Count Heterozygotes Homozygotes Frequency

274 1 1 0 0.0313

277 1 1 0 0.0313

285 2 2 0 0.0625

296 1 1 0 0.0313

300 2 2 0 0.0625

303 2 2 0 0.0625

304 1 1 0 0.0313

305 3 3 0 0.0938

306 2 2 0 0.0625

307 2 2 0 0.0625

308 8 8 0 0.2500

309 2 2 0 0.0625

310 1 1 0 0.0313

311 3 3 0 0.0938

312 1 1 0 0.0313

Number of individuals typed: 16

Heterozygotes: 16

Homozygotes: 0

Number of alleles: 15

Observed heterozygosity: 1.0000

Expected heterozygosity: 0.9194

Polymorphic information content (PIC): 0.8828

Average non-exclusion probability (first parent): 0.3504

Average non-exclusion probability (second parent): 0.2122

Average non-exclusion probability (parent pair): 0.0665

Average non-exclusion probability (identity): 0.0198

Average non-exclusion probability (sib identity): 0.3096

**** Locus 2289/2290 ****

Allele Count Heterozygotes Homozygotes Frequency

157 2 2 0 0.0625

158 1 1 0 0.0313

162 2 2 0 0.0625

164 1 1 0 0.0313

171 3 3 0 0.0938

172 1 1 0 0.0313

173 1 1 0 0.0313

174 5 5 0 0.1563

176 1 1 0 0.0313

177 2 2 0 0.0625

180 1 1 0 0.0313

186 1 1 0 0.0313

187 2 2 0 0.0625

189 7 7 0 0.2188

190 1 1 0 0.0313

192 1 1 0 0.0313

Number of individuals typed: 16

Heterozygotes: 16

Homozygotes: 0

Number of alleles: 16

Observed heterozygosity: 1.0000

Expected heterozygosity: 0.9234

Polymorphic information content (PIC): 0.8864

Average non-exclusion probability (first parent): 0.3433

Average non-exclusion probability (second parent): 0.2074

Average non-exclusion probability (parent pair): 0.0653

Average non-exclusion probability (identity): 0.0192

Average non-exclusion probability (sib identity): 0.3075

**** Locus 2297/2298 ****

Allele Count Heterozygotes Homozygotes Frequency

177 1 1 0 0.0313

178 1 1 0 0.0313

183 1 1 0 0.0313

189 2 2 0 0.0625

190 3 3 0 0.0938

192 1 1 0 0.0313

201 1 1 0 0.0313

202 2 2 0 0.0625

205 1 1 0 0.0313

207 3 3 0 0.0938

211 3 3 0 0.0938

212 9 9 0 0.2813

214 1 1 0 0.0313

215 2 2 0 0.0625

218 1 1 0 0.0313

Number of individuals typed: 16

Heterozygotes: 16

Homozygotes: 0

Number of alleles: 15

Observed heterozygosity: 1.0000

Expected heterozygosity: 0.9032

Polymorphic information content (PIC): 0.8659

Average non-exclusion probability (first parent): 0.3856

Average non-exclusion probability (second parent): 0.2382

Average non-exclusion probability (parent pair): 0.0804

Average non-exclusion probability (identity): 0.0247

Average non-exclusion probability (sib identity): 0.3187

**** Locus 2323/2324 ****

Allele Count Heterozygotes Homozygotes Frequency

224 1 1 0 0.0313

227 1 1 0 0.0313

254 6 6 0 0.1875

255 1 1 0 0.0313

256 3 1 1 0.0938

257 6 6 0 0.1875

258 3 3 0 0.0938

260 4 4 0 0.1250

261 5 5 0 0.1563

265 1 1 0 0.0313

266 1 1 0 0.0313

Number of individuals typed: 16

Heterozygotes: 15

Homozygotes: 1

Number of alleles: 11

Observed heterozygosity: 0.9375

Expected heterozygosity: 0.8952

Polymorphic information content (PIC): 0.8530

Average non-exclusion probability (first parent): 0.4234

Average non-exclusion probability (second parent): 0.2667

Average non-exclusion probability (parent pair): 0.1070

Average non-exclusion probability (identity): 0.0318

Average non-exclusion probability (sib identity): 0.3244

**** Locus 2361/2362 ****

Allele Count Heterozygotes Homozygotes Frequency

251 2 0 1 0.0625

268 3 3 0 0.0938

269 12 2 5 0.3750

271 1 1 0 0.0313

272 2 2 0 0.0625

273 1 1 0 0.0313

274 10 4 3 0.3125

277 1 1 0 0.0313

Number of individuals typed: 16

Heterozygotes: 7

Homozygotes: 9

Number of alleles: 8

Observed heterozygosity: 0.4375

Expected heterozygosity: 0.7661

Polymorphic information content (PIC): 0.7051

Average non-exclusion probability (first parent): 0.6480

Average non-exclusion probability (second parent): 0.4708

Average non-exclusion probability (parent pair): 0.2779

Average non-exclusion probability (identity): 0.1035

Average non-exclusion probability (sib identity): 0.4048

**** Locus 2379/2380 ****

Allele Count Heterozygotes Homozygotes Frequency

305 1 1 0 0.0313

315 1 1 0 0.0313

316 1 1 0 0.0313

320 1 1 0 0.0313

323 1 1 0 0.0313

324 1 1 0 0.0313

325 6 4 1 0.1875

326 2 2 0 0.0625

327 6 2 2 0.1875

328 2 2 0 0.0625

329 2 2 0 0.0625

330 2 2 0 0.0625

334 3 1 1 0.0938

336 2 2 0 0.0625

348 1 1 0 0.0313

Number of individuals typed: 16

Heterozygotes: 12

Homozygotes: 4

Number of alleles: 15

Observed heterozygosity: 0.7500

Expected heterozygosity: 0.9234

Polymorphic information content (PIC): 0.8860

Average non-exclusion probability (first parent): 0.3458

Average non-exclusion probability (second parent): 0.2090

Average non-exclusion probability (parent pair): 0.0671

Average non-exclusion probability (identity): 0.0196

Average non-exclusion probability (sib identity): 0.3076

**** Locus 2389/2390 ****

Allele Count Heterozygotes Homozygotes Frequency

230 2 2 0 0.0625

234 1 1 0 0.0313

236 1 1 0 0.0313

239 11 9 1 0.3438

242 1 1 0 0.0313

243 1 1 0 0.0313

244 3 3 0 0.0938

245 1 1 0 0.0313

248 2 2 0 0.0625

250 1 1 0 0.0313

251 3 1 1 0.0938

253 2 2 0 0.0625

254 1 1 0 0.0313

255 1 1 0 0.0313

257 1 1 0 0.0313

Number of individuals typed: 16

Heterozygotes: 14

Homozygotes: 2

Number of alleles: 15

Observed heterozygosity: 0.8750

Expected heterozygosity: 0.8710

Polymorphic information content (PIC): 0.8335

Average non-exclusion probability (first parent): 0.4456

Average non-exclusion probability (second parent): 0.2839

Average non-exclusion probability (parent pair): 0.1050

Average non-exclusion probability (identity): 0.0347

Average non-exclusion probability (sib identity): 0.3368

**** Locus 2397/2398 ****

Allele Count Heterozygotes Homozygotes Frequency

161 2 2 0 0.0625

164 4 2 1 0.1250

165 1 1 0 0.0313

166 4 2 1 0.1250

169 7 5 1 0.2188

172 7 7 0 0.2188

176 5 5 0 0.1563

188 1 1 0 0.0313

189 1 1 0 0.0313

Number of individuals typed: 16

Heterozygotes: 13

Homozygotes: 3

Number of alleles: 9

Observed heterozygosity: 0.8125

Expected heterozygosity: 0.8690

Polymorphic information content (PIC): 0.8225

Average non-exclusion probability (first parent): 0.4838

Average non-exclusion probability (second parent): 0.3158

Average non-exclusion probability (parent pair): 0.1445

Average non-exclusion probability (identity): 0.0444

Average non-exclusion probability (sib identity): 0.3402

**** Locus 2473/2474 ****

Allele Count Heterozygotes Homozygotes Frequency

138 2 2 0 0.0625

139 1 1 0 0.0313

141 1 1 0 0.0313

142 3 1 1 0.0938

143 4 2 1 0.1250

144 4 4 0 0.1250

146 1 1 0 0.0313

149 1 1 0 0.0313

150 5 5 0 0.1563

152 3 3 0 0.0938

153 3 3 0 0.0938

155 1 1 0 0.0313

167 2 2 0 0.0625

169 1 1 0 0.0313

Number of individuals typed: 16

Heterozygotes: 14

Homozygotes: 2

Number of alleles: 14

Observed heterozygosity: 0.8750

Expected heterozygosity: 0.9335

Polymorphic information content (PIC): 0.8965

Average non-exclusion probability (first parent): 0.3251

Average non-exclusion probability (second parent): 0.1936

Average non-exclusion probability (parent pair): 0.0601

Average non-exclusion probability (identity): 0.0170

Average non-exclusion probability (sib identity): 0.3021

**** Locus 2487/2488 ****

Allele Count Heterozygotes Homozygotes Frequency

277 4 2 1 0.1250

278 1 1 0 0.0313

279 2 2 0 0.0625

280 3 3 0 0.0938

281 2 2 0 0.0625

282 5 3 1 0.1563

283 9 5 2 0.2813

284 2 2 0 0.0625

285 2 0 1 0.0625

292 2 2 0 0.0625

Number of individuals typed: 16

Heterozygotes: 11

Homozygotes: 5

Number of alleles: 10

Observed heterozygosity: 0.6875

Expected heterozygosity: 0.8790

Polymorphic information content (PIC): 0.8368

Average non-exclusion probability (first parent): 0.4511

Average non-exclusion probability (second parent): 0.2886

Average non-exclusion probability (parent pair): 0.1188

Average non-exclusion probability (identity): 0.0368

Average non-exclusion probability (sib identity): 0.3334

**** Locus 2491/2492 ****

Allele Count Heterozygotes Homozygotes Frequency

294 3 3 0 0.1000

301 2 2 0 0.0667

302 3 1 1 0.1000

303 1 1 0 0.0333

307 1 1 0 0.0333

312 1 1 0 0.0333

313 1 1 0 0.0333

314 2 2 0 0.0667

315 4 2 1 0.1333

316 2 2 0 0.0667

319 2 0 1 0.0667

321 4 2 1 0.1333

322 3 1 1 0.1000

329 1 1 0 0.0333

Number of individuals typed: 15

Heterozygotes: 10

Homozygotes: 5

Number of alleles: 14

Observed heterozygosity: 0.6667

Expected heterozygosity: 0.9425

Polymorphic information content (PIC): 0.9042

Average non-exclusion probability (first parent): 0.3064

Average non-exclusion probability (second parent): 0.1804

Average non-exclusion probability (parent pair): 0.0530

Average non-exclusion probability (identity): 0.0148

Average non-exclusion probability (sib identity): 0.2981

**** Locus 2517/2518 ****

Allele Count Heterozygotes Homozygotes Frequency

213 1 1 0 0.0333

215 1 1 0 0.0333

216 1 1 0 0.0333

218 2 2 0 0.0667

219 6 6 0 0.2000

221 1 1 0 0.0333

223 1 1 0 0.0333

224 1 1 0 0.0333

225 5 5 0 0.1667

226 1 1 0 0.0333

227 3 3 0 0.1000

228 4 4 0 0.1333

229 1 1 0 0.0333

230 1 1 0 0.0333

234 1 1 0 0.0333

Number of individuals typed: 15

Heterozygotes: 15

Homozygotes: 0

Number of alleles: 15

Observed heterozygosity: 1.0000

Expected heterozygosity: 0.9195

Polymorphic information content (PIC): 0.8794

Average non-exclusion probability (first parent): 0.3615

Average non-exclusion probability (second parent): 0.2206

Average non-exclusion probability (parent pair): 0.0744

Average non-exclusion probability (identity): 0.0219

Average non-exclusion probability (sib identity): 0.3110

**** Locus 2527/2528 ****

Allele Count Heterozygotes Homozygotes Frequency

206 4 0 2 0.1250

207 6 2 2 0.1875

208 6 0 3 0.1875

210 3 1 1 0.0938

214 2 2 0 0.0625

217 3 1 1 0.0938

218 3 1 1 0.0938

219 1 1 0 0.0313

225 1 1 0 0.0313

226 1 1 0 0.0313

227 1 1 0 0.0313

228 1 1 0 0.0313

Number of individuals typed: 16

Heterozygotes: 6

Homozygotes: 10

Number of alleles: 12

Observed heterozygosity: 0.3750

Expected heterozygosity: 0.9073

Polymorphic information content (PIC): 0.8672

Average non-exclusion probability (first parent): 0.3919

Average non-exclusion probability (second parent): 0.2426

Average non-exclusion probability (parent pair): 0.0897

Average non-exclusion probability (identity): 0.0264

Average non-exclusion probability (sib identity): 0.3171

**** Locus 2535/2536 ****

Allele Count Heterozygotes Homozygotes Frequency

235 1 1 0 0.0333

238 1 1 0 0.0333

240 2 2 0 0.0667

241 8 0 4 0.2667

242 8 4 2 0.2667

244 10 0 5 0.3333

Number of individuals typed: 15

Heterozygotes: 4

Homozygotes: 11

Number of alleles: 6

Observed heterozygosity: 0.2667

Expected heterozygosity: 0.7655

Polymorphic information content (PIC): 0.6949

Average non-exclusion probability (first parent): 0.6709

Average non-exclusion probability (second parent): 0.4966

Average non-exclusion probability (parent pair): 0.3164

Average non-exclusion probability (identity): 0.1127

Average non-exclusion probability (sib identity): 0.4082

**** Locus 2537/2538 ****

Allele Count Heterozygotes Homozygotes Frequency

240 1 1 0 0.0333

245 1 1 0 0.0333

247 1 1 0 0.0333

249 4 4 0 0.1333

252 1 1 0 0.0333

253 3 3 0 0.1000

254 1 1 0 0.0333

255 3 3 0 0.1000

256 1 1 0 0.0333

257 1 1 0 0.0333

258 7 3 2 0.2333

260 4 4 0 0.1333

261 1 1 0 0.0333

264 1 1 0 0.0333

Number of individuals typed: 15

Heterozygotes: 13

Homozygotes: 2

Number of alleles: 14

Observed heterozygosity: 0.8667

Expected heterozygosity: 0.9103

Polymorphic information content (PIC): 0.8694

Average non-exclusion probability (first parent): 0.3835

Average non-exclusion probability (second parent): 0.2367

Average non-exclusion probability (parent pair): 0.0839

Average non-exclusion probability (identity): 0.0250

Average non-exclusion probability (sib identity): 0.3162

**** Locus 2599/2600 ****

Allele Count Heterozygotes Homozygotes Frequency

202 1 1 0 0.0313

204 1 1 0 0.0313

206 2 2 0 0.0625

207 10 4 3 0.3125

209 6 2 2 0.1875

211 3 3 0 0.0938

213 1 1 0 0.0313

215 1 1 0 0.0313

216 1 1 0 0.0313

219 2 2 0 0.0625

220 2 0 1 0.0625

221 2 0 1 0.0625

Number of individuals typed: 16

Heterozygotes: 9

Homozygotes: 7

Number of alleles: 12

Observed heterozygosity: 0.5625

Expected heterozygosity: 0.8649

Polymorphic information content (PIC): 0.8225

Average non-exclusion probability (first parent): 0.4724

Average non-exclusion probability (second parent): 0.3066

Average non-exclusion probability (parent pair): 0.1287

Average non-exclusion probability (identity): 0.0416

Average non-exclusion probability (sib identity): 0.3415

**** Locus 2615/2616 ****

Allele Count Heterozygotes Homozygotes Frequency

132 1 1 0 0.0313

136 1 1 0 0.0313

138 3 3 0 0.0938

141 2 2 0 0.0625

142 4 4 0 0.1250

143 3 3 0 0.0938

146 5 5 0 0.1563

147 1 1 0 0.0313

148 5 5 0 0.1563

149 1 1 0 0.0313

151 1 1 0 0.0313

152 1 1 0 0.0313

154 1 1 0 0.0313

155 3 3 0 0.0938

Number of individuals typed: 16

Heterozygotes: 16

Homozygotes: 0

Number of alleles: 14

Observed heterozygosity: 1.0000

Expected heterozygosity: 0.9274

Polymorphic information content (PIC): 0.8898

Average non-exclusion probability (first parent): 0.3406

Average non-exclusion probability (second parent): 0.2048

Average non-exclusion probability (parent pair): 0.0663

Average non-exclusion probability (identity): 0.0189

Average non-exclusion probability (sib identity): 0.3055

**** Locus 2623/2624 ****

Allele Count Heterozygotes Homozygotes Frequency

112 1 1 0 0.0333

119 1 1 0 0.0333

151 1 1 0 0.0333

152 2 2 0 0.0667

153 2 2 0 0.0667

154 4 4 0 0.1333

155 2 2 0 0.0667

164 1 1 0 0.0333

165 4 4 0 0.1333

167 7 7 0 0.2333

168 3 3 0 0.1000

171 2 2 0 0.0667

Number of individuals typed: 15

Heterozygotes: 15

Homozygotes: 0

Number of alleles: 12

Observed heterozygosity: 1.0000

Expected heterozygosity: 0.9080

Polymorphic information content (PIC): 0.8666

Average non-exclusion probability (first parent): 0.3912

Average non-exclusion probability (second parent): 0.2421

Average non-exclusion probability (parent pair): 0.0880

Average non-exclusion probability (identity): 0.0261

Average non-exclusion probability (sib identity): 0.3176

**** Locus 2787/2788 ****

Allele Count Heterozygotes Homozygotes Frequency

148 1 1 0 0.0313

152 2 2 0 0.0625

175 1 1 0 0.0313

176 10 6 2 0.3125

177 5 1 2 0.1563

178 4 0 2 0.1250

179 5 3 1 0.1563

180 2 2 0 0.0625

181 1 1 0 0.0313

187 1 1 0 0.0313

Number of individuals typed: 16

Heterozygotes: 9

Homozygotes: 7

Number of alleles: 10

Observed heterozygosity: 0.5625

Expected heterozygosity: 0.8528

Polymorphic information content (PIC): 0.8070

Average non-exclusion probability (first parent): 0.5051

Average non-exclusion probability (second parent): 0.3337

Average non-exclusion probability (parent pair): 0.1535

Average non-exclusion probability (identity): 0.0494

Average non-exclusion probability (sib identity): 0.3493

**** Locus 2847/2848 ****

Allele Count Heterozygotes Homozygotes Frequency

137 3 3 0 0.0938

139 2 2 0 0.0625

140 2 2 0 0.0625

141 1 1 0 0.0313

142 5 5 0 0.1563

144 2 2 0 0.0625

145 3 3 0 0.0938

146 1 1 0 0.0313

147 6 6 0 0.1875

149 1 1 0 0.0313

150 1 1 0 0.0313

153 4 4 0 0.1250

154 1 1 0 0.0313

Number of individuals typed: 16

Heterozygotes: 16

Homozygotes: 0

Number of alleles: 13

Observed heterozygosity: 1.0000

Expected heterozygosity: 0.9194

Polymorphic information content (PIC): 0.8809

Average non-exclusion probability (first parent): 0.3608

Average non-exclusion probability (second parent): 0.2195

Average non-exclusion probability (parent pair): 0.0748

Average non-exclusion probability (identity): 0.0216

Average non-exclusion probability (sib identity): 0.3101

**** Locus 2857/2858 ****

Allele Count Heterozygotes Homozygotes Frequency

123 1 1 0 0.0333

128 1 1 0 0.0333

129 1 1 0 0.0333

132 1 1 0 0.0333

133 9 9 0 0.3000

135 5 5 0 0.1667

136 7 7 0 0.2333

137 1 1 0 0.0333

143 3 3 0 0.1000

144 1 1 0 0.0333

Number of individuals typed: 15

Heterozygotes: 15

Homozygotes: 0

Number of alleles: 10

Observed heterozygosity: 1.0000

Expected heterozygosity: 0.8391

Polymorphic information content (PIC): 0.7874

Average non-exclusion probability (first parent): 0.5378

Average non-exclusion probability (second parent): 0.3637

Average non-exclusion probability (parent pair): 0.1804

Average non-exclusion probability (identity): 0.0594

Average non-exclusion probability (sib identity): 0.3593

**** Locus 2861/2862 ****

Allele Count Heterozygotes Homozygotes Frequency

126 1 1 0 0.0313

145 1 1 0 0.0313

174 1 1 0 0.0313

182 1 1 0 0.0313

183 1 1 0 0.0313

185 4 4 0 0.1250

187 8 8 0 0.2500

188 2 2 0 0.0625

189 1 1 0 0.0313

192 1 1 0 0.0313

193 1 1 0 0.0313

198 1 1 0 0.0313

199 2 2 0 0.0625

201 1 1 0 0.0313

202 1 1 0 0.0313

203 2 2 0 0.0625

205 1 1 0 0.0313

207 1 1 0 0.0313

209 1 1 0 0.0313

Number of individuals typed: 16

Heterozygotes: 16

Homozygotes: 0

Number of alleles: 19

Observed heterozygosity: 1.0000

Expected heterozygosity: 0.9254

Polymorphic information content (PIC): 0.8900

Average non-exclusion probability (first parent): 0.3303

Average non-exclusion probability (second parent): 0.1985

Average non-exclusion probability (parent pair): 0.0575

Average non-exclusion probability (identity): 0.0172

Average non-exclusion probability (sib identity): 0.3061

**** Locus 2869/2870 ****

Allele Count Heterozygotes Homozygotes Frequency

290 1 1 0 0.0333

302 1 1 0 0.0333

309 1 1 0 0.0333

318 2 2 0 0.0667

321 1 1 0 0.0333

324 4 2 1 0.1333

325 1 1 0 0.0333

326 2 2 0 0.0667

329 1 1 0 0.0333

334 2 0 1 0.0667

335 2 2 0 0.0667

342 3 3 0 0.1000

343 1 1 0 0.0333

347 2 2 0 0.0667

352 1 1 0 0.0333

354 1 1 0 0.0333

356 1 1 0 0.0333

407 3 3 0 0.1000

Number of individuals typed: 15

Heterozygotes: 13

Homozygotes: 2

Number of alleles: 18

Observed heterozygosity: 0.8667

Expected heterozygosity: 0.9609

Polymorphic information content (PIC): 0.9245

Average non-exclusion probability (first parent): 0.2513

Average non-exclusion probability (second parent): 0.1437

Average non-exclusion probability (parent pair): 0.0346

Average non-exclusion probability (identity): 0.0095

Average non-exclusion probability (sib identity): 0.2879

**** Locus 2895/2896 ****

Allele Count Heterozygotes Homozygotes Frequency

310 4 4 0 0.1250

313 1 1 0 0.0313

316 1 1 0 0.0313

318 1 1 0 0.0313

320 1 1 0 0.0313

334 1 1 0 0.0313

336 2 2 0 0.0625

337 2 2 0 0.0625

338 1 1 0 0.0313

346 4 2 1 0.1250

353 1 1 0 0.0313

356 1 1 0 0.0313

369 1 1 0 0.0313

379 1 1 0 0.0313

382 2 2 0 0.0625

391 1 1 0 0.0313

392 1 1 0 0.0313

398 1 1 0 0.0313

412 3 3 0 0.0938

413 2 2 0 0.0625

Number of individuals typed: 16

Heterozygotes: 15

Homozygotes: 1

Number of alleles: 20

Observed heterozygosity: 0.9375

Expected heterozygosity: 0.9617

Polymorphic information content (PIC): 0.9276

Average non-exclusion probability (first parent): 0.2416

Average non-exclusion probability (second parent): 0.1376

Average non-exclusion probability (parent pair): 0.0317

Average non-exclusion probability (identity): 0.0087

Average non-exclusion probability (sib identity): 0.2864

**** Locus 2895/2896 ****

Allele Count Heterozygotes Homozygotes Frequency

262 1 1 0 0.0313

265 1 1 0 0.0313

278 1 1 0 0.0313

303 2 2 0 0.0625

306 1 1 0 0.0313

312 2 2 0 0.0625

315 1 1 0 0.0313

316 1 1 0 0.0313

319 1 1 0 0.0313

320 1 1 0 0.0313

324 1 1 0 0.0313

328 1 1 0 0.0313

329 1 1 0 0.0313

331 1 1 0 0.0313

334 5 5 0 0.1563

337 2 2 0 0.0625

338 1 1 0 0.0313

347 1 1 0 0.0313

348 1 1 0 0.0313

363 1 1 0 0.0313

364 1 1 0 0.0313

404 2 2 0 0.0625

413 2 2 0 0.0625

Number of individuals typed: 16

Heterozygotes: 16

Homozygotes: 0

Number of alleles: 23

Observed heterozygosity: 1.0000

Expected heterozygosity: 0.9698

Polymorphic information content (PIC): 0.9365

Average non-exclusion probability (first parent): 0.2147

Average non-exclusion probability (second parent): 0.1208

Average non-exclusion probability (parent pair): 0.0243

Average non-exclusion probability (identity): 0.0066

Average non-exclusion probability (sib identity): 0.2819

**** Locus 2955/2956 ****

Allele Count Heterozygotes Homozygotes Frequency

318 2 0 1 0.0625

321 4 0 2 0.1250

322 2 0 1 0.0625

323 1 1 0 0.0313

325 1 1 0 0.0313

334 1 1 0 0.0313

335 4 0 2 0.1250

336 4 4 0 0.1250

337 3 1 1 0.0938

338 1 1 0 0.0313

339 2 2 0 0.0625

341 2 0 1 0.0625

342 1 1 0 0.0313

343 2 2 0 0.0625

347 1 1 0 0.0313

348 1 1 0 0.0313

Number of individuals typed: 16

Heterozygotes: 8

Homozygotes: 8

Number of alleles: 16

Observed heterozygosity: 0.5000

Expected heterozygosity: 0.9476

Polymorphic information content (PIC): 0.9121

Average non-exclusion probability (first parent): 0.2849

Average non-exclusion probability (second parent): 0.1659

Average non-exclusion probability (parent pair): 0.0453

Average non-exclusion probability (identity): 0.0126

Average non-exclusion probability (sib identity): 0.2942

**** Locus 2973/2974 ****

Allele Count Heterozygotes Homozygotes Frequency

127 1 1 0 0.0313

129 1 1 0 0.0313

135 1 1 0 0.0313

137 2 2 0 0.0625

138 1 1 0 0.0313

139 2 2 0 0.0625

140 1 1 0 0.0313

142 1 1 0 0.0313

145 1 1 0 0.0313

146 4 4 0 0.1250

148 1 1 0 0.0313

149 1 1 0 0.0313

151 2 2 0 0.0625

153 1 1 0 0.0313

156 1 1 0 0.0313

157 1 1 0 0.0313

164 1 1 0 0.0313

165 1 1 0 0.0313

167 2 2 0 0.0625

168 1 1 0 0.0313

169 2 2 0 0.0625

181 1 1 0 0.0313

182 1 1 0 0.0313

185 1 1 0 0.0313

Number of individuals typed: 16

Heterozygotes: 16

Homozygotes: 0

Number of alleles: 24

Observed heterozygosity: 1.0000

Expected heterozygosity: 0.9778

Polymorphic information content (PIC): 0.9448

Average non-exclusion probability (first parent): 0.1915

Average non-exclusion probability (second parent): 0.1062

Average non-exclusion probability (parent pair): 0.0195

Average non-exclusion probability (identity): 0.0052

Average non-exclusion probability (sib identity): 0.2777

**** Locus 2979/2980 ****

Allele Count Heterozygotes Homozygotes Frequency

146 2 0 1 0.0769

147 2 0 1 0.0769

150 2 0 1 0.0769

154 1 1 0 0.0385

252 3 3 0 0.1154

253 2 0 1 0.0769

254 3 1 1 0.1154

256 3 1 1 0.1154

257 1 1 0 0.0385

296 1 1 0 0.0385

306 1 1 0 0.0385

309 1 1 0 0.0385

340 1 1 0 0.0385

341 1 1 0 0.0385

342 1 1 0 0.0385

352 1 1 0 0.0385

Number of individuals typed: 13

Heterozygotes: 7

Homozygotes: 6

Number of alleles: 16

Observed heterozygosity: 0.5385

Expected heterozygosity: 0.9600

Polymorphic information content (PIC): 0.9179

Average non-exclusion probability (first parent): 0.2702

Average non-exclusion probability (second parent): 0.1560

Average non-exclusion probability (parent pair): 0.0405

Average non-exclusion probability (identity): 0.0111

Average non-exclusion probability (sib identity): 0.2912

**** Locus 2987/2988 ****

Allele Count Heterozygotes Homozygotes Frequency

225 8 0 4 0.2667

232 1 1 0 0.0333

296 1 1 0 0.0333

300 1 1 0 0.0333

302 1 1 0 0.0333

305 6 2 2 0.2000

306 2 2 0 0.0667

307 4 0 2 0.1333

309 1 1 0 0.0333

340 1 1 0 0.0333

341 1 1 0 0.0333

342 1 1 0 0.0333

350 1 1 0 0.0333

352 1 1 0 0.0333

Number of individuals typed: 15

Heterozygotes: 7

Homozygotes: 8

Number of alleles: 14

Observed heterozygosity: 0.4667

Expected heterozygosity: 0.8851

Polymorphic information content (PIC): 0.8417

Average non-exclusion probability (first parent): 0.4371

Average non-exclusion probability (second parent): 0.2787

Average non-exclusion probability (parent pair): 0.1106

Average non-exclusion probability (identity): 0.0347

Average non-exclusion probability (sib identity): 0.3309

**** Locus 3017/3018 ****

Allele Count Heterozygotes Homozygotes Frequency

132 3 1 1 0.0938

135 2 2 0 0.0625

136 4 4 0 0.1250

137 4 0 2 0.1250

138 2 0 1 0.0625

139 11 5 3 0.3438

142 5 3 1 0.1563

145 1 1 0 0.0313

Number of individuals typed: 16

Heterozygotes: 8

Homozygotes: 8

Number of alleles: 8

Observed heterozygosity: 0.5000

Expected heterozygosity: 0.8347

Polymorphic information content (PIC): 0.7871

Average non-exclusion probability (first parent): 0.5391

Average non-exclusion probability (second parent): 0.3625

Average non-exclusion probability (parent pair): 0.1763

Average non-exclusion probability (identity): 0.0581

Average non-exclusion probability (sib identity): 0.3602

**** Locus 3027/3028 ****

Allele Count Heterozygotes Homozygotes Frequency

321 2 2 0 0.0625

340 1 1 0 0.0313

343 4 2 1 0.1250

344 1 1 0 0.0313

345 5 5 0 0.1563

347 3 3 0 0.0938

348 4 4 0 0.1250

349 2 2 0 0.0625

350 4 2 1 0.1250

351 1 1 0 0.0313

352 1 1 0 0.0313

353 1 1 0 0.0313

357 1 1 0 0.0313

363 2 2 0 0.0625

Number of individuals typed: 16

Heterozygotes: 14

Homozygotes: 2

Number of alleles: 14

Observed heterozygosity: 0.8750

Expected heterozygosity: 0.9315

Polymorphic information content (PIC): 0.8943

Average non-exclusion probability (first parent): 0.3303

Average non-exclusion probability (second parent): 0.1973

Average non-exclusion probability (parent pair): 0.0621

Average non-exclusion probability (identity): 0.0176

Average non-exclusion probability (sib identity): 0.3032

**** Locus 3029/3030 ****

Allele Count Heterozygotes Homozygotes Frequency

99 2 2 0 0.0625

111 10 0 5 0.3125

113 1 1 0 0.0313

115 2 2 0 0.0625

117 3 3 0 0.0938

120 2 2 0 0.0625

123 1 1 0 0.0313

126 3 3 0 0.0938

134 1 1 0 0.0313

141 1 1 0 0.0313

142 1 1 0 0.0313

145 2 2 0 0.0625

146 1 1 0 0.0313

163 1 1 0 0.0313

164 1 1 0 0.0313

Number of individuals typed: 16

Heterozygotes: 11

Homozygotes: 5

Number of alleles: 15

Observed heterozygosity: 0.6875

Expected heterozygosity: 0.8891

Polymorphic information content (PIC): 0.8519

Average non-exclusion probability (first parent): 0.4120

Average non-exclusion probability (second parent): 0.2580

Average non-exclusion probability (parent pair): 0.0903

Average non-exclusion probability (identity): 0.0287

Average non-exclusion probability (sib identity): 0.3265

**** Locus 3051/3052 ****

Allele Count Heterozygotes Homozygotes Frequency

185 1 1 0 0.0313

191 3 3 0 0.0938

199 2 0 1 0.0625

202 7 5 1 0.2188

203 1 1 0 0.0313

205 1 1 0 0.0313

209 2 0 1 0.0625

210 1 1 0 0.0313

211 1 1 0 0.0313

213 1 1 0 0.0313

215 8 0 4 0.2500

221 1 1 0 0.0313

238 1 1 0 0.0313

254 1 1 0 0.0313

271 1 1 0 0.0313

Number of individuals typed: 16

Heterozygotes: 9

Homozygotes: 7

Number of alleles: 15

Observed heterozygosity: 0.5625

Expected heterozygosity: 0.8911

Polymorphic information content (PIC): 0.8509

Average non-exclusion probability (first parent): 0.4176

Average non-exclusion probability (second parent): 0.2636

Average non-exclusion probability (parent pair): 0.0995

Average non-exclusion probability (identity): 0.0311

Average non-exclusion probability (sib identity): 0.3261

**** Locus 3091/3092 ****

Allele Count Heterozygotes Homozygotes Frequency

304 3 1 1 0.1000

313 4 4 0 0.1333

315 3 3 0 0.1000

316 7 5 1 0.2333

317 1 1 0 0.0333

318 4 4 0 0.1333

319 2 2 0 0.0667

324 1 1 0 0.0333

330 1 1 0 0.0333

331 1 1 0 0.0333

343 1 1 0 0.0333

344 1 1 0 0.0333

346 1 1 0 0.0333

Number of individuals typed: 15

Heterozygotes: 13

Homozygotes: 2

Number of alleles: 13

Observed heterozygosity: 0.8667

Expected heterozygosity: 0.9080

Polymorphic information content (PIC): 0.8667

Average non-exclusion probability (first parent): 0.3905

Average non-exclusion probability (second parent): 0.2417

Average non-exclusion probability (parent pair): 0.0876

Average non-exclusion probability (identity): 0.0261

Average non-exclusion probability (sib identity): 0.3176

**** Locus 3119/3120 ****

Allele Count Heterozygotes Homozygotes Frequency

223 1 1 0 0.0313

230 1 1 0 0.0313

231 1 1 0 0.0313

233 1 1 0 0.0313

234 7 3 2 0.2188

235 3 1 1 0.0938

236 1 1 0 0.0313

240 1 1 0 0.0313

245 5 5 0 0.1563

274 1 1 0 0.0313

275 2 2 0 0.0625

276 2 2 0 0.0625

278 1 1 0 0.0313

280 4 4 0 0.1250

281 1 1 0 0.0313

Number of individuals typed: 16

Heterozygotes: 13

Homozygotes: 3

Number of alleles: 15

Observed heterozygosity: 0.8125

Expected heterozygosity: 0.9153

Polymorphic information content (PIC): 0.8771

Average non-exclusion probability (first parent): 0.3659

Average non-exclusion probability (second parent): 0.2238

Average non-exclusion probability (parent pair): 0.0758

Average non-exclusion probability (identity): 0.0224

Average non-exclusion probability (sib identity): 0.3122

**** Locus 3139/3140 ****

Allele Count Heterozygotes Homozygotes Frequency

256 1 1 0 0.0313

257 1 1 0 0.0313

263 1 1 0 0.0313

265 2 2 0 0.0625

266 1 1 0 0.0313

267 5 5 0 0.1563

268 2 2 0 0.0625

269 1 1 0 0.0313

270 2 2 0 0.0625

272 1 1 0 0.0313

273 2 2 0 0.0625

274 4 4 0 0.1250

275 3 3 0 0.0938

277 2 2 0 0.0625

279 1 1 0 0.0313

280 1 1 0 0.0313

281 1 1 0 0.0313

282 1 1 0 0.0313

Number of individuals typed: 16

Heterozygotes: 16

Homozygotes: 0

Number of alleles: 18

Observed heterozygosity: 1.0000

Expected heterozygosity: 0.9516

Polymorphic information content (PIC): 0.9168

Average non-exclusion probability (first parent): 0.2708

Average non-exclusion probability (second parent): 0.1568

Average non-exclusion probability (parent pair): 0.0402

Average non-exclusion probability (identity): 0.0112

Average non-exclusion probability (sib identity): 0.2919

**** Locus 3163/3164 ****

Allele Count Heterozygotes Homozygotes Frequency

208 1 1 0 0.0313

209 4 2 1 0.1250

211 1 1 0 0.0313

213 2 2 0 0.0625

216 2 2 0 0.0625

219 1 1 0 0.0313

220 3 3 0 0.0938

222 1 1 0 0.0313

232 1 1 0 0.0313

240 1 1 0 0.0313

242 2 0 1 0.0625

244 1 1 0 0.0313

251 2 2 0 0.0625

256 1 1 0 0.0313

258 2 0 1 0.0625

267 4 2 1 0.1250

274 1 1 0 0.0313

277 1 1 0 0.0313

293 1 1 0 0.0313

Number of individuals typed: 16

Heterozygotes: 12

Homozygotes: 4

Number of alleles: 19

Observed heterozygosity: 0.7500

Expected heterozygosity: 0.9597

Polymorphic information content (PIC): 0.9254

Average non-exclusion probability (first parent): 0.2482

Average non-exclusion probability (second parent): 0.1418

Average non-exclusion probability (parent pair): 0.0336

Average non-exclusion probability (identity): 0.0092

Average non-exclusion probability (sib identity): 0.2875

**** Locus 3181/3182 ****

Allele Count Heterozygotes Homozygotes Frequency

292 3 3 0 0.0938

294 5 5 0 0.1563

296 4 4 0 0.1250

298 3 3 0 0.0938

299 1 1 0 0.0313

310 4 4 0 0.1250

313 4 4 0 0.1250

320 4 4 0 0.1250

322 4 4 0 0.1250

Number of individuals typed: 16

Heterozygotes: 16

Homozygotes: 0

Number of alleles: 9

Observed heterozygosity: 1.0000

Expected heterozygosity: 0.9073

Polymorphic information content (PIC): 0.8662

Average non-exclusion probability (first parent): 0.3999

Average non-exclusion probability (second parent): 0.2475

Average non-exclusion probability (parent pair): 0.0953

Average non-exclusion probability (identity): 0.0274

Average non-exclusion probability (sib identity): 0.3174

**** Locus 3205/3206 ****

Allele Count Heterozygotes Homozygotes Frequency

243 1 1 0 0.0313

244 1 1 0 0.0313

246 1 1 0 0.0313

247 1 1 0 0.0313

249 4 4 0 0.1250

250 1 1 0 0.0313

255 7 7 0 0.2188

256 2 2 0 0.0625

258 2 2 0 0.0625

259 1 1 0 0.0313

260 2 2 0 0.0625

261 2 2 0 0.0625

263 2 2 0 0.0625

264 2 2 0 0.0625

268 1 1 0 0.0313

270 2 2 0 0.0625

Number of individuals typed: 16

Heterozygotes: 16

Homozygotes: 0

Number of alleles: 16

Observed heterozygosity: 1.0000

Expected heterozygosity: 0.9315

Polymorphic information content (PIC): 0.8955

Average non-exclusion probability (first parent): 0.3221

Average non-exclusion probability (second parent): 0.1922

Average non-exclusion probability (parent pair): 0.0566

Average non-exclusion probability (identity): 0.0164

Average non-exclusion probability (sib identity): 0.3029

**** Locus 3245/3246 ****

Allele Count Heterozygotes Homozygotes Frequency

102 3 3 0 0.1000

104 1 1 0 0.0333

106 1 1 0 0.0333

109 4 0 2 0.1333

111 2 2 0 0.0667

113 2 2 0 0.0667

115 1 1 0 0.0333

119 1 1 0 0.0333

121 1 1 0 0.0333

194 1 1 0 0.0333

195 2 2 0 0.0667

198 1 1 0 0.0333

202 1 1 0 0.0333

204 2 2 0 0.0667

205 1 1 0 0.0333

210 2 0 1 0.0667

213 1 1 0 0.0333

219 2 2 0 0.0667

233 1 1 0 0.0333

Number of individuals typed: 15

Heterozygotes: 12

Homozygotes: 3

Number of alleles: 19

Observed heterozygosity: 0.8000

Expected heterozygosity: 0.9655

Polymorphic information content (PIC): 0.9294

Average non-exclusion probability (first parent): 0.2372

Average non-exclusion probability (second parent): 0.1346

Average non-exclusion probability (parent pair): 0.0306

Average non-exclusion probability (identity): 0.0083

Average non-exclusion probability (sib identity): 0.2854

**** Locus 3253/3254 ****

Allele Count Heterozygotes Homozygotes Frequency

289 1 1 0 0.0313

297 2 2 0 0.0625

299 1 1 0 0.0313

301 1 1 0 0.0313

306 3 3 0 0.0938

307 5 1 2 0.1563

309 2 0 1 0.0625

311 1 1 0 0.0313

313 9 1 4 0.2813

315 1 1 0 0.0313

317 1 1 0 0.0313

320 1 1 0 0.0313

322 1 1 0 0.0313

325 1 1 0 0.0313

362 1 1 0 0.0313

378 1 1 0 0.0313

Number of individuals typed: 16

Heterozygotes: 9

Homozygotes: 7

Number of alleles: 16

Observed heterozygosity: 0.5625

Expected heterozygosity: 0.8972

Polymorphic information content (PIC): 0.8590

Average non-exclusion probability (first parent): 0.3993

Average non-exclusion probability (second parent): 0.2490

Average non-exclusion probability (parent pair): 0.0875

Average non-exclusion probability (identity): 0.0273

Average non-exclusion probability (sib identity): 0.3222

**** Locus 3319/3320 ****

Allele Count Heterozygotes Homozygotes Frequency

240 2 2 0 0.0625

248 2 2 0 0.0625

253 3 3 0 0.0938

285 1 1 0 0.0313

299 1 1 0 0.0313

300 1 1 0 0.0313

311 2 2 0 0.0625

314 1 1 0 0.0313

315 2 2 0 0.0625

320 1 1 0 0.0313

321 1 1 0 0.0313

323 1 1 0 0.0313

325 5 5 0 0.1563

330 1 1 0 0.0313

334 1 1 0 0.0313

336 1 1 0 0.0313

340 1 1 0 0.0313

347 1 1 0 0.0313

348 2 2 0 0.0625

350 1 1 0 0.0313

351 1 1 0 0.0313

Number of individuals typed: 16

Heterozygotes: 16

Homozygotes: 0

Number of alleles: 21

Observed heterozygosity: 1.0000

Expected heterozygosity: 0.9637

Polymorphic information content (PIC): 0.9299

Average non-exclusion probability (first parent): 0.2339

Average non-exclusion probability (second parent): 0.1329

Average non-exclusion probability (parent pair): 0.0293

Average non-exclusion probability (identity): 0.0081

Average non-exclusion probability (sib identity): 0.2852

**** Locus 3331/3332 ****

Allele Count Heterozygotes Homozygotes Frequency

138 1 1 0 0.0313

140 2 2 0 0.0625

142 2 2 0 0.0625

143 2 2 0 0.0625

147 1 1 0 0.0313

150 1 1 0 0.0313

151 1 1 0 0.0313

160 2 2 0 0.0625

161 2 2 0 0.0625

163 1 1 0 0.0313

164 2 2 0 0.0625

165 5 5 0 0.1563

166 5 3 1 0.1563

167 2 2 0 0.0625

169 3 3 0 0.0938

Number of individuals typed: 16

Heterozygotes: 15

Homozygotes: 1

Number of alleles: 15

Observed heterozygosity: 0.9375

Expected heterozygosity: 0.9395

Polymorphic information content (PIC): 0.9035

Average non-exclusion probability (first parent): 0.3061

Average non-exclusion probability (second parent): 0.1806

Average non-exclusion probability (parent pair): 0.0523

Average non-exclusion probability (identity): 0.0148

Average non-exclusion probability (sib identity): 0.2986

**** Locus 3343/3344 ****

Allele Count Heterozygotes Homozygotes Frequency

265 1 1 0 0.0313

278 1 1 0 0.0313

303 2 2 0 0.0625

310 5 3 1 0.1563

315 1 1 0 0.0313

316 2 2 0 0.0625

319 1 1 0 0.0313

334 6 6 0 0.1875

346 3 3 0 0.0938

353 1 1 0 0.0313

363 1 1 0 0.0313

391 1 1 0 0.0313

392 1 1 0 0.0313

398 1 1 0 0.0313

412 5 1 2 0.1563

Number of individuals typed: 16

Heterozygotes: 13

Homozygotes: 3

Number of alleles: 15

Observed heterozygosity: 0.8125

Expected heterozygosity: 0.9194

Polymorphic information content (PIC): 0.8812

Average non-exclusion probability (first parent): 0.3580

Average non-exclusion probability (second parent): 0.2179

Average non-exclusion probability (parent pair): 0.0731

Average non-exclusion probability (identity): 0.0214

Average non-exclusion probability (sib identity): 0.3100

**** Locus 3351/3352 ****

Allele Count Heterozygotes Homozygotes Frequency

144 5 5 0 0.1563

145 2 2 0 0.0625

149 2 0 1 0.0625

150 4 2 1 0.1250

151 1 1 0 0.0313

152 2 2 0 0.0625

153 4 4 0 0.1250

154 1 1 0 0.0313

156 2 0 1 0.0625

158 4 4 0 0.1250

159 1 1 0 0.0313

162 1 1 0 0.0313

169 2 2 0 0.0625

174 1 1 0 0.0313

Number of individuals typed: 16

Heterozygotes: 13

Homozygotes: 3

Number of alleles: 14

Observed heterozygosity: 0.8125

Expected heterozygosity: 0.9335

Polymorphic information content (PIC): 0.8965

Average non-exclusion probability (first parent): 0.3245

Average non-exclusion probability (second parent): 0.1933

Average non-exclusion probability (parent pair): 0.0598

Average non-exclusion probability (identity): 0.0169

Average non-exclusion probability (sib identity): 0.3021

**** Locus 3353/3354 ****

Allele Count Heterozygotes Homozygotes Frequency

102 3 3 0 0.1000

104 1 1 0 0.0333

106 1 1 0 0.0333

109 9 5 2 0.3000

111 3 3 0 0.1000

113 3 3 0 0.1000

115 1 1 0 0.0333

116 3 3 0 0.1000

119 1 1 0 0.0333

121 2 2 0 0.0667

122 2 2 0 0.0667

125 1 1 0 0.0333

Number of individuals typed: 15

Heterozygotes: 13

Homozygotes: 2

Number of alleles: 12

Observed heterozygosity: 0.8667

Expected heterozygosity: 0.8851

Polymorphic information content (PIC): 0.8432

Average non-exclusion probability (first parent): 0.4346

Average non-exclusion probability (second parent): 0.2754

Average non-exclusion probability (parent pair): 0.1061

Average non-exclusion probability (identity): 0.0332

Average non-exclusion probability (sib identity): 0.3305

**** Locus 3355/3356 ****

Allele Count Heterozygotes Homozygotes Frequency

283 3 3 0 0.0938

285 5 5 0 0.1563

286 2 2 0 0.0625

288 5 5 0 0.1563

290 11 11 0 0.3438

292 3 3 0 0.0938

293 1 1 0 0.0313

295 1 1 0 0.0313

296 1 1 0 0.0313

Number of individuals typed: 16

Heterozygotes: 16

Homozygotes: 0

Number of alleles: 9

Observed heterozygosity: 1.0000

Expected heterozygosity: 0.8347

Polymorphic information content (PIC): 0.7873

Average non-exclusion probability (first parent): 0.5374

Average non-exclusion probability (second parent): 0.3614

Average non-exclusion probability (parent pair): 0.1749

Average non-exclusion probability (identity): 0.0579

Average non-exclusion probability (sib identity): 0.3602

**** Locus 5211-B07 ****

Allele Count Heterozygotes Homozygotes Frequency

240 5 3 1 0.1563

244 2 2 0 0.0625

246 9 1 4 0.2813

247 4 2 1 0.1250

249 6 4 1 0.1875

252 4 4 0 0.1250

253 2 2 0 0.0625

Number of individuals typed: 16

Heterozygotes: 9

Homozygotes: 7

Number of alleles: 7

Observed heterozygosity: 0.5625

Expected heterozygosity: 0.8488

Polymorphic information content (PIC): 0.7993

Average non-exclusion probability (first parent): 0.5254

Average non-exclusion probability (second parent): 0.3511

Average non-exclusion probability (parent pair): 0.1731

Average non-exclusion probability (identity): 0.0546

Average non-exclusion probability (sib identity): 0.3525

**** Locus 535/536 ****

Allele Count Heterozygotes Homozygotes Frequency

282 1 1 0 0.0333

288 2 0 1 0.0667

289 4 2 1 0.1333

290 18 2 8 0.6000

292 2 0 1 0.0667

293 1 1 0 0.0333

296 1 1 0 0.0333

301 1 1 0 0.0333

Number of individuals typed: 15

Heterozygotes: 4

Homozygotes: 11

Number of alleles: 8

Observed heterozygosity: 0.2667

Expected heterozygosity: 0.6299

Polymorphic information content (PIC): 0.5859

Average non-exclusion probability (first parent): 0.7719

Average non-exclusion probability (second parent): 0.5855

Average non-exclusion probability (parent pair): 0.3735

Average non-exclusion probability (identity): 0.1760

Average non-exclusion probability (sib identity): 0.4895

**** Locus 541/542 ****

Allele Count Heterozygotes Homozygotes Frequency

214 1 1 0 0.0313

216 1 1 0 0.0313

217 3 3 0 0.0938

218 1 1 0 0.0313

221 1 1 0 0.0313

224 3 3 0 0.0938

226 3 3 0 0.0938

229 1 1 0 0.0313

232 1 1 0 0.0313

237 2 0 1 0.0625

238 2 2 0 0.0625

240 1 1 0 0.0313

242 1 1 0 0.0313

246 1 1 0 0.0313

251 1 1 0 0.0313

252 2 2 0 0.0625

255 3 1 1 0.0938

256 2 2 0 0.0625

258 1 1 0 0.0313

264 1 1 0 0.0313

Number of individuals typed: 16

Heterozygotes: 14

Homozygotes: 2

Number of alleles: 20

Observed heterozygosity: 0.8750

Expected heterozygosity: 0.9677

Polymorphic information content (PIC): 0.9340

Average non-exclusion probability (first parent): 0.2248

Average non-exclusion probability (second parent): 0.1266

Average non-exclusion probability (parent pair): 0.0275

Average non-exclusion probability (identity): 0.0074

Average non-exclusion probability (sib identity): 0.2831

**** Locus 615/616 ****

Allele Count Heterozygotes Homozygotes Frequency

224 1 1 0 0.0313

226 3 1 1 0.0938

227 8 2 3 0.2500

228 2 2 0 0.0625

231 1 1 0 0.0313

232 6 0 3 0.1875

233 2 2 0 0.0625

235 1 1 0 0.0313

236 2 0 1 0.0625

238 3 1 1 0.0938

240 3 1 1 0.0938

Number of individuals typed: 16

Heterozygotes: 6

Homozygotes: 10

Number of alleles: 11

Observed heterozygosity: 0.3750

Expected heterozygosity: 0.8891

Polymorphic information content (PIC): 0.8475

Average non-exclusion probability (first parent): 0.4304

Average non-exclusion probability (second parent): 0.2725

Average non-exclusion probability (parent pair): 0.1083

Average non-exclusion probability (identity): 0.0330

Average non-exclusion probability (sib identity): 0.3276

**** Locus 631/632 ****

Allele Count Heterozygotes Homozygotes Frequency

147 1 1 0 0.0313

153 1 1 0 0.0313

156 2 0 1 0.0625

158 2 2 0 0.0625

159 1 1 0 0.0313

168 1 1 0 0.0313

176 12 0 6 0.3750

177 2 0 1 0.0625

178 2 0 1 0.0625

179 7 1 3 0.2188

180 1 1 0 0.0313

Number of individuals typed: 16

Heterozygotes: 4

Homozygotes: 12

Number of alleles: 11

Observed heterozygosity: 0.2500

Expected heterozygosity: 0.8165

Polymorphic information content (PIC): 0.7695

Average non-exclusion probability (first parent): 0.5577

Average non-exclusion probability (second parent): 0.3802

Average non-exclusion probability (parent pair): 0.1854

Average non-exclusion probability (identity): 0.0652

Average non-exclusion probability (sib identity): 0.3708

**** Locus 687/688 ****

Allele Count Heterozygotes Homozygotes Frequency

260 1 1 0 0.0333

265 1 1 0 0.0333

270 3 3 0 0.1000

271 3 3 0 0.1000

272 2 2 0 0.0667

275 1 1 0 0.0333

276 1 1 0 0.0333

279 1 1 0 0.0333

282 2 2 0 0.0667

283 2 2 0 0.0667

285 2 2 0 0.0667

287 4 4 0 0.1333

288 3 3 0 0.1000

292 2 2 0 0.0667

297 1 1 0 0.0333

305 1 1 0 0.0333

Number of individuals typed: 15

Heterozygotes: 15

Homozygotes: 0

Number of alleles: 16

Observed heterozygosity: 1.0000

Expected heterozygosity: 0.9540

Polymorphic information content (PIC): 0.9169

Average non-exclusion probability (first parent): 0.2727

Average non-exclusion probability (second parent): 0.1577

Average non-exclusion probability (parent pair): 0.0413

Average non-exclusion probability (identity): 0.0114

Average non-exclusion probability (sib identity): 0.2917

**** Locus NFSG-009 ****

Allele Count Heterozygotes Homozygotes Frequency

237 1 1 0 0.0313

239 1 1 0 0.0313

243 4 2 1 0.1250

244 6 6 0 0.1875

245 2 2 0 0.0625

248 2 2 0 0.0625

249 5 5 0 0.1563

250 2 2 0 0.0625

254 2 2 0 0.0625

263 1 1 0 0.0313

265 3 1 1 0.0938

266 1 1 0 0.0313

271 2 2 0 0.0625

Number of individuals typed: 16

Heterozygotes: 14

Homozygotes: 2

Number of alleles: 13

Observed heterozygosity: 0.8750

Expected heterozygosity: 0.9214

Polymorphic information content (PIC): 0.8833

Average non-exclusion probability (first parent): 0.3552

Average non-exclusion probability (second parent): 0.2154

Average non-exclusion probability (parent pair): 0.0721

Average non-exclusion probability (identity): 0.0208

Average non-exclusion probability (sib identity): 0.3089

**** Locus NFSG-035 ****

Allele Count Heterozygotes Homozygotes Frequency

117 3 3 0 0.1000

119 2 2 0 0.0667

121 3 3 0 0.1000

122 1 1 0 0.0333

124 2 2 0 0.0667

125 3 3 0 0.1000

126 1 1 0 0.0333

130 5 1 2 0.1667

131 3 3 0 0.1000

134 3 3 0 0.1000

140 1 1 0 0.0333

141 2 0 1 0.0667

151 1 1 0 0.0333

Number of individuals typed: 15

Heterozygotes: 12

Homozygotes: 3

Number of alleles: 13

Observed heterozygosity: 0.8000

Expected heterozygosity: 0.9356

Polymorphic information content (PIC): 0.8966

Average non-exclusion probability (first parent): 0.3253

Average non-exclusion probability (second parent): 0.1935

Average non-exclusion probability (parent pair): 0.0601

Average non-exclusion probability (identity): 0.0169

Average non-exclusion probability (sib identity): 0.3020

**** Locus NFSG-036 ****

Allele Count Heterozygotes Homozygotes Frequency

120 3 3 0 0.0938

122 1 1 0 0.0313

125 1 1 0 0.0313

127 3 3 0 0.0938

129 1 1 0 0.0313

132 3 3 0 0.0938

134 1 1 0 0.0313

136 5 5 0 0.1563

137 1 1 0 0.0313

138 2 0 1 0.0625

142 4 4 0 0.1250

148 1 1 0 0.0313

151 1 1 0 0.0313

153 1 1 0 0.0313

154 2 2 0 0.0625

165 1 1 0 0.0313

167 1 1 0 0.0313

Number of individuals typed: 16

Heterozygotes: 15

Homozygotes: 1

Number of alleles: 17

Observed heterozygosity: 0.9375

Expected heterozygosity: 0.9456

Polymorphic information content (PIC): 0.9101

Average non-exclusion probability (first parent): 0.2890

Average non-exclusion probability (second parent): 0.1690

Average non-exclusion probability (parent pair): 0.0464

Average non-exclusion probability (identity): 0.0130

Average non-exclusion probability (sib identity): 0.2952

**** Locus NFSG-065 ****

Allele Count Heterozygotes Homozygotes Frequency

223 1 1 0 0.0313

224 1 1 0 0.0313

225 12 8 2 0.3750

227 5 5 0 0.1563

229 1 1 0 0.0313

230 3 1 1 0.0938

234 7 5 1 0.2188

235 1 1 0 0.0313

236 1 1 0 0.0313

Number of individuals typed: 16

Heterozygotes: 12

Homozygotes: 4

Number of alleles: 9

Observed heterozygosity: 0.7500

Expected heterozygosity: 0.7984

Polymorphic information content (PIC): 0.7449

Average non-exclusion probability (first parent): 0.5998

Average non-exclusion probability (second parent): 0.4201

Average non-exclusion probability (parent pair): 0.2280

Average non-exclusion probability (identity): 0.0799

Average non-exclusion probability (sib identity): 0.3833

**** Locus NFSG-112 ****

Allele Count Heterozygotes Homozygotes Frequency

189 21 11 5 0.6563

190 4 4 0 0.1250

195 7 7 0 0.2188

Number of individuals typed: 16

Heterozygotes: 11

Homozygotes: 5

Number of alleles: 3

Observed heterozygosity: 0.6875

Expected heterozygosity: 0.5222

Polymorphic information content (PIC): 0.4497

Average non-exclusion probability (first parent): 0.8721

Average non-exclusion probability (second parent): 0.7349

Average non-exclusion probability (parent pair): 0.5899

Average non-exclusion probability (identity): 0.3003

Average non-exclusion probability (sib identity): 0.5722

**** Locus NFSG-125 ****

Allele Count Heterozygotes Homozygotes Frequency

203 23 3 10 0.7188

206 4 2 1 0.1250

208 1 1 0 0.0313

214 3 3 0 0.0938

220 1 1 0 0.0313

Number of individuals typed: 16

Heterozygotes: 5

Homozygotes: 11

Number of alleles: 5

Observed heterozygosity: 0.3125

Expected heterozygosity: 0.4718

Polymorphic information content (PIC): 0.4294

Average non-exclusion probability (first parent): 0.8873

Average non-exclusion probability (second parent): 0.7331

Average non-exclusion probability (parent pair): 0.5656

Average non-exclusion probability (identity): 0.3224

Average non-exclusion probability (sib identity): 0.6021

**** Locus NFSG-133 ****

Allele Count Heterozygotes Homozygotes Frequency

148 1 1 0 0.0357

149 1 1 0 0.0357

150 1 1 0 0.0357

151 3 3 0 0.1071

152 2 2 0 0.0714

154 4 4 0 0.1429

156 7 3 2 0.2500

158 3 3 0 0.1071

159 2 0 1 0.0714

161 1 1 0 0.0357

167 3 1 1 0.1071

Number of individuals typed: 14

Heterozygotes: 10

Homozygotes: 4

Number of alleles: 11

Observed heterozygosity: 0.7143

Expected heterozygosity: 0.8995

Polymorphic information content (PIC): 0.8545

Average non-exclusion probability (first parent): 0.4172

Average non-exclusion probability (second parent): 0.2618

Average non-exclusion probability (parent pair): 0.1011

Average non-exclusion probability (identity): 0.0304

Average non-exclusion probability (sib identity): 0.3239

**** Locus NFSG-134 ****

Allele Count Heterozygotes Homozygotes Frequency

116 9 3 3 0.2813

118 1 1 0 0.0313

119 2 0 1 0.0625

120 4 2 1 0.1250

122 1 1 0 0.0313

123 11 1 5 0.3438

128 1 1 0 0.0313

130 1 1 0 0.0313

132 1 1 0 0.0313

134 1 1 0 0.0313

Number of individuals typed: 16

Heterozygotes: 6

Homozygotes: 10

Number of alleles: 10

Observed heterozygosity: 0.3750

Expected heterozygosity: 0.8024

Polymorphic information content (PIC): 0.7483

Average non-exclusion probability (first parent): 0.5919

Average non-exclusion probability (second parent): 0.4144

Average non-exclusion probability (parent pair): 0.2227

Average non-exclusion probability (identity): 0.0787

Average non-exclusion probability (sib identity): 0.3810

**** Locus NFSG-137 ****

Allele Count Heterozygotes Homozygotes Frequency

119 2 0 1 0.0625

120 1 1 0 0.0313

122 1 1 0 0.0313

124 7 3 2 0.2188

126 6 2 2 0.1875

127 8 0 4 0.2500

128 2 2 0 0.0625

129 2 0 1 0.0625

130 3 1 1 0.0938

Number of individuals typed: 16

Heterozygotes: 5

Homozygotes: 11

Number of alleles: 9

Observed heterozygosity: 0.3125

Expected heterozygosity: 0.8589

Polymorphic information content (PIC): 0.8114

Average non-exclusion probability (first parent): 0.5009

Average non-exclusion probability (second parent): 0.3308

Average non-exclusion probability (parent pair): 0.1551

Average non-exclusion probability (identity): 0.0489

Average non-exclusion probability (sib identity): 0.3462

**** Locus NFSG-139 ****

Allele Count Heterozygotes Homozygotes Frequency

178 3 3 0 0.0938

182 3 3 0 0.0938

183 1 1 0 0.0313

184 20 4 8 0.6250

185 1 1 0 0.0313

194 3 3 0 0.0938

196 1 1 0 0.0313

Number of individuals typed: 16

Heterozygotes: 8

Homozygotes: 8

Number of alleles: 7

Observed heterozygosity: 0.5000

Expected heterozygosity: 0.5988

Polymorphic information content (PIC): 0.5566

Average non-exclusion probability (first parent): 0.7987

Average non-exclusion probability (second parent): 0.6155

Average non-exclusion probability (parent pair): 0.4093

Average non-exclusion probability (identity): 0.1998

Average non-exclusion probability (sib identity): 0.5099

**** Locus NFSG-200 ****

Allele Count Heterozygotes Homozygotes Frequency

107 1 1 0 0.0313

109 5 5 0 0.1563

110 2 2 0 0.0625

112 2 2 0 0.0625

113 2 2 0 0.0625

114 1 1 0 0.0313

119 3 3 0 0.0938

120 1 1 0 0.0313

121 7 3 2 0.2188

123 2 2 0 0.0625

124 1 1 0 0.0313

127 1 1 0 0.0313

132 2 2 0 0.0625

133 1 1 0 0.0313

146 1 1 0 0.0313

Number of individuals typed: 16

Heterozygotes: 14

Homozygotes: 2

Number of alleles: 15

Observed heterozygosity: 0.8750

Expected heterozygosity: 0.9214

Polymorphic information content (PIC): 0.8841

Average non-exclusion probability (first parent): 0.3496

Average non-exclusion probability (second parent): 0.2118

Average non-exclusion probability (parent pair): 0.0682

Average non-exclusion probability (identity): 0.0200

Average non-exclusion probability (sib identity): 0.3087

**** Locus NFSG-202 ****

Allele Count Heterozygotes Homozygotes Frequency

149 11 5 3 0.3667

167 15 9 3 0.5000

170 3 3 0 0.1000

171 1 1 0 0.0333

Number of individuals typed: 15

Heterozygotes: 9

Homozygotes: 6

Number of alleles: 4

Observed heterozygosity: 0.6000

Expected heterozygosity: 0.6253

Polymorphic information content (PIC): 0.5287

Average non-exclusion probability (first parent): 0.8100

Average non-exclusion probability (second parent): 0.6730

Average non-exclusion probability (parent pair): 0.5184

Average non-exclusion probability (identity): 0.2323

Average non-exclusion probability (sib identity): 0.5058

**** Locus NFSG-219 ****

Allele Count Heterozygotes Homozygotes Frequency

165 1 1 0 0.0313

168 1 1 0 0.0313

173 1 1 0 0.0313

176 4 4 0 0.1250

177 1 1 0 0.0313

181 1 1 0 0.0313

183 1 1 0 0.0313

184 3 3 0 0.0938

185 2 2 0 0.0625

186 1 1 0 0.0313

188 1 1 0 0.0313

189 3 3 0 0.0938

190 2 2 0 0.0625

191 1 1 0 0.0313

193 3 3 0 0.0938

195 1 1 0 0.0313

196 1 1 0 0.0313

198 2 2 0 0.0625

199 2 0 1 0.0625

Number of individuals typed: 16

Heterozygotes: 15

Homozygotes: 1

Number of alleles: 19

Observed heterozygosity: 0.9375

Expected heterozygosity: 0.9617

Polymorphic information content (PIC): 0.9275

Average non-exclusion probability (first parent): 0.2428

Average non-exclusion probability (second parent): 0.1382

Average non-exclusion probability (parent pair): 0.0322

Average non-exclusion probability (identity): 0.0088

Average non-exclusion probability (sib identity): 0.2864

**** Locus NFSG-246 ****

Allele Count Heterozygotes Homozygotes Frequency

120 1 1 0 0.0313

130 8 4 2 0.2500

132 4 4 0 0.1250

134 15 11 2 0.4688

136 3 3 0 0.0938

167 1 1 0 0.0313

Number of individuals typed: 16

Heterozygotes: 12

Homozygotes: 4

Number of alleles: 6

Observed heterozygosity: 0.7500

Expected heterozygosity: 0.7137

Polymorphic information content (PIC): 0.6487

Average non-exclusion probability (first parent): 0.7156

Average non-exclusion probability (second parent): 0.5397

Average non-exclusion probability (parent pair): 0.3496

Average non-exclusion probability (identity): 0.1380

Average non-exclusion probability (sib identity): 0.4388

**** Locus PV1115/1116 ****

Allele Count Heterozygotes Homozygotes Frequency

139 4 0 2 0.1250

144 2 2 0 0.0625

146 1 1 0 0.0313

147 4 0 2 0.1250

148 2 2 0 0.0625

149 1 1 0 0.0313

150 2 2 0 0.0625

151 2 2 0 0.0625

152 1 1 0 0.0313

153 1 1 0 0.0313

154 5 1 2 0.1563

155 2 0 1 0.0625

157 2 2 0 0.0625

158 1 1 0 0.0313

160 1 1 0 0.0313

161 1 1 0 0.0313

Number of individuals typed: 16

Heterozygotes: 9

Homozygotes: 7

Number of alleles: 16

Observed heterozygosity: 0.5625

Expected heterozygosity: 0.9435

Polymorphic information content (PIC): 0.9079

Average non-exclusion probability (first parent): 0.2949

Average non-exclusion probability (second parent): 0.1729

Average non-exclusion probability (parent pair): 0.0484

Average non-exclusion probability (identity): 0.0136

Average non-exclusion probability (sib identity): 0.2964

**** Locus PV1143/1144 ****

Allele Count Heterozygotes Homozygotes Frequency

156 1 1 0 0.0313

159 1 1 0 0.0313

161 1 1 0 0.0313

164 1 1 0 0.0313

167 2 2 0 0.0625

173 1 1 0 0.0313

174 3 3 0 0.0938

177 1 1 0 0.0313

178 1 1 0 0.0313

179 3 3 0 0.0938

180 5 5 0 0.1563

181 2 2 0 0.0625

182 1 1 0 0.0313

183 1 1 0 0.0313

184 1 1 0 0.0313

185 2 2 0 0.0625

187 2 2 0 0.0625

195 1 1 0 0.0313

212 1 1 0 0.0313

221 1 1 0 0.0313

Number of individuals typed: 16

Heterozygotes: 16

Homozygotes: 0

Number of alleles: 20

Observed heterozygosity: 1.0000

Expected heterozygosity: 0.9597

Polymorphic information content (PIC): 0.9256

Average non-exclusion probability (first parent): 0.2465

Average non-exclusion probability (second parent): 0.1409

Average non-exclusion probability (parent pair): 0.0328

Average non-exclusion probability (identity): 0.0091

Average non-exclusion probability (sib identity): 0.2874

**** Locus PV1197/1198 ****

Allele Count Heterozygotes Homozygotes Frequency

263 4 4 0 0.1250

285 4 4 0 0.1250

291 1 1 0 0.0313

300 1 1 0 0.0313

305 1 1 0 0.0313

313 3 3 0 0.0938

316 2 2 0 0.0625

317 1 1 0 0.0313

318 1 1 0 0.0313

320 2 2 0 0.0625

322 1 1 0 0.0313

323 3 3 0 0.0938

324 1 1 0 0.0313

325 4 4 0 0.1250

336 1 1 0 0.0313

337 1 1 0 0.0313

351 1 1 0 0.0313

Number of individuals typed: 16

Heterozygotes: 16

Homozygotes: 0

Number of alleles: 17

Observed heterozygosity: 1.0000

Expected heterozygosity: 0.9476

Polymorphic information content (PIC): 0.9122

Average non-exclusion probability (first parent): 0.2842

Average non-exclusion probability (second parent): 0.1656

Average non-exclusion probability (parent pair): 0.0450

Average non-exclusion probability (identity): 0.0125

Average non-exclusion probability (sib identity): 0.2941

**** Locus PV1729/1730 ****

Allele Count Heterozygotes Homozygotes Frequency

265 1 1 0 0.0313

266 2 2 0 0.0625

267 2 2 0 0.0625

268 1 1 0 0.0313

270 3 3 0 0.0938

271 1 1 0 0.0313

272 3 3 0 0.0938

273 5 5 0 0.1563

274 2 2 0 0.0625

276 2 2 0 0.0625

277 1 1 0 0.0313

279 1 1 0 0.0313

281 1 1 0 0.0313

282 1 1 0 0.0313

284 1 1 0 0.0313

285 3 3 0 0.0938

286 1 1 0 0.0313

301 1 1 0 0.0313

Number of individuals typed: 16

Heterozygotes: 16

Homozygotes: 0

Number of alleles: 18

Observed heterozygosity: 1.0000

Expected heterozygosity: 0.9536

Polymorphic information content (PIC): 0.9189

Average non-exclusion probability (first parent): 0.2655

Average non-exclusion probability (second parent): 0.1532

Average non-exclusion probability (parent pair): 0.0386

Average non-exclusion probability (identity): 0.0107

Average non-exclusion probability (sib identity): 0.2908

**** Locus PVCA-145/146 ****

Allele Count Heterozygotes Homozygotes Frequency

304 31 1 15 0.9688

334 1 1 0 0.0313

Number of individuals typed: 16

Heterozygotes: 1

Homozygotes: 15

Number of alleles: 2

Observed heterozygosity: 0.0625

Expected heterozygosity: 0.0625

Polymorphic information content (PIC): 0.0587

Average non-exclusion probability (first parent): 0.9982

Average non-exclusion probability (second parent): 0.9706

Average non-exclusion probability (parent pair): 0.9439

Average non-exclusion probability (identity): 0.8844

Average non-exclusion probability (sib identity): 0.9408

**** Locus PVCA-17/18 ****

Allele Count Heterozygotes Homozygotes Frequency

335 2 2 0 0.0625

338 1 1 0 0.0313

341 1 1 0 0.0313

342 1 1 0 0.0313

344 1 1 0 0.0313

346 4 4 0 0.1250

351 5 5 0 0.1563

360 4 4 0 0.1250

362 2 2 0 0.0625

365 2 2 0 0.0625

368 1 1 0 0.0313

369 1 1 0 0.0313

370 1 1 0 0.0313

374 1 1 0 0.0313

378 1 1 0 0.0313

382 1 1 0 0.0313

385 1 1 0 0.0313

391 2 2 0 0.0625

Number of individuals typed: 16

Heterozygotes: 16

Homozygotes: 0

Number of alleles: 18

Observed heterozygosity: 1.0000

Expected heterozygosity: 0.9476

Polymorphic information content (PIC): 0.9124

Average non-exclusion probability (first parent): 0.2820

Average non-exclusion probability (second parent): 0.1644

Average non-exclusion probability (parent pair): 0.0438

Average non-exclusion probability (identity): 0.0123

Average non-exclusion probability (sib identity): 0.2941

**** Locus PVCA-173/174 ****

Allele Count Heterozygotes Homozygotes Frequency

241 2 2 0 0.0625

250 2 2 0 0.0625

304 1 1 0 0.0313

305 1 1 0 0.0313

306 1 1 0 0.0313

309 4 4 0 0.1250

310 4 4 0 0.1250

311 1 1 0 0.0313

312 2 2 0 0.0625

313 2 2 0 0.0625

314 1 1 0 0.0313

315 2 2 0 0.0625

318 1 1 0 0.0313

319 1 1 0 0.0313

320 2 2 0 0.0625

332 2 2 0 0.0625

335 1 1 0 0.0313

342 1 1 0 0.0313

356 1 1 0 0.0313

Number of individuals typed: 16

Heterozygotes: 16

Homozygotes: 0

Number of alleles: 19

Observed heterozygosity: 1.0000

Expected heterozygosity: 0.9617

Polymorphic information content (PIC): 0.9276

Average non-exclusion probability (first parent): 0.2422

Average non-exclusion probability (second parent): 0.1379

Average non-exclusion probability (parent pair): 0.0320

Average non-exclusion probability (identity): 0.0087

Average non-exclusion probability (sib identity): 0.2864

**** Locus PVCA-19/20 ****

Allele Count Heterozygotes Homozygotes Frequency

326 6 2 2 0.1875

327 2 2 0 0.0625

328 1 1 0 0.0313

331 2 2 0 0.0625

332 2 2 0 0.0625

335 1 1 0 0.0313

341 1 1 0 0.0313

342 1 1 0 0.0313

344 2 2 0 0.0625

345 1 1 0 0.0313

346 2 2 0 0.0625

347 1 1 0 0.0313

348 1 1 0 0.0313

349 1 1 0 0.0313

351 1 1 0 0.0313

353 1 1 0 0.0313

354 1 1 0 0.0313

388 3 3 0 0.0938

389 1 1 0 0.0313

391 1 1 0 0.0313

Number of individuals typed: 16

Heterozygotes: 14

Homozygotes: 2

Number of alleles: 20

Observed heterozygosity: 0.8750

Expected heterozygosity: 0.9536

Polymorphic information content (PIC): 0.9194

Average non-exclusion probability (first parent): 0.2612

Average non-exclusion probability (second parent): 0.1508

Average non-exclusion probability (parent pair): 0.0363

Average non-exclusion probability (identity): 0.0102

Average non-exclusion probability (sib identity): 0.2906

**** Locus PVCA-285/286 ****

Allele Count Heterozygotes Homozygotes Frequency

203 10 8 1 0.3125

205 1 1 0 0.0313

208 1 1 0 0.0313

210 1 1 0 0.0313

211 2 2 0 0.0625

220 5 5 0 0.1563

224 5 5 0 0.1563

226 6 6 0 0.1875

229 1 1 0 0.0313

Number of individuals typed: 16

Heterozygotes: 15

Homozygotes: 1

Number of alleles: 9

Observed heterozygosity: 0.9375

Expected heterozygosity: 0.8367

Polymorphic information content (PIC): 0.7866

Average non-exclusion probability (first parent): 0.5416

Average non-exclusion probability (second parent): 0.3662

Average non-exclusion probability (parent pair): 0.1834

Average non-exclusion probability (identity): 0.0598

Average non-exclusion probability (sib identity): 0.3597

**** Locus PVCA-317/318 ****

Allele Count Heterozygotes Homozygotes Frequency

150 3 3 0 0.0938

152 1 1 0 0.0313

153 8 8 0 0.2500

154 1 1 0 0.0313

155 3 3 0 0.0938

156 2 2 0 0.0625

157 3 3 0 0.0938

158 2 2 0 0.0625

159 6 0 3 0.1875

160 1 1 0 0.0313

162 1 1 0 0.0313

163 1 1 0 0.0313

Number of individuals typed: 16

Heterozygotes: 13

Homozygotes: 3

Number of alleles: 12

Observed heterozygosity: 0.8125

Expected heterozygosity: 0.8911

Polymorphic information content (PIC): 0.8500

Average non-exclusion probability (first parent): 0.4244

Average non-exclusion probability (second parent): 0.2680

Average non-exclusion probability (parent pair): 0.1047

Average non-exclusion probability (identity): 0.0320

Average non-exclusion probability (sib identity): 0.3264

**** Locus PVCA-349/350 ****

Allele Count Heterozygotes Homozygotes Frequency

234 1 1 0 0.0313

235 3 3 0 0.0938

236 3 3 0 0.0938

238 2 2 0 0.0625

241 1 1 0 0.0313

243 4 4 0 0.1250

245 2 2 0 0.0625

246 1 1 0 0.0313

268 1 1 0 0.0313

269 1 1 0 0.0313

270 1 1 0 0.0313

273 1 1 0 0.0313

275 1 1 0 0.0313

293 1 1 0 0.0313

294 1 1 0 0.0313

303 1 1 0 0.0313

306 1 1 0 0.0313

308 1 1 0 0.0313

309 2 2 0 0.0625

316 1 1 0 0.0313

337 1 1 0 0.0313

365 1 1 0 0.0313

Number of individuals typed: 16

Heterozygotes: 16

Homozygotes: 0

Number of alleles: 22

Observed heterozygosity: 1.0000

Expected heterozygosity: 0.9698

Polymorphic information content (PIC): 0.9362

Average non-exclusion probability (first parent): 0.2169

Average non-exclusion probability (second parent): 0.1220

Average non-exclusion probability (parent pair): 0.0253

Average non-exclusion probability (identity): 0.0069

Average non-exclusion probability (sib identity): 0.2820

**** Locus PVCA-415/416 ****

Allele Count Heterozygotes Homozygotes Frequency

137 2 2 0 0.0625

139 1 1 0 0.0313

140 3 1 1 0.0938

141 3 3 0 0.0938

142 1 1 0 0.0313

144 2 2 0 0.0625

146 2 2 0 0.0625

148 1 1 0 0.0313

149 4 4 0 0.1250

151 1 1 0 0.0313

152 1 1 0 0.0313

160 1 1 0 0.0313

161 3 3 0 0.0938

165 1 1 0 0.0313

172 1 1 0 0.0313

195 1 1 0 0.0313

197 1 1 0 0.0313

203 1 1 0 0.0313

205 1 1 0 0.0313

210 1 1 0 0.0313

Number of individuals typed: 16

Heterozygotes: 15

Homozygotes: 1

Number of alleles: 20

Observed heterozygosity: 0.9375

Expected heterozygosity: 0.9637

Polymorphic information content (PIC): 0.9297

Average non-exclusion probability (first parent): 0.2362

Average non-exclusion probability (second parent): 0.1341

Average non-exclusion probability (parent pair): 0.0304

Average non-exclusion probability (identity): 0.0083

Average non-exclusion probability (sib identity): 0.2853

**** Locus PVCA-7/8 ****

Allele Count Heterozygotes Homozygotes Frequency

289 1 1 0 0.0313

291 1 1 0 0.0313

292 2 2 0 0.0625

295 2 2 0 0.0625

296 1 1 0 0.0313

297 1 1 0 0.0313

305 1 1 0 0.0313

306 2 2 0 0.0625

311 1 1 0 0.0313

313 1 1 0 0.0313

314 1 1 0 0.0313

318 1 1 0 0.0313

320 1 1 0 0.0313

323 1 1 0 0.0313

325 3 3 0 0.0938

326 2 2 0 0.0625

328 2 2 0 0.0625

330 1 1 0 0.0313

332 2 2 0 0.0625

333 2 2 0 0.0625

334 2 2 0 0.0625

341 1 1 0 0.0313

Number of individuals typed: 16

Heterozygotes: 16

Homozygotes: 0

Number of alleles: 22

Observed heterozygosity: 1.0000

Expected heterozygosity: 0.9778

Polymorphic information content (PIC): 0.9447

Average non-exclusion probability (first parent): 0.1933

Average non-exclusion probability (second parent): 0.1070

Average non-exclusion probability (parent pair): 0.0201

Average non-exclusion probability (identity): 0.0054

Average non-exclusion probability (sib identity): 0.2777

**** Locus PVCA-815/816 ****

Allele Count Heterozygotes Homozygotes Frequency

302 3 3 0 0.0938

303 9 3 3 0.2813

304 4 0 2 0.1250

305 1 1 0 0.0313

306 4 2 1 0.1250

307 4 2 1 0.1250

308 1 1 0 0.0313

310 1 1 0 0.0313

311 1 1 0 0.0313

312 1 1 0 0.0313

314 2 0 1 0.0625

317 1 1 0 0.0313

Number of individuals typed: 16

Heterozygotes: 8

Homozygotes: 8

Number of alleles: 12

Observed heterozygosity: 0.5000

Expected heterozygosity: 0.8831

Polymorphic information content (PIC): 0.8417

Average non-exclusion probability (first parent): 0.4402

Average non-exclusion probability (second parent): 0.2802

Average non-exclusion probability (parent pair): 0.1121

Average non-exclusion probability (identity): 0.0347

Average non-exclusion probability (sib identity): 0.3309

**** Locus PVCA-893/894 ****

Allele Count Heterozygotes Homozygotes Frequency

297 3 3 0 0.0938

316 7 1 3 0.2188

317 2 2 0 0.0625

318 11 3 4 0.3438

323 2 2 0 0.0625

324 1 1 0 0.0313

325 3 3 0 0.0938

330 1 1 0 0.0313

336 2 2 0 0.0625

Number of individuals typed: 16

Heterozygotes: 9

Homozygotes: 7

Number of alleles: 9

Observed heterozygosity: 0.5625

Expected heterozygosity: 0.8286

Polymorphic information content (PIC): 0.7803

Average non-exclusion probability (first parent): 0.5465

Average non-exclusion probability (second parent): 0.3702

Average non-exclusion probability (parent pair): 0.1818

Average non-exclusion probability (identity): 0.0614

Average non-exclusion probability (sib identity): 0.3640

**** Locus PVCA-979/980 ****

Allele Count Heterozygotes Homozygotes Frequency

283 1 1 0 0.0313

285 16 14 1 0.5000

293 2 2 0 0.0625

295 2 2 0 0.0625

297 3 3 0 0.0938

301 5 5 0 0.1563

303 2 2 0 0.0625

310 1 1 0 0.0313

Number of individuals typed: 16

Heterozygotes: 15

Homozygotes: 1

Number of alleles: 8

Observed heterozygosity: 0.9375

Expected heterozygosity: 0.7258

Polymorphic information content (PIC): 0.6782

Average non-exclusion probability (first parent): 0.6792

Average non-exclusion probability (second parent): 0.4910

Average non-exclusion probability (parent pair): 0.2807

Average non-exclusion probability (identity): 0.1130

Average non-exclusion probability (sib identity): 0.4267

**** Locus sww-2662 ****

Allele Count Heterozygotes Homozygotes Frequency

178 1 1 0 0.0333

180 1 1 0 0.0333

181 6 4 1 0.2000

185 11 5 3 0.3667

186 2 2 0 0.0667

189 1 1 0 0.0333

191 1 1 0 0.0333

192 3 3 0 0.1000

195 3 3 0 0.1000

197 1 1 0 0.0333

Number of individuals typed: 15

Heterozygotes: 11

Homozygotes: 4

Number of alleles: 10

Observed heterozygosity: 0.7333

Expected heterozygosity: 0.8230

Polymorphic information content (PIC): 0.7737

Average non-exclusion probability (first parent): 0.5548

Average non-exclusion probability (second parent): 0.3771

Average non-exclusion probability (parent pair): 0.1852

Average non-exclusion probability (identity): 0.0637

Average non-exclusion probability (sib identity): 0.3681

**** Locus sww-1394 ****

Allele Count Heterozygotes Homozygotes Frequency

194 1 1 0 0.0313

204 1 1 0 0.0313

205 17 1 8 0.5313

206 1 1 0 0.0313

208 2 2 0 0.0625

211 4 4 0 0.1250

212 4 4 0 0.1250

217 2 2 0 0.0625

Number of individuals typed: 16

Heterozygotes: 8

Homozygotes: 8

Number of alleles: 8

Observed heterozygosity: 0.5000

Expected heterozygosity: 0.6976

Polymorphic information content (PIC): 0.6508

Average non-exclusion probability (first parent): 0.7095

Average non-exclusion probability (second parent): 0.5208

Average non-exclusion probability (parent pair): 0.3090

Average non-exclusion probability (identity): 0.1301

Average non-exclusion probability (sib identity): 0.4446

**** Locus sww-1615 ****

Allele Count Heterozygotes Homozygotes Frequency

185 1 1 0 0.0313

190 1 1 0 0.0313

193 1 1 0 0.0313

195 1 1 0 0.0313

197 1 1 0 0.0313

198 1 1 0 0.0313

199 2 2 0 0.0625

200 1 1 0 0.0313

201 1 1 0 0.0313

202 1 1 0 0.0313

204 3 3 0 0.0938

205 9 7 1 0.2813

207 2 2 0 0.0625

208 1 1 0 0.0313

210 3 3 0 0.0938

213 2 2 0 0.0625

216 1 1 0 0.0313

Number of individuals typed: 16

Heterozygotes: 15

Homozygotes: 1

Number of alleles: 17

Observed heterozygosity: 0.9375

Expected heterozygosity: 0.9093

Polymorphic information content (PIC): 0.8731

Average non-exclusion probability (first parent): 0.3677

Average non-exclusion probability (second parent): 0.2252

Average non-exclusion probability (parent pair): 0.0712

Average non-exclusion probability (identity): 0.0219

Average non-exclusion probability (sib identity): 0.3151

**** Locus sww-1622 ****

Allele Count Heterozygotes Homozygotes Frequency

233 7 7 0 0.2333

236 5 5 0 0.1667

237 7 5 1 0.2333

239 7 5 1 0.2333

242 2 2 0 0.0667

246 2 2 0 0.0667

Number of individuals typed: 15

Heterozygotes: 13

Homozygotes: 2

Number of alleles: 6

Observed heterozygosity: 0.8667

Expected heterozygosity: 0.8276

Polymorphic information content (PIC): 0.7697

Average non-exclusion probability (first parent): 0.5758

Average non-exclusion probability (second parent): 0.3979

Average non-exclusion probability (parent pair): 0.2184

Average non-exclusion probability (identity): 0.0703

Average non-exclusion probability (sib identity): 0.3676

**** Locus sww-1643 ****

Allele Count Heterozygotes Homozygotes Frequency

177 1 1 0 0.0357

184 1 1 0 0.0357

185 6 4 1 0.2143

186 3 1 1 0.1071

187 3 1 1 0.1071

188 2 0 1 0.0714

190 3 3 0 0.1071

191 2 0 1 0.0714

194 2 2 0 0.0714

204 1 1 0 0.0357

205 2 0 1 0.0714

207 2 0 1 0.0714

Number of individuals typed: 14

Heterozygotes: 7

Homozygotes: 7

Number of alleles: 12

Observed heterozygosity: 0.5000

Expected heterozygosity: 0.9233

Polymorphic information content (PIC): 0.8809

Average non-exclusion probability (first parent): 0.3607

Average non-exclusion probability (second parent): 0.2191

Average non-exclusion probability (parent pair): 0.0740

Average non-exclusion probability (identity): 0.0214

Average non-exclusion probability (sib identity): 0.3102

**** Locus sww-1667 ****

Allele Count Heterozygotes Homozygotes Frequency

211 14 0 7 0.4667

220 2 0 1 0.0667

224 4 2 1 0.1333

226 3 1 1 0.1000

227 1 1 0 0.0333

230 1 1 0 0.0333

231 3 1 1 0.1000

234 2 0 1 0.0667

Number of individuals typed: 15

Heterozygotes: 3

Homozygotes: 12

Number of alleles: 8

Observed heterozygosity: 0.2000

Expected heterozygosity: 0.7586

Polymorphic information content (PIC): 0.7102

Average non-exclusion probability (first parent): 0.6417

Average non-exclusion probability (second parent): 0.4542

Average non-exclusion probability (parent pair): 0.2464

Average non-exclusion probability (identity): 0.0942

Average non-exclusion probability (sib identity): 0.4069

**** Locus sww-1749 ****

Allele Count Heterozygotes Homozygotes Frequency

208 2 2 0 0.0625

209 4 4 0 0.1250

210 9 5 2 0.2813

211 4 0 2 0.1250

212 2 2 0 0.0625

213 7 5 1 0.2188

214 2 2 0 0.0625

216 1 1 0 0.0313

217 1 1 0 0.0313

Number of individuals typed: 16

Heterozygotes: 11

Homozygotes: 5

Number of alleles: 9

Observed heterozygosity: 0.6875

Expected heterozygosity: 0.8548

Polymorphic information content (PIC): 0.8077

Average non-exclusion probability (first parent): 0.5060

Average non-exclusion probability (second parent): 0.3349

Average non-exclusion probability (parent pair): 0.1569

Average non-exclusion probability (identity): 0.0500

Average non-exclusion probability (sib identity): 0.3484

**** Locus sww-1754 ****

Allele Count Heterozygotes Homozygotes Frequency

176 3 3 0 0.0938

182 7 3 2 0.2188

183 9 1 4 0.2813

184 9 3 3 0.2813

200 1 1 0 0.0313

203 1 1 0 0.0313

205 1 1 0 0.0313

207 1 1 0 0.0313

Number of individuals typed: 16

Heterozygotes: 7

Homozygotes: 9

Number of alleles: 8

Observed heterozygosity: 0.4375

Expected heterozygosity: 0.8065

Polymorphic information content (PIC): 0.7483

Average non-exclusion probability (first parent): 0.6003

Average non-exclusion probability (second parent): 0.4229

Average non-exclusion probability (parent pair): 0.2386

Average non-exclusion probability (identity): 0.0808

Average non-exclusion probability (sib identity): 0.3796

**** Locus sww-1761 ****

Allele Count Heterozygotes Homozygotes Frequency

201 1 1 0 0.0313

203 2 0 1 0.0625

204 1 1 0 0.0313

205 1 1 0 0.0313

207 1 1 0 0.0313

208 3 3 0 0.0938

209 1 1 0 0.0313

210 2 2 0 0.0625

211 5 5 0 0.1563

212 4 4 0 0.1250

213 4 4 0 0.1250

214 1 1 0 0.0313

216 3 3 0 0.0938

218 2 2 0 0.0625

223 1 1 0 0.0313

Number of individuals typed: 16

Heterozygotes: 15

Homozygotes: 1

Number of alleles: 15

Observed heterozygosity: 0.9375

Expected heterozygosity: 0.9375

Polymorphic information content (PIC): 0.9011

Average non-exclusion probability (first parent): 0.3129

Average non-exclusion probability (second parent): 0.1852

Average non-exclusion probability (parent pair): 0.0552

Average non-exclusion probability (identity): 0.0156

Average non-exclusion probability (sib identity): 0.2998

**** Locus sww-1795 ****

Allele Count Heterozygotes Homozygotes Frequency

214 1 1 0 0.0333

215 1 1 0 0.0333

218 2 2 0 0.0667

220 3 3 0 0.1000

221 7 7 0 0.2333

222 1 1 0 0.0333

223 1 1 0 0.0333

224 8 8 0 0.2667

225 4 4 0 0.1333

227 2 2 0 0.0667

Number of individuals typed: 15

Heterozygotes: 15

Homozygotes: 0

Number of alleles: 10

Observed heterozygosity: 1.0000

Expected heterozygosity: 0.8621

Polymorphic information content (PIC): 0.8140

Average non-exclusion probability (first parent): 0.4934

Average non-exclusion probability (second parent): 0.3245

Average non-exclusion probability (parent pair): 0.1480

Average non-exclusion probability (identity): 0.0471

Average non-exclusion probability (sib identity): 0.3451

**** Locus sww-1813 ****

Allele Count Heterozygotes Homozygotes Frequency

149 3 3 0 0.1000

153 1 1 0 0.0333

154 3 3 0 0.1000

159 6 6 0 0.2000

170 8 0 4 0.2667

172 2 2 0 0.0667

173 2 0 1 0.0667

174 2 2 0 0.0667

175 2 0 1 0.0667

182 1 1 0 0.0333

Number of individuals typed: 15

Heterozygotes: 9

Homozygotes: 6

Number of alleles: 10

Observed heterozygosity: 0.6000

Expected heterozygosity: 0.8782

Polymorphic information content (PIC): 0.8330

Average non-exclusion probability (first parent): 0.4587

Average non-exclusion probability (second parent): 0.2952

Average non-exclusion probability (parent pair): 0.1246

Average non-exclusion probability (identity): 0.0387

Average non-exclusion probability (sib identity): 0.3352

**** Locus sww-1889 ****

Allele Count Heterozygotes Homozygotes Frequency

211 2 2 0 0.0625

212 1 1 0 0.0313

215 22 6 8 0.6875

218 7 3 2 0.2188

Number of individuals typed: 16

Heterozygotes: 6

Homozygotes: 10

Number of alleles: 4

Observed heterozygosity: 0.3750

Expected heterozygosity: 0.4899

Polymorphic information content (PIC): 0.4243

Average non-exclusion probability (first parent): 0.8838

Average non-exclusion probability (second parent): 0.7489

Average non-exclusion probability (parent pair): 0.6026

Average non-exclusion probability (identity): 0.3264

Average non-exclusion probability (sib identity): 0.5943

**** Locus sww-1969 ****

Allele Count Heterozygotes Homozygotes Frequency

206 1 1 0 0.0357

207 1 1 0 0.0357

213 1 1 0 0.0357

214 6 4 1 0.2143

216 1 1 0 0.0357

217 2 2 0 0.0714

220 2 2 0 0.0714

222 2 2 0 0.0714

224 1 1 0 0.0357

225 1 1 0 0.0357

226 4 4 0 0.1429

228 2 2 0 0.0714

231 1 1 0 0.0357

234 3 3 0 0.1071

Number of individuals typed: 14

Heterozygotes: 13

Homozygotes: 1

Number of alleles: 14

Observed heterozygosity: 0.9286

Expected heterozygosity: 0.9259

Polymorphic information content (PIC): 0.8841

Average non-exclusion probability (first parent): 0.3509

Average non-exclusion probability (second parent): 0.2125

Average non-exclusion probability (parent pair): 0.0692

Average non-exclusion probability (identity): 0.0202

Average non-exclusion probability (sib identity): 0.3086

**** Locus sww-2034 ****

Allele Count Heterozygotes Homozygotes Frequency

226 1 1 0 0.0333

227 10 10 0 0.3333

228 2 2 0 0.0667

230 10 10 0 0.3333

231 3 3 0 0.1000

236 2 2 0 0.0667

239 1 1 0 0.0333

244 1 1 0 0.0333

Number of individuals typed: 15

Heterozygotes: 15

Homozygotes: 0

Number of alleles: 8

Observed heterozygosity: 1.0000

Expected heterozygosity: 0.7816

Polymorphic information content (PIC): 0.7206

Average non-exclusion probability (first parent): 0.6297

Average non-exclusion probability (second parent): 0.4521

Average non-exclusion probability (parent pair): 0.2602

Average non-exclusion probability (identity): 0.0947

Average non-exclusion probability (sib identity): 0.3959

**** Locus sww-2070 ****

Allele Count Heterozygotes Homozygotes Frequency

176 10 10 0 0.3125

181 3 3 0 0.0938

213 3 1 1 0.0938

216 1 1 0 0.0313

218 2 2 0 0.0625

219 13 11 1 0.4063

Number of individuals typed: 16

Heterozygotes: 14

Homozygotes: 2

Number of alleles: 6

Observed heterozygosity: 0.8750

Expected heterozygosity: 0.7379

Polymorphic information content (PIC): 0.6705

Average non-exclusion probability (first parent): 0.6909

Average non-exclusion probability (second parent): 0.5166

Average non-exclusion probability (parent pair): 0.3285

Average non-exclusion probability (identity): 0.1257

Average non-exclusion probability (sib identity): 0.4240

**** Locus sww-2167 ****

Allele Count Heterozygotes Homozygotes Frequency

174 1 1 0 0.0313

176 1 1 0 0.0313

177 1 1 0 0.0313

178 5 5 0 0.1563

179 1 1 0 0.0313

180 2 2 0 0.0625

181 9 9 0 0.2813

182 2 2 0 0.0625

184 5 5 0 0.1563

187 5 5 0 0.1563

Number of individuals typed: 16

Heterozygotes: 16

Homozygotes: 0

Number of alleles: 10

Observed heterozygosity: 1.0000

Expected heterozygosity: 0.8629

Polymorphic information content (PIC): 0.8171

Average non-exclusion probability (first parent): 0.4894

Average non-exclusion probability (second parent): 0.3207

Average non-exclusion probability (parent pair): 0.1454

Average non-exclusion probability (identity): 0.0458

Average non-exclusion probability (sib identity): 0.3435

**** Locus sww-2235 ****

Allele Count Heterozygotes Homozygotes Frequency

214 24 2 11 0.9231

220 1 1 0 0.0385

226 1 1 0 0.0385

Number of individuals typed: 13

Heterozygotes: 2

Homozygotes: 11

Number of alleles: 3

Observed heterozygosity: 0.1538

Expected heterozygosity: 0.1508

Polymorphic information content (PIC): 0.1399

Average non-exclusion probability (first parent): 0.9895

Average non-exclusion probability (second parent): 0.9262

Average non-exclusion probability (parent pair): 0.8627

Average non-exclusion probability (identity): 0.7361

Average non-exclusion probability (sib identity): 0.8615

**** Locus sww-223 ****

Allele Count Heterozygotes Homozygotes Frequency

204 1 1 0 0.0313

210 1 1 0 0.0313

215 5 5 0 0.1563

220 5 5 0 0.1563

223 4 4 0 0.1250

225 16 16 0 0.5000

Number of individuals typed: 16

Heterozygotes: 16

Homozygotes: 0

Number of alleles: 6

Observed heterozygosity: 1.0000

Expected heterozygosity: 0.7056

Polymorphic information content (PIC): 0.6474

Average non-exclusion probability (first parent): 0.7186

Average non-exclusion probability (second parent): 0.5371

Average non-exclusion probability (parent pair): 0.3409

Average non-exclusion probability (identity): 0.1363

Average non-exclusion probability (sib identity): 0.4423

**** Locus sww-2320 ****

Allele Count Heterozygotes Homozygotes Frequency

189 1 1 0 0.0313

190 1 1 0 0.0313

193 1 1 0 0.0313

194 2 2 0 0.0625

195 1 1 0 0.0313

196 15 5 5 0.4688

198 4 4 0 0.1250

199 2 2 0 0.0625

202 4 4 0 0.1250

206 1 1 0 0.0313

Number of individuals typed: 16

Heterozygotes: 11

Homozygotes: 5

Number of alleles: 10

Observed heterozygosity: 0.6875

Expected heterozygosity: 0.7601

Polymorphic information content (PIC): 0.7156

Average non-exclusion probability (first parent): 0.6319

Average non-exclusion probability (second parent): 0.4443

Average non-exclusion probability (parent pair): 0.2336

Average non-exclusion probability (identity): 0.0902

Average non-exclusion probability (sib identity): 0.4044

**** Locus sww-2368 ****

Allele Count Heterozygotes Homozygotes Frequency

220 8 2 3 0.2500

223 1 1 0 0.0313

224 2 2 0 0.0625

227 1 1 0 0.0313

231 8 2 3 0.2500

232 8 6 1 0.2500

233 2 2 0 0.0625

234 2 0 1 0.0625

Number of individuals typed: 16

Heterozygotes: 8

Homozygotes: 8

Number of alleles: 8

Observed heterozygosity: 0.5000

Expected heterozygosity: 0.8246

Polymorphic information content (PIC): 0.7701

Average non-exclusion probability (first parent): 0.5684

Average non-exclusion probability (second parent): 0.3921

Average non-exclusion probability (parent pair): 0.2094

Average non-exclusion probability (identity): 0.0692

Average non-exclusion probability (sib identity): 0.3679

**** Locus sww-2376 ****

Allele Count Heterozygotes Homozygotes Frequency

204 1 1 0 0.0313

209 3 3 0 0.0938

211 1 1 0 0.0313

212 1 1 0 0.0313

213 16 8 4 0.5000

216 7 7 0 0.2188

217 1 1 0 0.0313

219 1 1 0 0.0313

223 1 1 0 0.0313

Number of individuals typed: 16

Heterozygotes: 12

Homozygotes: 4

Number of alleles: 9

Observed heterozygosity: 0.7500

Expected heterozygosity: 0.7097

Polymorphic information content (PIC): 0.6547

Average non-exclusion probability (first parent): 0.7034

Average non-exclusion probability (second parent): 0.5214

Average non-exclusion probability (parent pair): 0.3176

Average non-exclusion probability (identity): 0.1304

Average non-exclusion probability (sib identity): 0.4389

**** Locus sww-2377 ****

Allele Count Heterozygotes Homozygotes Frequency

276 30 2 14 0.9375

280 1 1 0 0.0313

281 1 1 0 0.0313

Number of individuals typed: 16

Heterozygotes: 2

Homozygotes: 14

Number of alleles: 3

Observed heterozygosity: 0.1250

Expected heterozygosity: 0.1230

Polymorphic information content (PIC): 0.1157

Average non-exclusion probability (first parent): 0.9929

Average non-exclusion probability (second parent): 0.9396

Average non-exclusion probability (parent pair): 0.8860

Average non-exclusion probability (identity): 0.7793

Average non-exclusion probability (sib identity): 0.8853

**** Locus sww-2387 ****

Allele Count Heterozygotes Homozygotes Frequency

238 9 1 4 0.3000

239 7 1 3 0.2333

245 14 2 6 0.4667

Number of individuals typed: 15

Heterozygotes: 2

Homozygotes: 13

Number of alleles: 3

Observed heterozygosity: 0.1333

Expected heterozygosity: 0.6598

Polymorphic information content (PIC): 0.5651

Average non-exclusion probability (first parent): 0.7966

Average non-exclusion probability (second parent): 0.6507

Average non-exclusion probability (parent pair): 0.5008

Average non-exclusion probability (identity): 0.2039

Average non-exclusion probability (sib identity): 0.4821

**** Locus sww-2503 ****

Allele Count Heterozygotes Homozygotes Frequency

198 1 1 0 0.0313

199 1 1 0 0.0313

200 2 2 0 0.0625

201 1 1 0 0.0313

204 8 4 2 0.2500

205 1 1 0 0.0313

206 5 5 0 0.1563

208 1 1 0 0.0313

373 12 0 6 0.3750

Number of individuals typed: 16

Heterozygotes: 8

Homozygotes: 8

Number of alleles: 9

Observed heterozygosity: 0.5000

Expected heterozygosity: 0.7883

Polymorphic information content (PIC): 0.7321

Average non-exclusion probability (first parent): 0.6162

Average non-exclusion probability (second parent): 0.4372

Average non-exclusion probability (parent pair): 0.2449

Average non-exclusion probability (identity): 0.0874

Average non-exclusion probability (sib identity): 0.3900

**** Locus sww-2527 ****

Allele Count Heterozygotes Homozygotes Frequency

180 3 3 0 0.0938

192 1 1 0 0.0313

195 3 3 0 0.0938

196 6 2 2 0.1875

197 1 1 0 0.0313

198 7 5 1 0.2188

199 1 1 0 0.0313

200 6 4 1 0.1875

202 1 1 0 0.0313

204 2 2 0 0.0625

205 1 1 0 0.0313

Number of individuals typed: 16

Heterozygotes: 12

Homozygotes: 4

Number of alleles: 11

Observed heterozygosity: 0.7500

Expected heterozygosity: 0.8831

Polymorphic information content (PIC): 0.8395

Average non-exclusion probability (first parent): 0.4484

Average non-exclusion probability (second parent): 0.2871

Average non-exclusion probability (parent pair): 0.1209

Average non-exclusion probability (identity): 0.0368

Average non-exclusion probability (sib identity): 0.3315

**** Locus sww-2532 ****

Allele Count Heterozygotes Homozygotes Frequency

178 2 0 1 0.0714

179 1 1 0 0.0357

182 1 1 0 0.0357

199 13 3 5 0.4643

203 3 3 0 0.1071

204 1 1 0 0.0357

208 6 2 2 0.2143

214 1 1 0 0.0357

Number of individuals typed: 14

Heterozygotes: 6

Homozygotes: 8

Number of alleles: 8

Observed heterozygosity: 0.4286

Expected heterozygosity: 0.7434

Polymorphic information content (PIC): 0.6854

Average non-exclusion probability (first parent): 0.6717

Average non-exclusion probability (second parent): 0.4888

Average non-exclusion probability (parent pair): 0.2870

Average non-exclusion probability (identity): 0.1116

Average non-exclusion probability (sib identity): 0.4195

**** Locus sww-2545 ****

Allele Count Heterozygotes Homozygotes Frequency

180 1 1 0 0.0313

183 2 2 0 0.0625

184 1 1 0 0.0313

185 2 2 0 0.0625

187 21 5 8 0.6563

188 3 3 0 0.0938

190 1 1 0 0.0313

192 1 1 0 0.0313

Number of individuals typed: 16

Heterozygotes: 8

Homozygotes: 8

Number of alleles: 8

Observed heterozygosity: 0.5000

Expected heterozygosity: 0.5665

Polymorphic information content (PIC): 0.5309

Average non-exclusion probability (first parent): 0.8181

Average non-exclusion probability (second parent): 0.6349

Average non-exclusion probability (parent pair): 0.4260

Average non-exclusion probability (identity): 0.2215

Average non-exclusion probability (sib identity): 0.5310

**** Locus sww-2578 ****

Allele Count Heterozygotes Homozygotes Frequency

215 16 12 2 0.5333

217 2 2 0 0.0667

222 1 1 0 0.0333

241 11 9 1 0.3667

Number of individuals typed: 15

Heterozygotes: 12

Homozygotes: 3

Number of alleles: 4

Observed heterozygosity: 0.8000

Expected heterozygosity: 0.5954

Polymorphic information content (PIC): 0.4944

Average non-exclusion probability (first parent): 0.8292

Average non-exclusion probability (second parent): 0.7028

Average non-exclusion probability (parent pair): 0.5559

Average non-exclusion probability (identity): 0.2613

Average non-exclusion probability (sib identity): 0.5275

**** Locus sww-333 ****

Allele Count Heterozygotes Homozygotes Frequency

146 2 2 0 0.0625

147 1 1 0 0.0313

149 12 4 4 0.3750

150 1 1 0 0.0313

151 4 4 0 0.1250

156 11 11 0 0.3438

157 1 1 0 0.0313

Number of individuals typed: 16

Heterozygotes: 12

Homozygotes: 4

Number of alleles: 7

Observed heterozygosity: 0.7500

Expected heterozygosity: 0.7419

Polymorphic information content (PIC): 0.6736

Average non-exclusion probability (first parent): 0.6862

Average non-exclusion probability (second parent): 0.5131

Average non-exclusion probability (parent pair): 0.3256

Average non-exclusion probability (identity): 0.1242

Average non-exclusion probability (sib identity): 0.4217

**** Locus sww-387 ****

Allele Count Heterozygotes Homozygotes Frequency

154 23 5 9 0.7188

155 1 1 0 0.0313

156 2 0 1 0.0625

157 1 1 0 0.0313

159 5 5 0 0.1563

Number of individuals typed: 16

Heterozygotes: 6

Homozygotes: 10

Number of alleles: 5

Observed heterozygosity: 0.3750

Expected heterozygosity: 0.4677

Polymorphic information content (PIC): 0.4215

Average non-exclusion probability (first parent): 0.8901

Average non-exclusion probability (second parent): 0.7413

Average non-exclusion probability (parent pair): 0.5795

Average non-exclusion probability (identity): 0.3307

Average non-exclusion probability (sib identity): 0.6061

**** Locus sww-389 ****

Allele Count Heterozygotes Homozygotes Frequency

235 2 0 1 0.0833

236 2 0 1 0.0833

238 2 0 1 0.0833

240 2 0 1 0.0833

241 10 0 5 0.4167

242 4 0 2 0.1667

244 2 0 1 0.0833

Number of individuals typed: 12

Heterozygotes: 0

Homozygotes: 12

Number of alleles: 7

Observed heterozygosity: 0.0000

Expected heterozygosity: 0.7971

Polymorphic information content (PIC): 0.7393

Average non-exclusion probability (first parent): 0.6070

Average non-exclusion probability (second parent): 0.4231

Average non-exclusion probability (parent pair): 0.2244

Average non-exclusion probability (identity): 0.0803

Average non-exclusion probability (sib identity): 0.3881

**** Locus sww-463 ****

Allele Count Heterozygotes Homozygotes Frequency

253 11 5 3 0.3438

254 7 1 3 0.2188

255 2 0 1 0.0625

256 5 3 1 0.1563

258 3 3 0 0.0938

261 2 2 0 0.0625

273 2 0 1 0.0625

Number of individuals typed: 16

Heterozygotes: 7

Homozygotes: 9

Number of alleles: 7

Observed heterozygosity: 0.4375

Expected heterozygosity: 0.8145

Polymorphic information content (PIC): 0.7615

Average non-exclusion probability (first parent): 0.5798

Average non-exclusion probability (second parent): 0.4009

Average non-exclusion probability (parent pair): 0.2134

Average non-exclusion probability (identity): 0.0720

Average non-exclusion probability (sib identity): 0.3735

**** Locus sww-532 ****

Allele Count Heterozygotes Homozygotes Frequency

208 1 1 0 0.0333

215 1 1 0 0.0333

217 7 7 0 0.2333

219 1 1 0 0.0333

225 10 8 1 0.3333

227 1 1 0 0.0333

228 1 1 0 0.0333

229 2 2 0 0.0667

230 5 3 1 0.1667

231 1 1 0 0.0333

Number of individuals typed: 15

Heterozygotes: 13

Homozygotes: 2

Number of alleles: 10

Observed heterozygosity: 0.8667

Expected heterozygosity: 0.8230

Polymorphic information content (PIC): 0.7699

Average non-exclusion probability (first parent): 0.5630

Average non-exclusion probability (second parent): 0.3865

Average non-exclusion probability (parent pair): 0.1986

Average non-exclusion probability (identity): 0.0675

Average non-exclusion probability (sib identity): 0.3691

**** Locus sww-556 ****

Allele Count Heterozygotes Homozygotes Frequency

110 2 0 1 0.0625

112 3 3 0 0.0938

120 3 3 0 0.0938

121 2 2 0 0.0625

125 1 1 0 0.0313

126 3 3 0 0.0938

128 1 1 0 0.0313

132 1 1 0 0.0313

133 9 9 0 0.2813

134 2 2 0 0.0625

135 1 1 0 0.0313

136 2 2 0 0.0625

141 2 2 0 0.0625

Number of individuals typed: 16

Heterozygotes: 15

Homozygotes: 1

Number of alleles: 13

Observed heterozygosity: 0.9375

Expected heterozygosity: 0.8992

Polymorphic information content (PIC): 0.8610

Average non-exclusion probability (first parent): 0.3979

Average non-exclusion probability (second parent): 0.2471

Average non-exclusion probability (parent pair): 0.0871

Average non-exclusion probability (identity): 0.0267

Average non-exclusion probability (sib identity): 0.3211

**** Locus sww-573 ****

Allele Count Heterozygotes Homozygotes Frequency

229 13 7 3 0.4063

231 1 1 0 0.0313

232 1 1 0 0.0313

235 3 1 1 0.0938

237 1 1 0 0.0313

240 5 3 1 0.1563

241 1 1 0 0.0313

242 2 2 0 0.0625

243 2 2 0 0.0625

246 1 1 0 0.0313

247 2 2 0 0.0625

Number of individuals typed: 16

Heterozygotes: 11

Homozygotes: 5

Number of alleles: 11

Observed heterozygosity: 0.6875

Expected heterozygosity: 0.8105

Polymorphic information content (PIC): 0.7670

Average non-exclusion probability (first parent): 0.5607

Average non-exclusion probability (second parent): 0.3802

Average non-exclusion probability (parent pair): 0.1804

Average non-exclusion probability (identity): 0.0644

Average non-exclusion probability (sib identity): 0.3735

**** Locus SWW-611 ****

Allele Count Heterozygotes Homozygotes Frequency

230 2 0 1 0.0625

233 5 1 2 0.1563

235 4 2 1 0.1250

236 18 4 7 0.5625

239 2 2 0 0.0625

242 1 1 0 0.0313

Number of individuals typed: 16

Heterozygotes: 5

Homozygotes: 11

Number of alleles: 6

Observed heterozygosity: 0.3125

Expected heterozygosity: 0.6552

Polymorphic information content (PIC): 0.6024

Average non-exclusion probability (first parent): 0.7600

Average non-exclusion probability (second parent): 0.5784

Average non-exclusion probability (parent pair): 0.3778

Average non-exclusion probability (identity): 0.1658

Average non-exclusion probability (sib identity): 0.4741

********************************************************************************

**NOTE**: For each locus in the allele frequency analysis, a table of alleles is shown containing the following information:

Allele: Name of the allele.

Count: Number of occurrences of the allele in the genotype file.

Heterozygotes: Number of individuals in the genotype file heterozygous for the allele.

Homozygotes: Number of individuals in the genotype file homozygous for the allele.

Frequency: Number of occurrences of the allele divided by the total number of alleles.
